# Art’s hidden topology: A window into human perception

**DOI:** 10.1371/journal.pcbi.1014156

**Published:** 2026-05-14

**Authors:** Emil Dmitruk, Beata Bajno, Lidia Kot, Joanna Dreszer, Bibianna Bałaj, Ewa Ratajczak, Marcin Hajnowski, Romuald A. Janik, Marek Kuś, Shabnam N. Kadir, Jacek Rogala

**Affiliations:** 1 UH Biocomputation Research Group, Department of Computer Science, University of Hertfordshire, Hatfield, United Kingdom; 2 Independent Artist, Warsaw, Poland; 3 In Situ Contemporary Art Foundation, International Laboratory of Culture, Sokołowsko, Poland; 4 Institute of Psychology, Faculty of Philosophy and Social Sciences, Nicolaus Copernicus University in Toruń, Toruń, Poland; 5 Institute of Theoretical Physics and Mark Kac Center for Complex Systems Research, Jagiellonian University, Kraków, Poland; 6 Center for Theoretical Physics, Polish Academy of Sciences, Warsaw, Poland; 7 Center of Trustworthy AI for Life Sciences – International Research Agendas Programme, University of Warsaw, Warsaw, Poland; Pantheon-Sorbonne University: Universite Paris 1 Pantheon-Sorbonne, FRANCE

## Abstract

Generations of researchers have sought a link between features of an artistic image and the audience’s experience. However, a direct link between the properties of an image and the responses evoked has still not been established. Given the importance of shape to human perception and artistic creation, it can be assumed that one of the most important aspects of an artistic image is the use of different visual structures. We show that a method from the field of computational topology, persistent homology, can be used to analyse properties of image structures and composition at multiple scales. In order to determine the reliability of this method as a tool for analysing visual artworks, we analysed two different sets of abstract paintings that revealed significant discrepancies in the eye tracking, electrical brain activity and the subjective experience of viewers. Our research showed that our newly developed method not only clearly distinguished between two sets of images but also allowed us to map topological features onto gaze fixation heat maps. Furthermore, the extent to which various artistic images violate a topological duality (Alexander duality) is significantly different from that of pseudo-art. It is intriguing that a diverse group of eminent abstract artists seem to favour a special rate of violation close to a specific value.

## Author summary

For decades, scientists have tried to understand why certain works of art move us more than others. Yet, directly connecting what is in an image to how it makes us feel has proven elusive. Since shapes and visual patterns play such a central role in both art and how we see the world, we explored a new angle: using advanced mathematical tools from topology to reveal hidden structures in images. By applying a method called persistent homology, we examined how artworks are built up from layers of visual shapes at different scales. We then tested this approach by studying people’s eye movements as they viewed two sets of abstract paintings. Remarkably, our method did not just separate the two groups of images clearly – it also let us create maps showing exactly where viewers looked, linked to the underlying shapes in the artwork. We also reveal that artists appear to be following a type of ‘golden rule’ when it comes to framing their compositions using shapes.

## 1 Introduction

Psychological studies suggest that the experience elicited by a work of art can evolve as the viewer deciphers the message encoded by the artist. This process resembles the transmission of information from a sender to a receiver through a communication channel—here, the artwork itself—as outlined in Shannon’s information theory [1,2].

We proposed that this flow of information is critical for expressing artistic intent [2] and shaping the viewer’s perceptual and emotional engagement. To examine the potential for informational transfer through visual art, we compared audience responses to two types of stimuli: intentionally crafted abstract artistic images conveying an original artist’s message, and pseudo-artistic abstract images generated by randomly perturbed generative neural networks lacking deliberate content. We hypothesized that the presence of a purposeful and consistent communicative intent in the artist-curated images, as opposed to the incoherent nature of the randomly generated pseudo-art counterparts, would elicit systematically different responses at both the behavioural and the aesthetic experience level, regardless of the mode of audience exposure. Building on the work of [1] and [2], we also anticipated systematic structural differences in the formal properties of the images.

However, although it is acknowledged that works of art often reflect the statistical regularities present in natural scenes, a direct correlation between these statistics and the experience evoked by art is weak or at most, moderate [3].

Concurrently, studies in both human and non-human primates (for example macaques) have revealed that higher-order brain regions are more responsive to holistic shapes than to basic visual elements, such as edges, used to define the shape [4–7].

The ability to perceive and recognise shapes and objects is not only fundamental to our interaction with the world but also crucial for artists in depicting scenes or objects accurately. This skill is particularly important for individuals with advanced drawing and painting abilities. Research by Robles et al. [8] demonstrates a significant correlation between the accuracy of shape judgement in skilled individuals and their drawing precision. This suggests that enhanced abilities in realistic drawing are likely linked to a more precise perception of shapes.

Given the importance of shape and object perception in artistic creation, it is reasonable to postulate that these elements are also vital tools for artists in conveying impressions to their audience. This concept is eloquently explored in Arnheim’s seminal work ‘Art and Visual Perception’, where he delves into the role of visual perception in art and how artists use shapes, among other elements, to create specific psychological effects in viewers. He posits that artists are keenly aware of the impact of certain shapes in crafting these effects [9].

We believe that one of the most crucial aspects of an artistic image, especially in terms of viewer experience, is indeed the use of various visual structures. In mathematics, topology offers a quantitative framework for analyzing the qualitative properties of shapes that remain invariant under continuous deformations – such as bending and stretching (cutting and glueing are forbidden). In this context, persistent homology [10] is particularly compelling, as it captures the topological features of an image that emerge across multiple spatial scales by computing homology classes over a hierarchy of combinatorial representations derived from the image. This makes it especially well-suited for the characterization and comparison of structural patterns within image data.

This mathematical method builds up abstract structures in very much the same way that an artist composes the shapes of a painting from colours, shades and edges. The method explores qualitative questions about spaces: Are there distinctive shapes, such as connected components, holes, and voids? How many shapes are there? Are there any holes? How many holes are there? It is, therefore, a useful tool that enables us to quantify the qualitative features of an abstract image to which people are drawn. The appeal of focusing on topological properties lies also in their independence from arbitrarily chosen coordinates and metric properties of the objects being perceived. Persistent homology operates on multiple scales and dimensions, by way of enabling not only the detection of less salient, less noticeable structures, but also the disclosure of detailed properties of the image that commonly used statistical image properties are unable to capture. Unlike figurative art, abstract art does not consist of identifiable objects that can be given clear labels; this detailed topological decomposition of the artworks in terms of shapes enables us to analyse how viewers mentally process a composition comprised of those abstract shapes.

In this study, we present findings from our psychophysiological experiment with a particular focus on the relationship between topological image properties and human perceptual experience. To achieve this, we first compared the topologically derived features of digital representations of images from both exhibitions. We then examined how these features related to participants’ eye movement patterns and subjective experience. We also compared the insights gained through persistent homology with those obtained using conventional statistical image descriptors. Finally, in the last step of our investigations, we compared topological features between artistic images originating from different abstract artists and those created by a randomly perturbed generative adversarial network. This methodological comparison is aimed at evaluating the effectiveness of persistent homology in capturing the perceptual complexity involved in the experience of visual art.

## 2 Materials and methods

### 2.1 Ethics statement

The Ethical approval was issued by the Research Ethics Committee at the Nicolaus Copernicus University in Toruń - decision number 16/2021/FT. All participants gave written consent before the experiment began.

### 2.2 General information

Both categories of abstract images (one created by an artist and the other by a perturbed generative adversarial network) were presented in two settings: (1) as part of a regular art gallery exhibition and (2) in a controlled laboratory environment via computer display. To evaluate potential differences in viewers’ responses across behavioural and aesthetic dimensions, we collected and analysed data from eye-tracking, self-reported flow questionnaires, and electroencephalography (EEG) recordings obtained while participants viewed the images.

Because the study incorporated a regularly scheduled art exhibition in a gallery and reused the same exhibition hall for the second condition (also presented to the general public as an art exhibition), it was not feasible to design the experiment so that each participant could view both exhibitions in a counterbalanced order. Accordingly, we recruited two groups of participants matched for personality and socioeconomic status. Each group was exposed to only one of the two exhibitions in two sessions (separated by a one-week interval).

To ensure an unbiased comparison, we intentionally avoided direct comparisons between the two experimental settings, namely the laboratory and the gallery. To clearly indicate which measurements were conducted in each setting, we introduce two separate subsections titled “In the gallery” and “In the laboratory” in the “Materials and methods” section and maintain this distinction further in the Results.

Prior to the main experiment, we conducted an initial assessment to rule out any baseline differences in aesthetic flow tendency and EEG activity. This involved administering flow‑propensity questionnaires and recording EEG resting state activity for both groups before commencing the gallery and laboratory investigations.

Both groups of participants were under the impression that they were viewing artworks made by a real artist for a solo exhibition. In the following sections, we describe the origin of the artistic images and the method used to produce the pseudo-artistic images. Next, we describe an experimental procedure designed to collect eye-tracking data. Finally, we describe how tools from persistent homology were used to examine differences between what participants looked at and what they neglected to look at.

### 2.3 Images and preprocessing

#### 2.3.1 Art images.

To ensure a meaningful and consistent artistic message, we chose a planned solo exhibition of Polish visual artist Lidia Kot [11], dedicated to the colour black and presented at the Wozownia Art Gallery, Toruń, Poland. The artistic images used in the laboratory condition were digital copies of the original works on display in the gallery. According to the artist, these works emerged from an “unrestricted creative process”, resulting in a collection of 12 colour art prints prepared specifically for the exhibition. Each artwork shared identical dimensions, 50 cm×100 cm (*W* × *H*). All were artistic fine prints printed using an EPSON STYLUS PRO 11880 printer with an Epson Micro Piezo TFP printhead and Epson UltraChrome K3 ink with Vivid Magenta technology (print resolution: 2880 × 1440 dpi; minimum droplet size: 3.5 pl) on Canson Rag Photographique 210 gsm paper.

**Fig 1:**
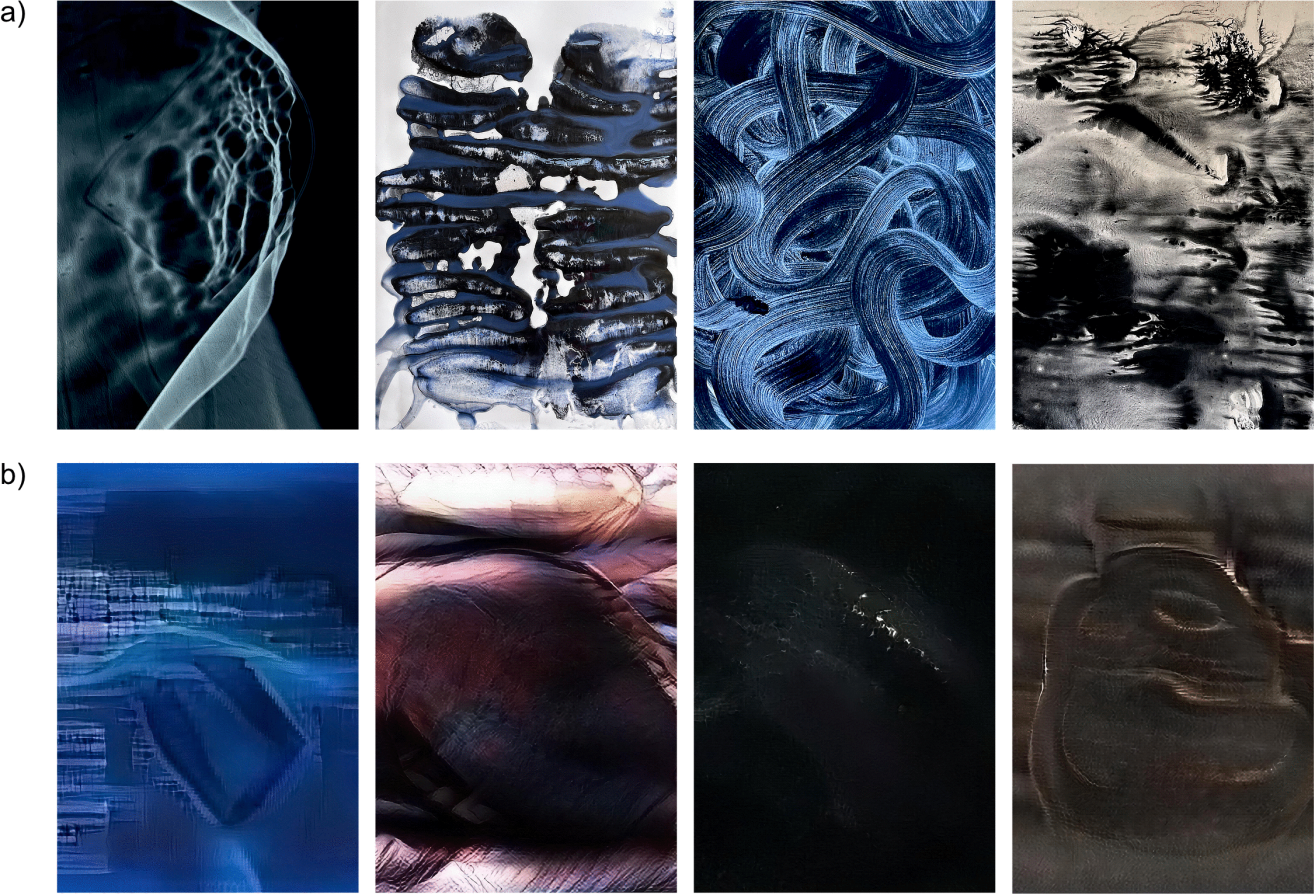
Samples of artistic images [11] (a) and pseudo-artistic images (b). The titles, from left to right, are: **(a)** ‘Black holes of memory’ (orig. ‘Czarne dziury pamięci’); ‘Lungs of Blackness’ (orig. ‘Płuca czerni’); ‘Ear of Blackness’ (orig. ‘Ucho czerni’); ‘Guts of Blackness’ (orig. ‘Jelita czerni’). **(b)** ‘Vibrations of time’ (orig. ‘Wibracje czasu’); ‘The Inside’ (orig. ‘Wnętrze’); ‘Cold fire’ (orig. ‘Zimny ognień’); orig. ‘Everything is a thing and nothing is everything’.

Examples of the artworks are shown in Fig 1a, and digital copies of all original artworks used in the study are available in S3 Fig.

#### 2.3.2 Pseudo-art images.

The second exhibition was structured to minimise any potential human involvement that might convey information or intentionality. As the information embedded in art and its reception might be implicit [2], it is difficult, if not impossible, to identify which visual properties communicate meaning or reflect the artist’s intent. Consequently, we refrained from manipulating original artworks and instead employed artificially generated images created through processes specifically designed to minimise the presence of consistent or coherent information. To achieve this, we utilised randomly selected categories from a randomly perturbed BigGAN network [12], generating abstract visual forms derived from initially photorealistic image categories.

In order to ensure that the generated images had an abstract non-figurative character, the weights of all layers in Block 2, as well as those of a layer in the “self-attention” block of the BigGAN network (see Fig 1 in [13]), were substituted by random numbers. All the remaining weights remained unchanged. In this way, we constructed 500 different neural networks by drawing 500 random vectors of neural network weights, which gave us a sufficient variety of image distortion types or styles. Subsequently, a subset of 480 ImageNet categories was specifically chosen to exclude any that featured animals or human faces to ensure a strictly non-figurative character for the pseudo-artistic images. Then, for each of the 500 network modifications, images were generated using 9 categories randomly selected from this ‘non-figurative’ subset. In this way, we obtained 4500 neural network-generated images of size 256×256. The images generated by the perturbed neural networks were then enlarged to a 2048×2048 resolution by first reducing colour noise in Photoshop (Adobe Systems Inc., Adobe Photoshop 2022) and subsequently upscaling using an SRGAN [14] super-resolution neural network.

Examples of the generated images are shown in Fig 1b, digital copies of all artificially generated images used in the study are available in S4 Fig.

For the purpose of this study, the original artworks were downscaled to a resolution 1434×2048 (*W* × *H*) using Photoshop. All pseudo-artistic images were rescaled to match the resolution of the artistic images. Next, in order to mitigate differences in audience response arising merely from large disparities in brightness or colour, while minimizing human input, each of the 4500 artificially generated images was compared with each of the artist’s 12 artworks using Matlab’s (The MathWorks, Inc.) *imabsdiff* function. The *imabsdiff* function compares two images of the same size by computing the absolute value of the difference between the pixel intensities of the two images (resulting in a matrix of absolute pixel value differences). Next, this matrix was averaged to obtain a single value for each pair of compared images (by comparing each of 12 images with each of 4500 generated images, resulting in 54000 comparisons). The averaged differences of all comparisons were then placed in ascending order, and the 12 images with the smallest difference were selected as the set of pseudo-artistic images used in the present study.

Selected pseudo-artistic images were then printed to the same size as the artistic ones in the same print shop using exactly the same technology and paper. The titles for the pseudo-artistic exhibition were generated using and publicly available painting titles from exhibitions held at the Wozownia Art Gallery. All texts were created using GPT-3 software (beta.openai.com) and initially generated in English before being translated into Polish via Google Translate. From the pool of generated titles, 12 were randomly selected using the Matlab (MathWorks Inc.) *randperm* function, while 1 pseudo-curatorial text was generated using GPT-3 fine-tuned using original curatorial texts. The pseudo-curatorial text was proofread by an artist. Finally, titles were randomly assigned to pseudo-artistic images using the Matlab (MathWorks Inc.) *randi* function. The display order of pseudo-artistic images in the gallery was also drawn using the Matlab (MathWorks Inc.) *randi* function. Each set of images had its own dedicated exhibition open to the public. Both exhibitions ran for the same duration and were held at the same venue. The exhibition of the pseudo-artistic images opened directly after the exhibition of the artist Lidia Kot [11] had ended. In the laboratory, the raw images that were displayed were represented in the JPEG format, with 8 bits for each RGB channel.

#### 2.3.3 Image preprocessing for topological analysis.

Our topological analyses target structural properties such as maximal persistence, cycle density, and perimeter length. Analysing each colour channel separately would often yield redundant spatial information—cycles typically appear in the same location across channels or not at all. Consequently, a greyscale representation effectively captures these topological features while reducing computational complexity and facilitating result interpretation. This approach aligns with prior studies in computational image analysis, including [15], which examined artworks using entropy and complexity measures in greyscale.

For completeness, we demonstrate this in separate *RGB* channel analyses for 4 images (two from each group and with filtrations in both directions) in S16, S17, S18, S19, S20, S21, S22, and S23 Figs. For a detailed explanation, consult the Limitations part of the Discussion Section.

To maintain methodological consistency and comparability with established literature, we adopted greyscale conversion for all images. All images were converted to greyscale, where every pixel in greyscale was computed as a weighted average of three colours according to the formula [16,17]: *Y* = 0.299*R* + 0.587*G* + 0.114*B*, where R, G, and B are the pixel intensities in red, green, and blue. This conversion follows the CCIR 601 format originally issued in 1987 by the International Radio Consultative Committee (now the ITU Radiocommunication Sector). All images were displayed on a 21-inch computer screen with a 1920 x 1080 dpi resolution.

### 2.4 Experimental procedure

#### 2.4.1 Participants.

Participants were either second-year art students or persons of similar age and socio-economic status. The recruitment strategy was designed to control participants’ level of expertise, selecting individuals who were interested in art, familiar with gallery environments, and motivated to engage in artistic exploration. Importantly, the study targeted “young experts”—individuals with an intrinsic curiosity and openness to art — rather than professionals or trained experts, whose responses might be shaped by formal knowledge and contextual frameworks. A total of 58 participants took part in the study (16 males, 27.59%; 42 females, 72.41%), with a mean age of 22.29 years (SD = 2.25 years; age range: 19–27 years). Participants provided informed consent to take part in the study, which was approved by the Research Ethics Committee at the Nicolaus Copernicus University in Toruń (decision # 16/2021/FT).

Participants were randomly allocated to two groups of equal size, with matching procedures ensuring equivalent distributions of sex and age across the groups. Each participant in the first group was required to visit Lidia Kot’s [11] exhibition and then immediately proceed to the laboratory. Participants in the second group followed the same procedure but were instructed to visit the second exhibition, consisting of pseudo-art images instead. The participants were required to revisit the exhibition and the laboratory a second time, not less than one week after their initial visit.

#### 2.4.2 In the gallery.

Each participant attended an exhibition featuring either artistic or pseudo-artistic images at the renowned Wozownia Art Gallery in Toruń. The gallery hall in which the artistic exhibition was held was darkened and devoid of natural daylight, maintaining a low level of ambient illumination. The primary light sources were spotlights directed toward individual artworks, consistent with the artist’s original design. Identical lighting conditions were replicated for the exhibition of pseudo-artistic images.

Both exhibitions were presented in the same gallery space and were announced and open to the public. Participants were equipped with a wearable eye-tracker built into glasses and were instructed to first follow a recommended viewing order before engaging freely with the exhibition.

To measure aesthetic experience, participants completed a six-item questionnaire inspired by the Aesthetic Experience Questionnaire (AEQ, [18]) at the end of their visit. Each question was scored using an unlabelled slider (with no ticks). The slider moved over a 5-hidden-point scale, which recorded the participants’ responses.

#### 2.4.3 In the laboratory.

To ensure comparable illumination between the gallery and the laboratory settings, the laboratory sessions were conducted in a dark room with dim ambient lighting, where the only significant light sources were the computer screens displaying digital reproductions of the artistic and pseudo-artistic images.

Participants were instructed to observe these images whilst their eye movements and EEG activity were recorded. The images were presented in the following scheme: first, the title of each image appeared for two seconds, followed by the corresponding image itself for seven seconds. The choice of a seven-second viewing duration was informed by the free viewing study by [19], which showed that after an initial three-second period, characterised by increasing average fixation duration and decreasing saccade duration, both types of eye movements tend to stabilise. Each image presentation was followed by a 2-second mask of white noise and a 2-second grey mask, before the next title and image appeared.

After completing this eye-tracking and EEG session, participants scored each image on a separate computer station located in the same room using the same aesthetic experience questionnaire used in the gallery condition (included in S2 Appendix).

#### 2.4.4 Eye tracking recording and data analysis.

To capture differences in perception of the two sets of images in the laboratory, we used two basic parameters of eye movements: average fixation durations and average saccade amplitudes. Average fixation duration is a measure of attention. Short fixations indicate implicit processing, whereas longer fixations (typically above 70 ms of duration) mark the initiation of perceptual information reception and processing by the brain [20,21]. The amplitude of saccades is a measure of visual space exploration. The data were preprocessed using a positional algorithm for the automated correction of vertical drift in eye-tracking data [22].

For laboratory eye-tracking measurements, the Eyetracker SMI RED 500 was used. This device operates at a sampling frequency of 500 Hz and employs a 5-point calibration procedure for enhanced precision and accuracy. The lighting in the room was kept constant for all participants, and the luminance of the object was the same for everyone.

In the gallery, participants wore eye-trackers built into glasses. To measure attention, we used the average visual intake duration, equivalent to the average fixation duration in the laboratory setting, and the average saccade duration, equivalent to the average saccade amplitude. Both measures were computed for the areas of images extracted from the movie recorded during the visit and for the time spent by participants individually at each image. In the gallery, we used SMI Eye Tracking Glasses 2 Wireless (SMI ETG 2w), dark pupil eye tracking with a binocular sampling rate of 60 Hz, parallax compensation, and accuracy of 0.5°(declared by the manufacturer). After the glasses had been fitted to the study participant and secured, a 1-point calibration procedure was performed. During the study, scene view was recorded with a scene camera (video resolution: 1280x960p and 24FPS), and audio was recorded with an integrated microphone.

All data were curated for outlier treatments using the winsorizing method introduced by Tukey and McLaughlin [23]. That is, any value of a variable above or below 2 standard deviations on each side of the variable’s mean was replaced with the value at plus or minus 2 standard deviations itself and tested for normality using the Shapiro test. Since both laboratory and gallery eye-tracking data failed to conform to the normal distribution (Shapiro test *p* < 0.001), the comparison was performed using a non-parametric Kruskal-Wallis ANOVA and the post-hoc Dunn test with False Discovery Rate (FDR) correction for multiple comparisons. For other non-parametric comparisons, the Mann-Whitney test was used. The significance of differences in statistical analyses is indicated by the *p*-value resulting from the applied tests. Differences are assumed to be significant when the *p*-value is less than 0.05.

#### 2.4.5 EEG recording and data analysis.

Although the primary focus of the present study is the relationship between topological image properties and human perceptual experience, we also aimed to examine whether exposure to artistic and pseudo-artistic images would be reflected in differences in brain activity. To address this question, we analysed brain connectivity and graph theoretic measures derived from EEG signals. Brain connectivity provides an estimate of how different regions of the brain interact during cognitive processing and how these interactions may vary in response to specific experimental conditions. We hypothesized that the two sets of images would elicit different levels of cognitive challenge and engagement, leading to variations in the organization of brain connectivity patterns. To infer EEG-based connectivity, we assessed the statistical dependencies between signals recorded from different brain regions, thereby estimating patterns of interregional communication. Specifically, we computed the weighted Phase Lag Index (wPLI) [24,25] for all pairs of electrodes. Further methodological details of the EEG analysis are presented in S1 Appendix and the results are shown in S1 and S2 Figs.

### 2.5 Subjective experience data collection and analysis

Before the main experiment, all participants underwent an initial assessment to control for potential baseline differences between groups. This assessment included a series of psychometric measures: the full version of the Aesthetic Experience Questionnaire (AEQ; [18]), a brief survey to confirm participants’ interest in art. All questionnaires were presented in Polish (see S2 Appendix). The AEQ was administered using an in-house Polish translation, which preceded the formal publication of the official polish adaptation by Świątek et al. (2023) [26]. In addition, to evaluate individual differences in personality traits, the NEO-PI–R inventory [27] (Polish adaptation by [28]) was administered. This session also served to familiarise participants with the overall study procedure and laboratory environment. Throughout all stages of the session, an experimenter’s assistant was present to provide guidance and support.

To measure the intensity of the multidimensional aesthetic experience during the main experiment, a shortened and modified version of the Aesthetic Experience Questionnaire (AEQ–S) was used [18].

The AEQ–S comprised six items corresponding to key dimensions from [29] and the full AEQ: emotional, cultural, perceptual, cognitive understanding, proximal flow, and experiential flow. In the gallery context, participants completed a paper-and-pencil version evaluating the entire exhibition (AEQ–S–Gallery), while in the laboratory, a computer-based version (AEQ–S–Lab) was used to assess responses to individual images.

For the AEQ–S–Lab, participants rated their level of agreement with each item using a five-point slider scale (0%, 25%, 50%, 75%, and 100%). Item presentation was randomised to control for order effects. The internal consistency of the AEQ–S–Lab was high (Cronbach’s α=0.90).

### 2.6 Topological analysis

We provide a quick, informal introduction to topological data analysis in the context that we use in this paper, namely, persistent homology from filtrations of cubical complexes derived from 2D images. For mathematical definitions see S3 Appendix; for a more comprehensive account, consult [30–33].

#### 2.6.1 Persistent homology method.

Central to the analysis of topological features in datasets, regardless of their representation (for example, as an image), are the concepts of a filtration and persistence. To illustrate the construction of a 1-parameter filtration for our application, consider an image composed of pixels with varying shades of grey. A filter that permits only shades above a certain intensity to pass through might inadvertently exclude certain geometric structures formed by darker pixels. By varying the filter’s threshold, some structures may either emerge or vanish. In our example, this visual filtering results in a mathematical filtration – whereby we obtain a sequence of cubical complexes (see Subsection 4.6.2 below) ordered by inclusion, that is, a complex obtained from thresholding at a lower level of the filtration is included in the complex obtained at any higher level of the filtration. The span of filtration parameter values over which a particular topological structure exists is termed its persistence. The lowest parameter value for which a structure exists is called its birth, and the highest, its death. Persistence, therefore, is a structure’s lifetime. Structures characterised by extended persistence periods are deemed the most salient, typically offering distinctive insights into the object under examination. We have just described informally a tool used in computational topology [34], persistent homology [10].

**Fig 2:**
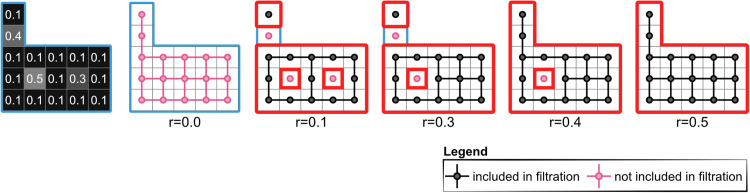
Exploring topology and persistence through filtration. Visualisation of a pixel-based structure with varying brightness levels, demonstrating the use of grey intensity (on a scale from 0 to 1) as a filtration parameter. At a grey intensity of 0.1, the structure is divided into two distinct parts, outlined by a red contour) with two ‘holes’ of grey intensities 0.3 and 0.5. This results in Betti numbers $\beta_{0}=2$ and $\beta_{1}=2$. As the filter transparency (greyness) increases, changes in the visibility of pixels at grey level 0.3, the two distinct shapes are still present, although one of the holes in the bigger part (with intensity r = 0.3) disappears, changing the Betti numbers to $\beta_{0}=2$ and $\beta_{1}=1$. At the intensity r = 0.4, the two shapes merged into one part with one hole ($\beta_{0}=1$ and $\beta_{1}=1$). If we further increase filter intensity to r = 0.5 all pixels will become visible yielding Betti numbers $\beta_{0}=1$ and $\beta_{1}=0$.

When this method is applied to two-dimensional objects such as digital images, each pixel can form vertices of elementary (e.g., in 2D, squares), and two pivotal topological characteristics come to the forefront (as further described in Section 2.6.2). The first is the number of connected components of the parts of an image that have exceeded the filtration threshold, i.e., discrete, disconnected segments within a given structure which are regions of the same or darker colour, hereinafter referred to as the ‘dimension 0’ or ‘dim 0’ cycles (formally, cycle representatives of homology classes). The second is the number of ‘holes’ present in a designated area, for example, the count of regions of a certain colour entirely encircled by areas of a different colour, hereinafter referred to as the ‘dimension 1’ or ‘dim 1’ cycles. In algebraic topology, these integers correspond to the Betti numbers (formally, ranks of homology groups), denoted $\beta_{0}$ and $\beta_{1}$ respectively, as illustrated in Fig 2.

**Fig 3:**
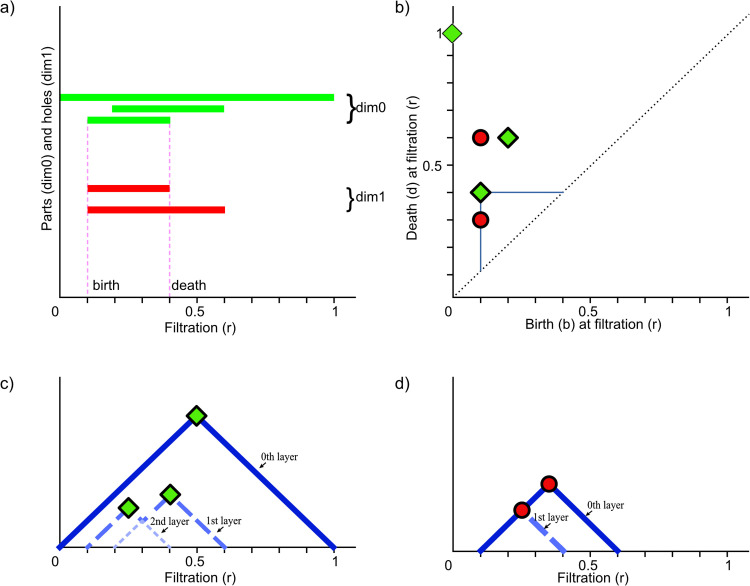
Visualisations of the filtration process. (a) ‘barcodes’ – each structure corresponds to a line segment parallel to the axis of the filtration parameter *r*, which begins when the structure appears (i.e., at point *r*_*b*_, birth) and ends when it disappears (at point *r*_*d*_, death), marked by two vertical lines for clarity; (b) persistence diagram – persistence is also representable via a two-dimensional scatter plot diagram with coordinates (*r*_*b*_, *r*_*d*_) representing births and deaths of the shape’s parts and holes on two orthogonal filtration axes marked by the horizontal blue lines orthogonal to respective axes. Naturally, points on this diagram occupy only the area above the main diagonal, (c) and (d) persistence landscape – connecting each of the birth/death points to the diagonal with vertical and horizontal lines, as shown for the uppermost point, green point on b, we get a system of ‘pyramids’ or isosceles right triangles. After rotating the persistence diagram by π/4, it becomes a persistence landscape drawn separately for dim 0 – separate connected components (Fig 2c) and dim 1 – holes (Fig 2d). Persistence landscapes, in general, consist of several layers, where the *n*-th layer is the *n*-th highest value at a given point *r* of filtration. In **(c)**, the top (0-th) layer is constructed from the ‘pyramid’ built on the largest barcode that lasts throughout the filtration (this pyramid is greater than all others at any value of *r*), the 1st and 2nd layers are constructed from the pyramids built on 2 shorter barcodes – whose corresponding ‘pyramids’ intersect, resulting in 3 layers (a full definition of layers is provided in S3 Appendix).

Through persistent homology, it is possible to capture the emergence (birth) and disappearance (death) of components and voids relative to filtration values. The evolution of those topological properties is commonly presented in the form of barcodes [34]. The barcodes are often transformed into persistence diagrams [35] and persistence landscapes [36]. These techniques are elucidated and depicted in Fig 3, offering graphical insights that effectively highlight variations in topological properties among the objects under scrutiny.

As well as providing valuable graphical representations for qualitative assessment, persistence landscapes (sequences of piecewise linear functions) also enable us to use standard tools from statistics [36]. Without a transformation to persistence landscapes, a multiset of barcodes is inadequate for defining even simple notions such as mean and median. In addition to comparing landscapes using L1 distance (see S10 Fig), we also used simple derived measures such as the total area beneath the ‘pyramids’ in the persistence landscape (this can also be considered the L1-norm of the persistence landscape). Even though this quick quantification entails some information loss concerning each image’s topological structure, it provided an adequate first glimpse into distinguishing between the two sets of images in our application.

However, our goal was not just to investigate differences in art pieces, but also to explore whether human perception was guided by the topological properties of the images uncovered via persistent homology. For this purpose, we derived measures that could bind these topological structures with their spatial distribution, namely, feature maps. We then investigated their relationship with the gaze-derived heatmaps, as explained in the following sections.

#### 2.6.2 Cubical complexes.

An important aspect of the topological investigation is the decision on how to decompose the object of study, i.e., the image, using topological structures. Here, we shall compute persistent homology from a filtration of cubical complexes [31,32] with the *V*-construction [37].

**Fig 4:**
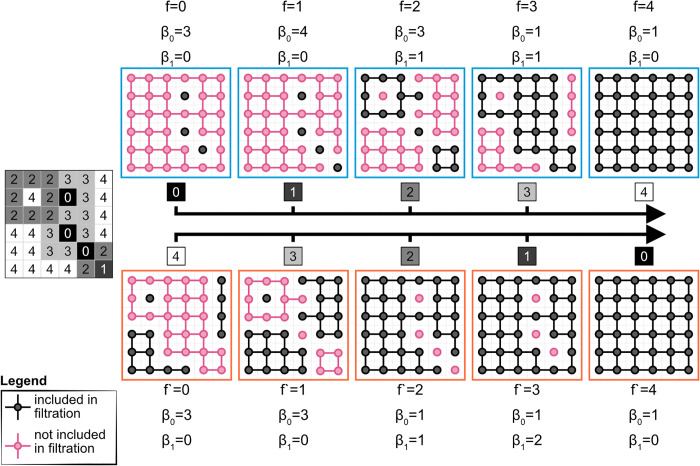
Demonstration of the duality of black-to-white (BW) (first row) and white-to-black filtrations (WB) (second row). The image from which the cubical complexes are derived is to the left of the rows in the middle. Components (and corresponding connections) that comprise a cubical complex at a particular step of the filtration, *f* (or $f^{\prime }$), are coloured in black, whereas the pink components are not included in the filtration. Pink components also belong to the dual cubical complex coming from the reverse filtration at step $f^{\prime }=M-1-f$, where *M* + 1 is the total number of filtration steps in [0,*M*].

To obtain a cubical complex, we exploit the fact that the digital representation of an image is a matrix, where the pixels are laid out in a grid, where we take the neighbourhood of a pixel to be limited to the 4 compass directions (some of which are not possible, for example, the pixels at the periphery of the image). Using this grid structure, a cubical complex is formed as a topological space constructed from a union of vertices and edges, where a vertex is assigned to a pixel, and edges are formed from adjacent pixels which satisfy a condition, for example being above (or below) a certain value in the greyness scale as is demonstrated in Figs 2 and 4. Each cubical complex thus created (one for each value of the filtration) is dependent on the relationship between neighbouring pixels. Hence, a cubical complex could be, for example, a set of disjoint pixels located in the image matrix, pixels covering some extent of an image, pixels encircling an area containing pixels that are not yet included in the filtration (thus forming a hole), or any combination of the above.

From this, it naturally follows that the topological structures we can investigate are limited to 0-dimensional (connected components) and 1-dimensional (loops) cycles. We record the locations in a filtration where such structures emerge (births) and disappear (deaths). The list of all such births and deaths results in the multi-set of barcodes to which we can associate a persistence landscape (see Fig 3). More details on obtaining persistence landscapes from cubical complexes derived from image data can be found in S3 Appendix. Finally, we also plot Betti curves $\beta_{d}(f)$, functions defined in (S3 Appendix) for each dimension *d*, which show how the Betti numbers (the number of barcodes present at any given point of the filtration) vary throughout the filtration.

Apart from the natural and easily definable transition from image to a nested sequence of cubical complexes, an advantage of this cubical representation of the image is that once the topological content of the image is established, it is possible to study not only the properties of the cycles but also their spatial distribution and spatial extent within an image. This allowed us to compare the images from the exhibition in two ways – by following a standard approach of analysing persistence properties using persistence landscapes and, as introduced in our work, by exploring the spatial distribution of the topological structures using feature maps.

#### 2.6.3 Duality of persistent homology of cubical complexes on images.

Lastly, the persistent homology groups of cubical complexes thus defined exhibit a simple but interesting duality. Topological invariants computed for the filtration starting from black and finishing on white (BW) can be related to invariants computed from the reverse filtration starting from white and finishing on black (WB), namely, persistent homology classes of dimension 1 can be (injectively) mapped to dimension 0 classes (connected components) of the reverse filtration. This is similar to results obtained in [37,38] and is an example of Alexander duality [39]. A proof can be easily deduced from looking at Fig 4; a sketch proof is provided in S3 Appendix.

The advantage of this is that by using information from both filtrations (BW and WB) and only dimension 1 cycles, we capture almost all information about cycles in images – this was utilised in the analysis of feature maps. The only discrepancies are due to cycles which comprise or interact with cycles at the boundary of the image.

Fig 4 illustrates the duality of filtrations via a simple example. At each filtration step (different steps of the dual filtrations), the underlying grid is partitioned by the connected components (0-cycles of one cubical complex and its complement with respect to the grid, which we shall denote the ‘dual’ complex). Let *f* and $f^{\prime }$ be the steps in the BW and WB filtrations, respectively, where $f^{\prime }=M-1-f$. For this toy example, the total length of a filtration, *M*, is 4 (for a general greyscale image, *M* = 255). Hence, the black components for *f* = 0 are pink components for $f^{\prime }=3$ and vice versa. Hence, as shown in Table 1, for any given pair $\left(f,f^{\prime })=(f,4-1-f\right)$, the connected components from both filtrations partition the underlying grid; hence, the number of partitions of the grid is also the sum of the zeroth Betti numbers.

**Table 1:** Number of partitions for filtration steps for the toy example.

*f*	$f^{\prime }$	#partitions	$\beta_{0}^{f}$	$\beta_{0}^{f^{\prime }}$
0	3	4	3	1
1	2	5	4	1
2	1	6	3	3
3	0	4	1	3

For the BW filtration at a grey intensity level, *f* = 0 (on a scale from 0 to 4), the cubical complex consists of three distinct parts marked with black dots. This results in the Betti numbers being $\beta_{0}=3$ for the three components and $\beta_{1}=0$, indicating the lack of holes. As the filter transparency increases to 1 (*f* = 1), one more disconnected component is added, increasing the zeroth Betti number $\beta_{0}$ to 4. In the next filtration step *f* = 2, there are 2 changes: (1) two components are joined (bottom right corner), decreasing $\beta_{0}$ by 1, and (2) another component merges with new elements joining the filtration, forming a hole, thus increasing $\beta_{1}$ to 1. At intensity *f* = 3, all 3 shapes merge into a single connected component with one hole ($\beta_{0}=1$ and $\beta_{1}=1$). If we further increase filter intensity to *f* = 4, all pixels will be added to the cubical complex, yielding Betti numbers $\beta_{0}=1$ and $\beta_{1}=0$.

Now, for every filtration step *f* with non-zero $\beta_{1}$, we can find the dual step, $f^{\prime }=M-1-f$, in the reverse filtration, where there exists a dimension 0 component, which we call its dual. For example, as shown in Fig 4 for *f* = {2, 3} top row, a cycle exists in the top left corner, and in WB filtration for $f^{\prime }=\{1,0\}$, bottom row, there is a connected component present in that corner. If we study the reverse filtration, the same rule applies – for the two steps, $f^{\prime }=\{2,3\}$ in which $\beta_{1}$ is not zero, we can find dual components in steps *f* = {1,0}. In all cases, the persistence of dual components is equal (unless there is an interaction with a cycle on the periphery). This can also be seen within the barcodes and persistent landscape, as demonstrated with another example where most cycles do not interact with the periphery of the image in S9 Fig.

Altogether, with this duality, we have shown that the topological properties derived from a single filtration in both dimensions are similar to those from both filtrations but in the same dimension, and we exploit this fact in constructing feature maps.

Violations of Alexander duality

Finally, we also investigated to what extent Alexander duality was violated by each image, and whether there were differences between the art and the pseudo-art. We hypothesise that artists would be very aware of the existence of the rectangular frame around any image they create, which encloses their composition. Artists will be deliberate about which shapes touch the frame, which will affect the distribution of cycles touching the frame and thereby impacting Alexander duality.

We investigate this by computing the difference in the areas under the Betti curves (or integrated Betti numbers) for the opposing filtrations and dimensions and normalizing by dividing by the mean area under those two Betti curves (denoted $\beta_{dim0}^{BW}(f)$ and $\beta_{dim1}^{WB}(f)$, where *f* is the filtration parameter). Hence the area under the Betti curve for dimension 0 cycles in the black to white filtration (BW), ${\bar{\beta }}_{dim0}^{BW}$ is given by the integral, ${\bar{\beta }}_{dim0}^{BW}:=\int \beta_{dim0}^{BW}(f)df$. Since ${\bar{\beta }}_{dim0}^{BW}>{\bar{\beta }}_{dim1}^{WB}$ always, we used the following positive measure for violations of Alexander duality:


$$ADV_{BW}:=\frac{\left({\bar{\beta }}_{dim0}^{BW}-{\bar{\beta }}_{dim1}^{WB}\right)}{Mean\left({\bar{\beta }}_{dim0}^{BW},{\bar{\beta }}_{dim1}^{WB}\right)},$$ (1)

and also its counterpart with BW and WB interchanged, *ADV*_*WB*_. This measure is bounded between 0 (no violation) and a maximum of 2.

#### 2.6.4 Topological feature maps.

In visual art, artistic impressions are created with objects, colours, and textures. Whereas the first two are directly impacted throughout the artistic process, texture is controlled at the earliest stages, when the technique is chosen. Furthermore, texture has a dual nature – for images, the texture is the spatial distribution of colours or changes in contrast on the canvas, whereas, for physical objects, texture refers to the smoothness or roughness of an object’s surface. In painting, texture is a combination of these two aspects, as both are combined (by the artist’s selection of canvas and paint) to create the overall artistic impression.

In this space of objects, colours, and textures, our method is inherently sensitive not only to shapes of various sizes but also to their spatial distribution, which makes it suitable for investigating both objects and textures. The motivation is that, in areas with rich texture, there are many small patches of colour, which translate into a large number of cycles per unit area. On the other hand, smooth areas do not vary as abruptly in terms of colour, so they shall contain fewer distinct cycles per unit area (low cycle density).

**Fig 5:**
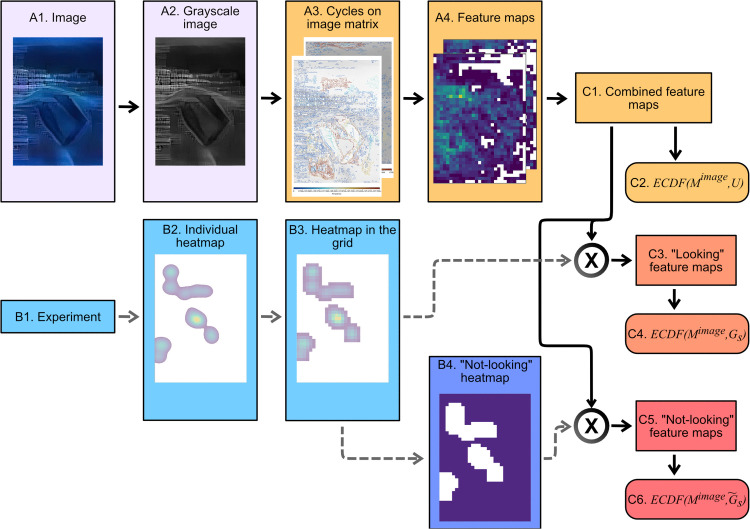
Visualisation of the pipeline for obtaining feature distributions and gaze maps. After the image is converted to greyscale, the spatial distribution of cycles is obtained from the BW and WB filtrations, resulting in feature maps for each type of topological feature (for example, cycle density, persistence or cycle perimeter). Firstly, for each type of topological feature (for example, cycle density, persistence or cycle perimeter) and each image, the Empirical Cumulative Distribution Function (ECDFs), $ECDF(M^{image},U)$, is obtained, which is intrinsic to the image. Secondly for a subject *s*, gaze-heatmaps *G*_*s*_ and its complement heatmap ${\tilde{G}}_{s}$ are used to obtain $ECDF(M^{image},G_{s})$ (looking) and $ECDF(M^{image},{\tilde{G}}_{s})$ (not looking), by weighting the image feature maps with the corresponding gaze-heatmap.

To explore textures and differences in the spatial extent of visual structures between the two sets of images and their effect on perception, we introduce topological feature maps. The pipeline visualising this process is shown in Fig 5. Topological feature maps are topographic maps conveying the spatial location of properties of various topological features of an image that were obtained by persistent homology calculations.

**Fig 6:**
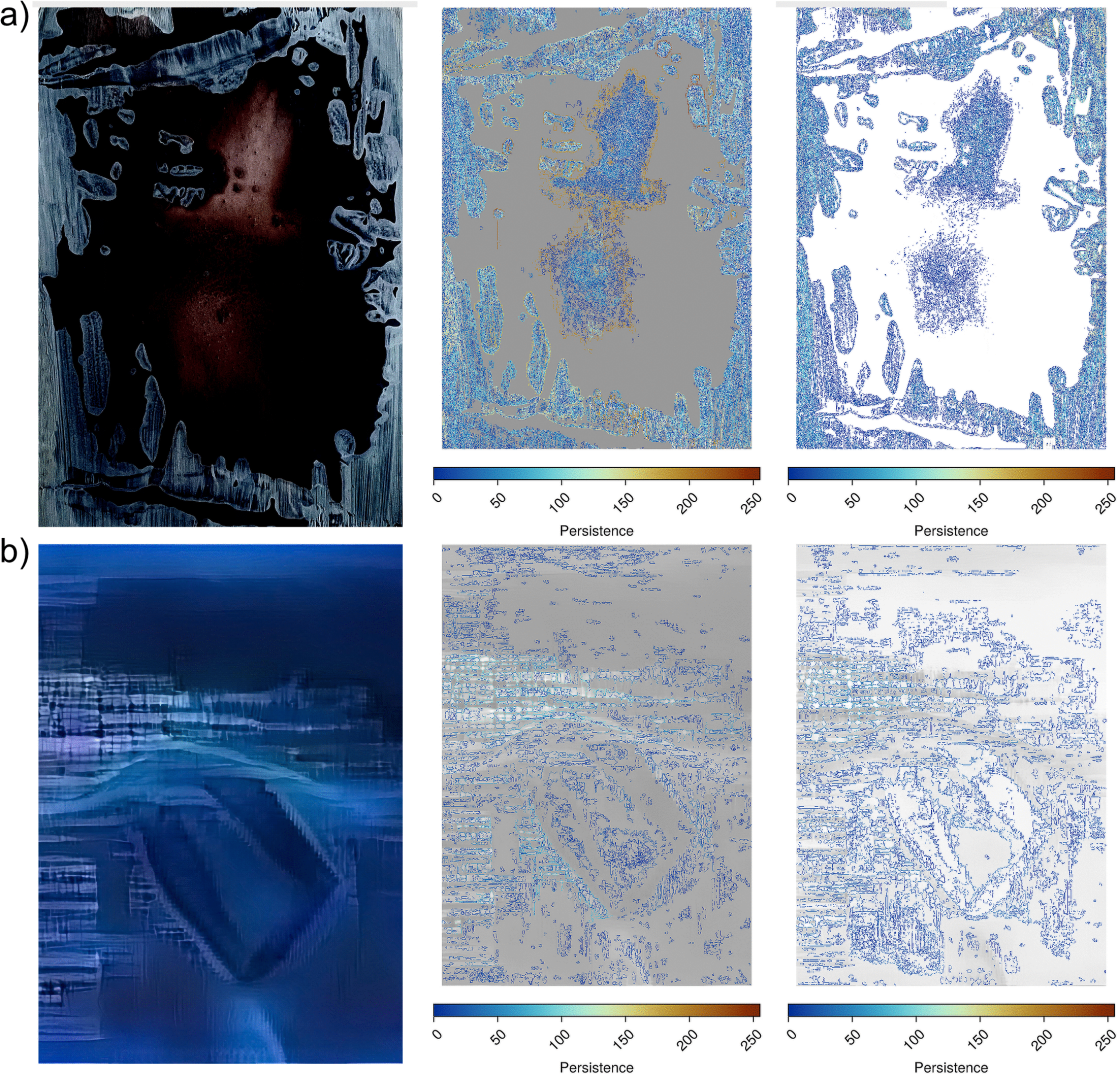
Visualisation of cycles for BW filtration (middle column) and WB filtration (right column) for an artistic [11] (a) and pseudo-artistic image (b). Cycles presented in the middle and right-most columns are coloured according to their persistence, as indicated by each legend.

Starting with the image depicting the location of all 1-dimensional cycle representatives (Step *A3* in Fig 5, samples shown in Fig 6), one can overlay a square grid mesh consisting of non-overlapping windows of fixed size. Then, for each window, a feature is derived (*A4* in Fig 5) – it could be cycle density (computed as the total number of cycles in each grid window), maximal 1-dimensional cycle perimeter (the maximal number of pixels belonging to the perimeter of any cycle lying within the grid window), or maximal persistence (given by the largest persistence of all cycles found in the grid window). A feature map can therefore be specified by a matrix *M*^*image*^ where, depending on the choice of topological descriptor, $M_{i,j}^{image}$ will be the feature map value in the square in the *i*th row and the *j*th column of the grid over that image. Full details can be found in the S4 Appendix.

These three topologically derived measures, cycle density, maximal persistence, and cycle perimeter, are proxies for texture in the image, local contrast, and shape size, respectively.

Feature maps are derived from the locations of 1-dimensional cycle representatives, and this, at first glance, excludes the information about the topology in dimension 0. To overcome this, we exploit the duality feature of cycles and incorporate the information about the cycles in dimension 0 from BW filtration by studying their dual in dimension 1 in WB filtration. As demonstrated in S9 Fig and also shown in S10 Fig, very little is lost by only using dimension 1 cycles.

Finally, we devised composite feature maps derived from both filtrations (WB and BW, step *C1* in Fig 5) and used these composite feature maps in our study. The feature maps were as follows: (1) cycle density, which was obtained from summing the densities per unit grid area from both filtrations; (2) maximum cycle persistence, which took the maximum persistence of all the cycles from both filtrations present per unit grid area; (3) maximum cycle perimeter, which took the maximum perimeter of all the cycles from both filtrations present in any particular square in the grid.

All values from a feature map *M*^*image*^ yield a distribution that was specified using its Empirical Cumulative Distribution Function ($ECDF\left(M^{image},U\right)$, where *U* is a uniform weight, thus not favouring any regions, *C2* in Fig 5). This function is the cumulative sum of the count of windows having a given property value (for example, a cumulative sum of a histogram showing the counts of windows having a given cycle density). Even though some 2D spatial information is lost, we shall show that the ECDF provides a convenient way of comparing and visualising distributions derived from the feature maps of the images combined with the gaze maps of individuals, as will be seen below.

Note that all images were the same size, and the same grid mesh size (50 pixels by 50 pixels) was used for each image. Although grid size may seem arbitrary, we verified that the choice makes very little difference to the structure of the ECDFs by testing multiple versions of the grid size (see S30 Fig) and Section 3.8 for a more detailed discussion).

#### 2.6.5 Computation of cycles and software.

Cycle representatives for dimension 1 homology classes used in this study were obtained using the ‘Ripserer.jl’ Julia library [40–43] and they are shown in S12, S13, S14, and S15 Figs for art, pseudo-art, and using both filtrations.

Further details, together with the algorithmic framework for this procedure, are presented in S4 Appendix. While the ECDF curves characterize the underlying nature of the images’ textures, gaze-weighted ECDF curves, introduced in the next section, will provide insight into which features the participants were interested in while interacting with art (or pseudo-art).

#### 2.6.6 Feature maps’ robustness to image distortions.

Our exploration of topologically derived properties is grounded in their inherent independence from arbitrarily defined coordinates and metric attributes of perceived objects [44]. It allows for direct comparisons between different images by examining their intrinsic features. In the context of 2D visual art, persistent homology was used here to analyse images based on either a single greyscale channel or three colour channels. The use of a common scale of 256 points for pixel intensity ensures a fair and unbiased comparison across all analysed images. Topological analysis using pixel-based cubical complexes is relatively fault-tolerant to differences in image resolution. Our analyses show that the output of topologically derived features is only affected after a large reduction in the resolution of analysed images (for details, see S11 Fig).

Furthermore, topological properties exhibit a notable resilience to disturbances, such as variations in visual acuity or noise [45], including changes in illumination [32], as well as image degradation [46]. Moreover, since these features reflect the distribution of local contrast rather than its absolute global value, they are expected to remain stable under global contrast variations. To confirm that the outcomes of our topological analyses are not artifacts of global contrast differences, we conducted persistent homology computations on images with systematically altered contrast levels (consult S5 Appendix for full details). The resulting topological descriptors remained largely invariant across a wide range of contrast manipulations (see S24, S25, S26, S27, S28, and S29 Figs), supporting the contrast insensitivity of the method. The numbers of barcodes are largely preserved, although these can shrink or be stretched.

Importantly, the relation between image sets is preserved – this is shown in S28 and S29 Figs, where we demonstrate the *L*1 distance computed between all pairs of images, after the transformation was applied to all images independently. In all cases, the distance between pseudo-art images was lower than for art images.

#### 2.6.7 Analysis of the relationship between feature maps and fixation patterns.

Potential differences in the eye movements of the investigated groups could have resulted from the different topological properties of the observed image sets. To verify this hypothesis, we compared information from eye-tracking movements and topologically derived properties of the images.

To this end, fixation sequences lasting over 75 ms were extracted from the eye-tracking data of each participant and used to generate fixation heatmaps (*B2* in Fig 5). These heatmaps, created by applying a Gaussian kernel to qualifying fixation positions, were produced for each participant, image, viewing, and session (a total of 4 viewings, 2 per session). More details on heatmap generation can be found in S4 Appendix.

Apart from the feature distributions intrinsic to the image, $ECDF\left(M^{image},U\right)$ we studied two weighted- $ECDF\left(M^{image},G_{s}\right)$ weighted with individual heatmap *G*_*s*_ and $ECDF\left(M^{image},\tilde{G_{s}}\right)$ weighted with a uniform weight where a participant did not look $\tilde{G_{s}}$ (*C4* and *C6* in Fig 5 respectively). The main idea of weighting via gaze maps is as follows – the more time a participant spent looking at a specific window in the mesh grid, the more important that window was and the higher the weight given to it; this also means that the topological properties of that window are more important. For example, if a person only looks at the parts of an image with highly persistent cycles and ignores other parts of the image, then the distribution obtained will be highly skewed and very different from the underlying distribution of persistent cycles in the image that is obtained from the neutral or ‘uniform gaze’. An electronic scanner will have a uniform gaze – all parts of the image are given equal weight; there are no preferred areas.

ECDF comparison

Two comparisons were made between datasets to investigate what kinds of features participants’ eyes were drawn to. The first comparison was about the “ground truth” for an image and the areas where subjects look at. This was done by comparing the distributions of the mean squared ECDF errors (MSE), computed as a mean squared difference of $ECDF(M^{image},U)$ and $ECDF(M^{image},G_{s})$ (looking), that is a mean squared difference of the ECDF from feature map of an image and ECDF derived from a feature map of the same image weighted with a heatmap of subject *s* viewing it. The statistical tests were performed by comparing the distribution of MSE for artistic images with the distribution of MSE for pseudo-artistic images.

The second comparison was about the areas that subjects looked at and areas where they did not look. This was computed as mean squared difference and mean difference between $ECDF(M^{image},G_{s})$ (looking) and the ECDF derived from the complement gaze, $ECDF(M^{image},{\tilde{G}}_{s})$ (not looking), thereby comparing features that a viewer, *s*, looked at with the features in the image that the (same) viewer did not explore. The first measure was used to assess if there are group differences between the art images and the pseudo-art images, while the second was necessary for interpretation of the results.

The comparisons were performed between artistic and pseudo-artistic images. In all comparisons, data was used from all images for all visits and sessions and repeated for three feature maps – maximal cycle persistence, maximal cycle perimeter, and cycle density. More details of this procedure, including a precise definition of these feature maps, can be found in S4 Appendix.

### 2.7 Statistical analysis

The statistical methods employed for eye-tracking data analysis are detailed in the corresponding section. Presented below are the statistical approaches used for comparing persistence landscapes and conducting image-based analyses.

#### 2.7.1 Topological data analyses.

Empirical cumulative distribution function analysis

In the study of the spatial distribution of cycle density, the shapes of the ECDF curves generated from the grid of images were compared using the Kolmogorov-Smirnov (KS) statistic [47–49]. The choice of grid size was motivated by the achieved total number of windows (smaller grid size) – the more windows, the better the statistical power of the KS test. However, this was limited by the fact that the window size needed to be large enough to contain a range of cycles.

The Friedman test was used to assess the difference between regions where subjects looked at and did not look at (using combined data from two exhibitions, one test for each of the feature maps), and in the post hoc analysis, the Wilcoxon signed-rank test was used to determine differences within blocks (with one block being data for the art exhibition and another for the exhibition of pseudo-artistic images).

#### 2.7.2 Statistical Image Properties (SIP).

Many statistical measures can be applied to the analysis of images, such as artworks. As a benchmark for the persistent homology method, we have chosen statistics commonly used for artistic image analyses, representing a wide variety of parameters, from spectral to non-linear measures such as complexity or entropy. The final set of SIPs used for comparison with persistent homology analyses included: Fourier slope – the least squares fit of a line to the log-log power spectrum of the images transformed into the frequency domain [50], Self-similarity – self-similarity assessment based on the Pyramid Histogram of Oriented Gradients (PHOG) that is of occurrences of gradient orientation computed on a dense grid of uniformly spaced cells on an image to facilitate edge detection [51], Complexity – the mean norm of the Histogram of Oriented Gradients (HOG) across all orientations [52], Anisotropy – the variance of gradient strength in the HOGs across its bin entries [52] and the edge density [53]. The details of the statistical methods can be found in the cited articles. The analyses were performed using scripts provided by the authors of the relevant methods found in the Supplementary Materials to the cited articles.

The *p*-value represents the probability of obtaining the results by chance alone. In this study, a value below 0.05 was used to indicate the statistical significance of the results.

## 3 Results

### 3.1 Aesthetic experience

None of the assessments administered during the initial session revealed significant differences between participants (all p > 0.05), confirming that the groups were comparable at baseline in terms of aesthetic experience tendency, interest in art, personality traits and electrical brain activity – factors that could otherwise differentially influence responses to the presented images.

#### 3.1.1 Aesthetic experience differences in the gallery.

Aesthetic experience scores were gathered at the conclusion of each participant’s visit to the gallery, using a paper-and-pencil questionnaire. Participants evaluated their overall exhibition experience.

Analysis of the collected data with two-way mixed-design MANOVA: Exhibition showed no significant differences: *F*(6,46) = 1.748, *p* = 0.131, $\eta_{p}^{2}=0.131$.

#### 3.1.2 Aesthetic experience differences in the laboratory.

**Fig 7:**
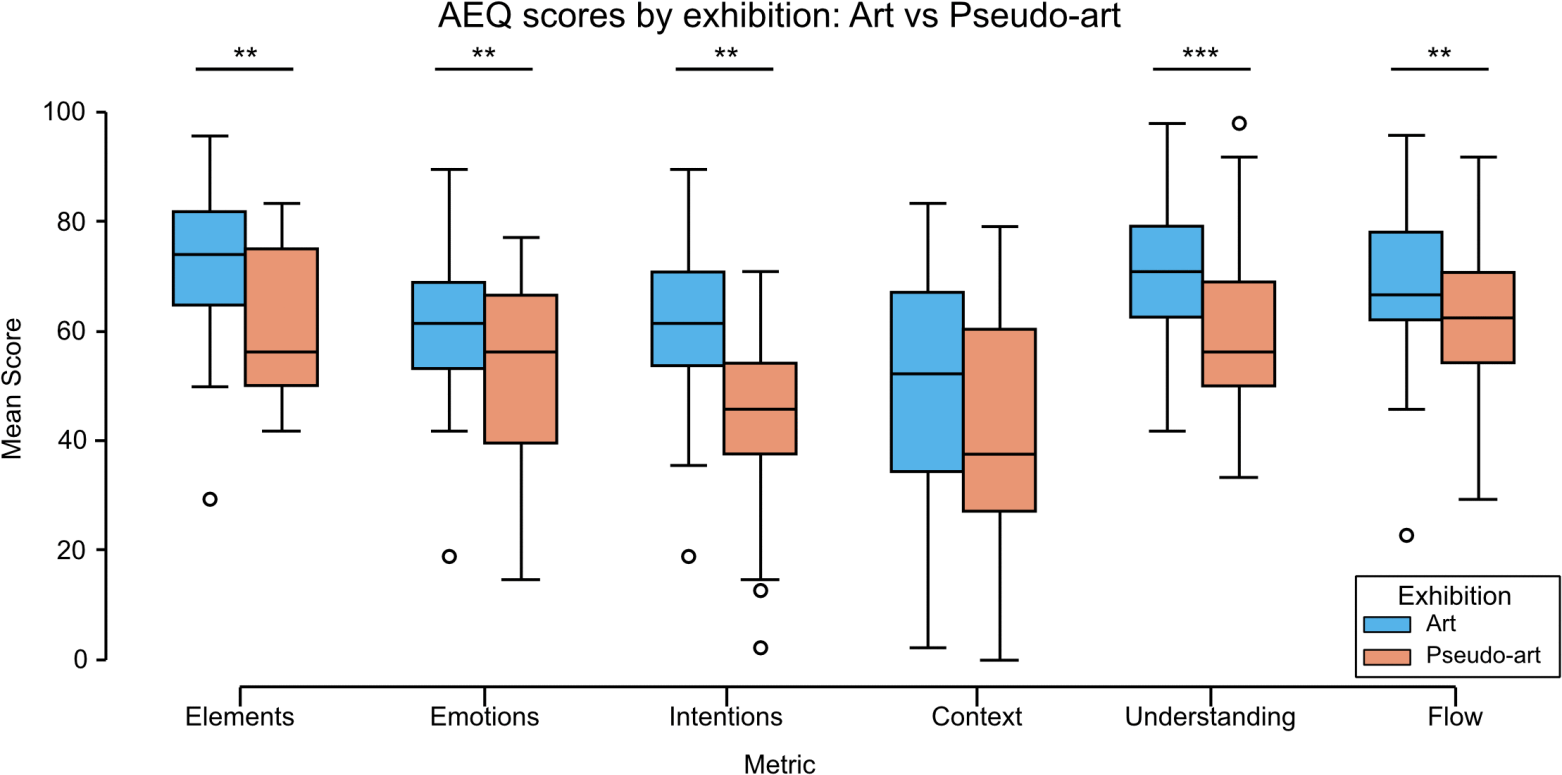
The laboratory comparison between art and pseudo-art of average aesthetic experience questionnaire scores. Responses were collected using a 5-point slider scale (0%, 25%, 50%, 75%, 100%). The boxplots show medians for a given group (horizontal line) together with the middle 50% of the data denoted by the interquartile range box (the distance between the first and third quartiles) and ranges for the bottom 25% and the top 25% of the data values, excluding outliers (whiskers). Stars above horizontal lines connecting boxplots denote significance levels: *** for *p* < 0.001; ** for *p* < 0.01; * for *p* < 0.05.

Aesthetic experience scores were collected in the laboratory for each image individually, independently of the eye-tracking. Subsequently, the scores were averaged across images for each participant. Two-way mixed-design Multivariate Analysis of Variance, MANOVA yielded a significant effect of the exhibition: Exhibition, *F*(6,49) = 4.14, *p* = 0.002, $\eta_{p}^{2}=0.336$. To further investigate the multivariate effect of exhibition type, separate one-way ANOVAs were conducted for each dependent variable. The results revealed significant effects of exhibition type on most measures of aesthetic experience: Elements perception: *F*(1, 54) = 22.57, *p* < 0.001, $\eta_{p}^{2}=0.295$; Emotional intensity: *F*(1, 54) = 12.01, *p* = 0.001, $\eta_{p}^{2}=0.182$; Understanding the artist’s intent: *F*(1, 54) = 23.48, *p* < 0.001, $\eta_{p}^{2}=0.303$; Context clarity: *F*(1, 54) = 4.02, *p* = 0.050, $\eta_{p}^{2}=0.069$; Understanding flow: *F*(1, 54) = 17.89, *p* < 0.001, $\eta_{p}^{2}=0.249$; Personal experience of flow: *F*(1, 54) = 10.18, *p* = 0.002, $\eta_{p}^{2}=0.159$ See Fig 7.

### 3.2 Results of the eye tracking and EEG analysis – differences between groups watching the two image sets

Results of the eye tracking analyses.

The results showed statistically significant differences between the groups in terms of eye movements under both laboratory conditions and during gallery visits.

**Fig 8:**
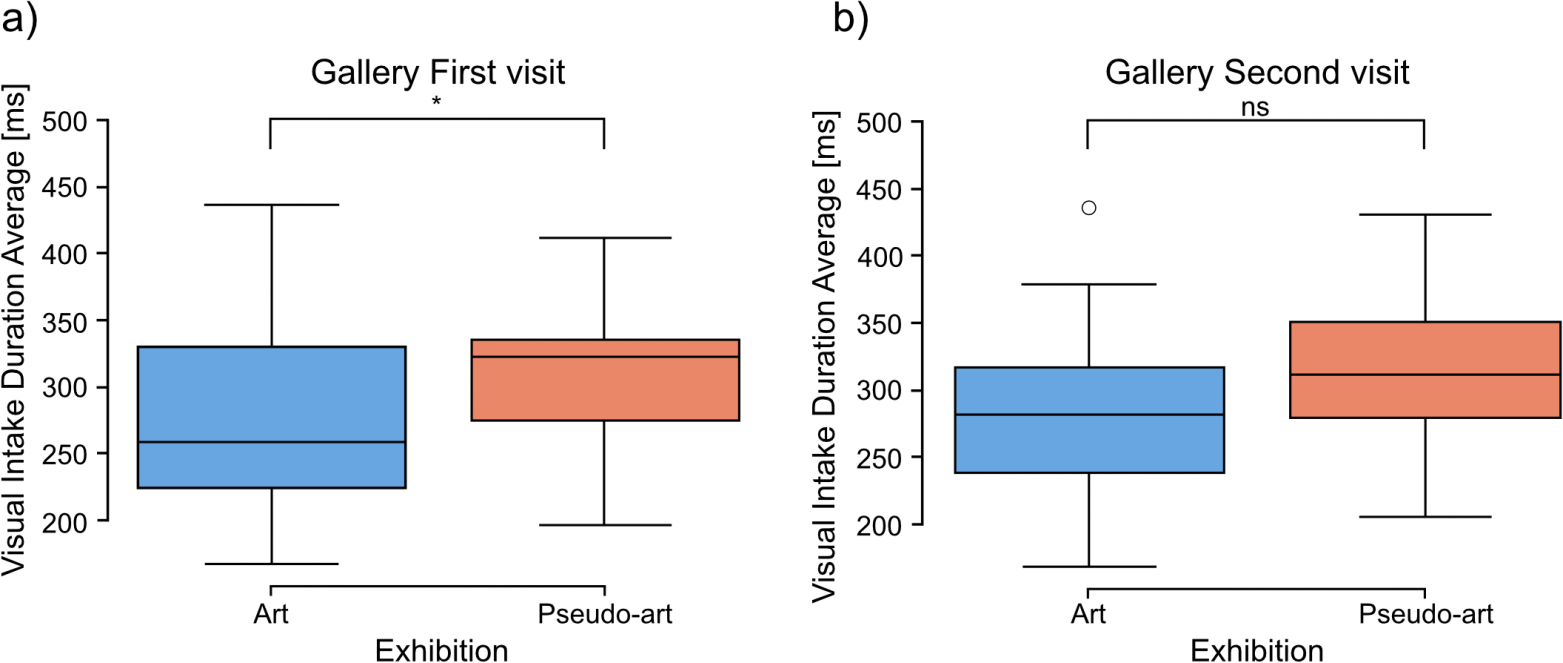
Comparison between art and pseudo-art of average visual intake duration during first (a) and second (b) visit in a gallery. The boxplots show medians for a given group (horizontal line) together with the middle 50% of the data denoted by interquartile range box (the distance between the first and third quartiles) and ranges for the bottom 25% and the top 25% of the data values, excluding outliers (whiskers). A * above a horizontal line denotes *p* < 0.05, *ns* denotes *p* > 0.05.

Analyses of data collected during gallery visits showed significantly longer average visual intakes for the pseudo-art images (*mean* = 319.79, *SD* = 54.79) than for the art ones (*mean* = 281.87, *SD* = 65.84) in the first visit (2-tailed t-test: *t* = −2.2222, *df* = 47.98, *p* = 0.031, Fig 8a).

In the second gallery visit, average visual intakes for the pseudo-art images (*mean* = 317.38, *SD* = 64.19) were also longer than for the art ones (*mean* = 282.91, *SD* = 77.71), however, the difference showed only a trend toward significance (2-tailed t-test: *t* = −1.7172, *df* = 47.98, *p* = 0.092, Fig 8b).

**Fig 9:**
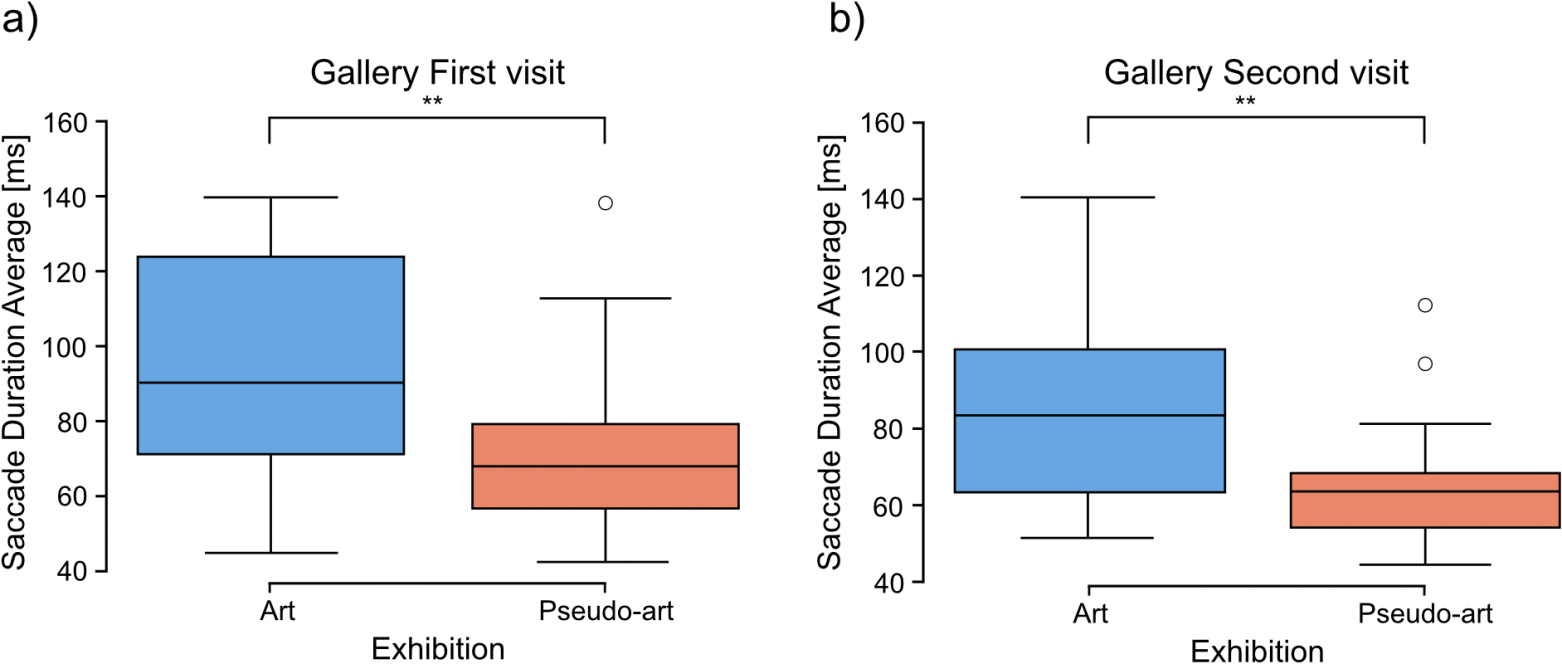
Comparison between art and pseudo-art of average saccade duration during first (a) and second (b) visit in a gallery. The boxplots show medians for a given group (horizontal line) together with the middle 50% of the data denoted by interquartile range box (the distance between the first and third quartiles) and ranges for the bottom 25% and the top 25% of the data values, excluding outliers (whiskers). A ** above the horizontal line over 1st visit boxplot denotes *p* < 0.01.

Similar analyses of the average saccade durations yielded significance via the Mann-Whitney U test in both visits (for the first: art images *Med* = 83.33, pseudo-art images *Med* = 63.61, *U* = 473, *p* = 0.001, and for the second visit art images *Med* = 90.28, pseudo-art images *Med* = 68.52, *U* = 461, *p* = 0.003). Fig 9a) shows results for the first and Fig 9b) for the second visit.

**Fig 10:**
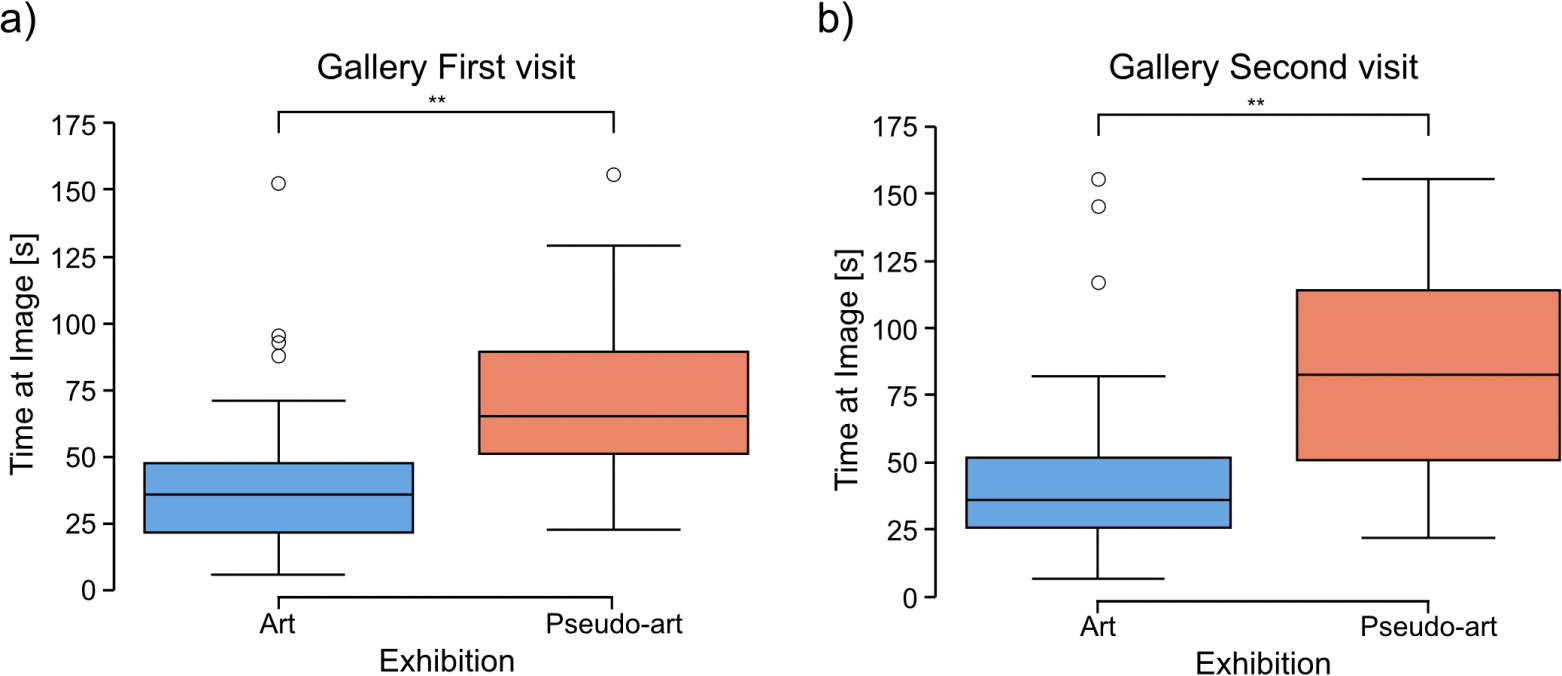
Comparison between art and pseudo-art of average time spent at image during the first (a) and second (b) visit to the gallery. The boxplots show medians for a given group (horizontal line) together with the middle 50% of the data denoted by interquartile range box (the distance between the first and third quartiles) and ranges for the bottom 25% and the top 25% of the data values, excluding outliers (whiskers). ** above horizontal line over 1st visit boxplot denotes *p* < 0.01; *** above horizontal line over 1st visit boxplot denotes *p* < 0.001.

Moreover, a comparison of the time participants spent on each image in the gallery, calculated as the sum of saccades and fixations for each image, showed that pseudo-art images attracted significantly longer viewing times than art ones during both visits (for the first: art images *Med* = 35.71, pseudo-art images *Med* = 79.39, *U* = 133, *p* = 0.0005, Fig 10a; and for second visit: art images *Med* = 36.65, pesudo-art images *Med* = 64.59, *U* = 168, *p* = 0.005 (Mann-Whitney U test), Fig 10b.

**Fig 11:**
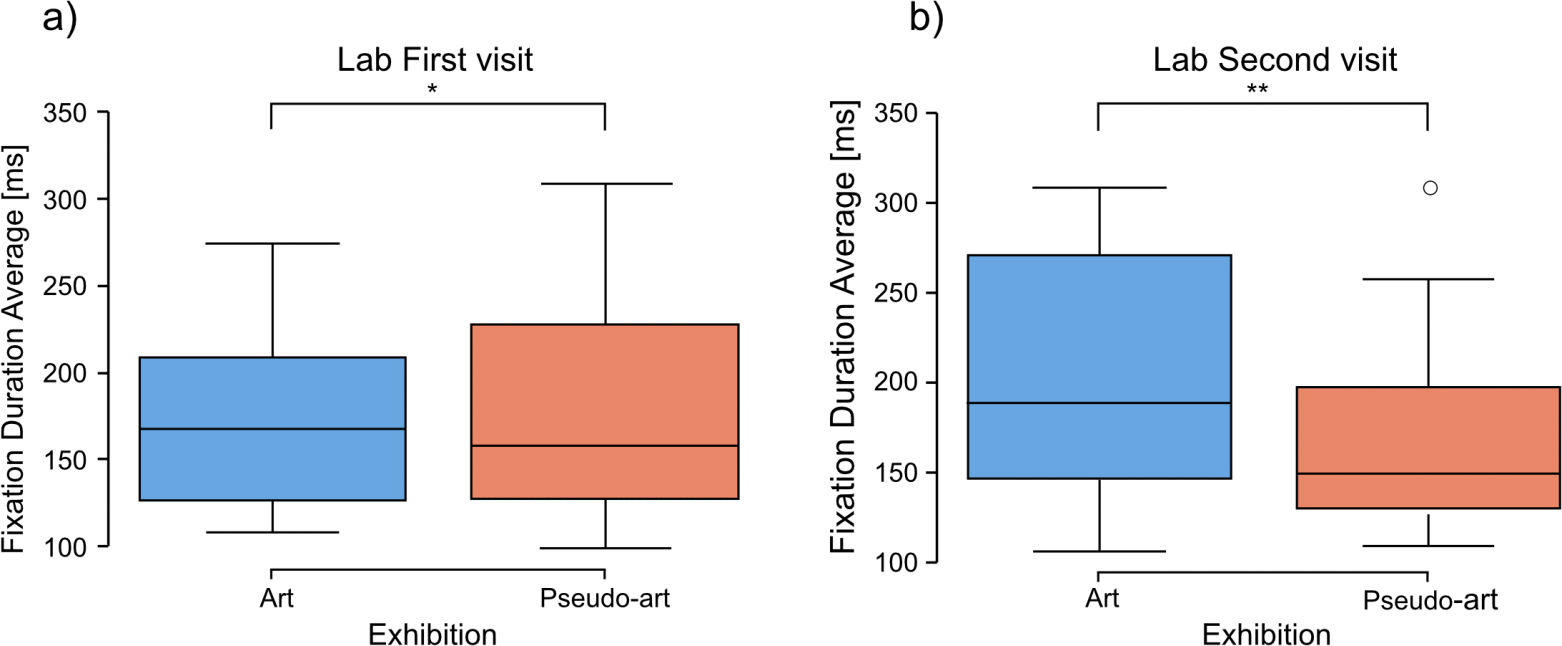
Comparison between art and pseudo-art of average fixation duration during first (a) and second (b) visit in a laboratory. The boxplots show medians for a given group (horizontal line) together with the middle 50% of the data denoted by the interquartile range box (the distance between the first and third quartiles) and ranges for the bottom 25% and the top 25% of the data values, excluding outliers (whiskers). A * above the horizontal line over the 1st visit boxplot denotes *p* < 0.05, whereas ** denotes *p* < 0.01.

Interestingly, during laboratory visits, the longer eye fixations were observed for the group viewing the artist’s works. In detail: Mann-Whitney U test results for the first visit: art images *Med* = 197.6551, pseudo-art images *Med* = 149.9637, *U* = 566.0, *p* = 0.042. Similar analyses for the second visit showed no significant results (*U* = 340.0, *p* = 0.70, Fig 11).

Laboratory comparisons of the average saccade durations between artistic and pseudo-artistic images revealed no significant differences during either visit(first visit: U test, *U* = 436.0, *p* = 0.95; second visit: *U* = 369.0, *p* = 0.91).

Both the gallery and laboratory eye-tracking results revealed significant group differences between artistic and pseudo-artistic images in terms of average visual intake and fixation durations. However, they followed different patterns. Specifically, during the first visit, participants in the laboratory exhibited longer average fixation durations when viewing artistic images, whereas in the gallery setting, the average visual intake durations were longer for pseudo-artistic images. Moreover, in the gallery, pseudo-artistic images were associated with shorter average saccade durations across both visits, along with significantly longer total viewing times per image. These interesting reversed patterns between contexts will be further examined in the Discussion section.

Results of the EEG analyses.

The EEG recordings were performed only in the laboratory setting. The initial comparison of resting-state EEG connectivity—recorded prior to the gallery and laboratory sessions—revealed no significant group differences across any of the investigated frequency bands. The maximum U-statistic values and minimum *p*-values observed for all electrode pairs in each band indicated the absence of baseline connectivity disparities between groups. This finding rules out the possibility that any connectivity differences observed during image viewing could be attributed to pre-existing group variations.

In contrast, the comparison of EEG connectivity during image viewing revealed significant group differences in two of the five analysed frequency bands: beta 1 and gamma. Specifically, beta 1-band connectivity was significantly stronger in participants exposed to the artist’s images, whereas gamma-band connectivity was weaker in this group compared to the pseudo-art exposed group. No significant differences were detected in the remaining EEG bands. The graph-theoretic analyses also showed significant differences between the analysed groups. In the beta 2 band significant differences were found for all three graph measures: global efficiency and modularity appeared to be higher for artist images, while clustering coefficient was lower for artist images. Similar pattern was also found for gamma band where global efficiency was higher for artist images and cluster coefficient was lower. Comprehensive statistical details are provided in S1 Appendix.

Thus, viewing artistic or pseudo-artistic images by two different groups of participants revealed, in laboratory settings statistically significant differences in eye movements, EEG network organisation and aesthetic experience. In the gallery the differences were found only for eye movements which exhibited reversed pattern as compared to results obtained in the laboratory.

Since our participants were unaware that one set of images was actually generated by the machine learning algorithms, it can be assumed that the differences were due to different processing of the stimuli from the two exhibitions and two experimental settings. To investigate whether this might be due to their different topological properties, we examined the persistent homology of images. Then, we compared the spatial distribution of the topological properties of the images with the fixation heat maps.

### 3.3 Topological analysis

**Fig 12:**
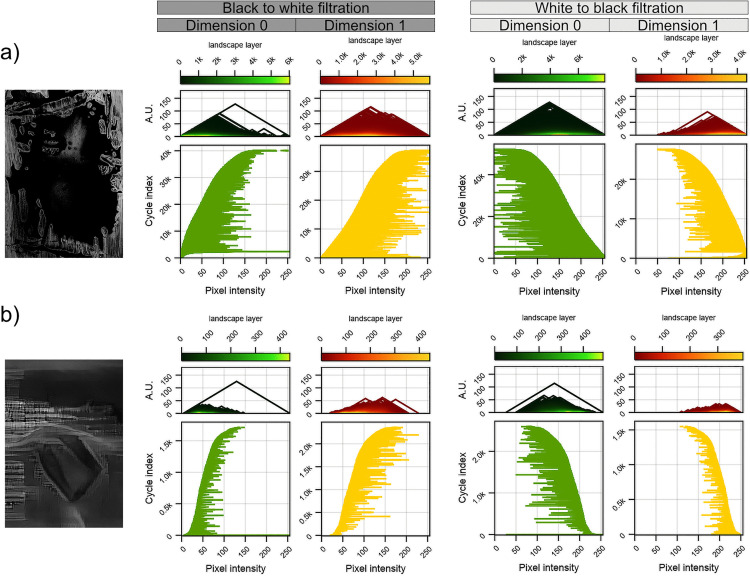
Examples of topological properties of art [11] (a) and pseudo-art images (b). Left column: greyscale-converted original image; middle section: topological results for filtration from black to white; right section: topological results filtration from white to black; within each section – left column: topological characteristic in dimension 0 and right column: topological characteristics in dimension 1. The topological characteristics, the same for both dimensions, are: persistence landscapes(top) and barcodes (bottom). The horizontal axis for both characteristics is pixel intensity filtration steps (in the range [5,255]). Vertical axes are: for persistence landscapes – arbitrary units; for barcodes – index of a barcode in the birth sorted list of all barcodes for an image. Every landscape (for every dimension) is constructed from a different number of layers – the layers are coloured according to the legend above each landscape. The barcodes for the pseudo-artistic images are significantly shorter than for the artistic images, which shows a smaller persistence of the former. As can be read from the landscape diagrams, the pseudo-artistic images contained significantly fewer holes and cycles indicting the far richer structure of the artistic image.

In the first part of our topological analysis, we note that visual inspection of the landscapes of the artist’s images showed that the barcodes spanned almost the entire range of filtration parameters, indicating the existence of topological cycles with high persistence (a sample is shown in Fig 12a; see S5 Fig for all images).

**Fig 13:**
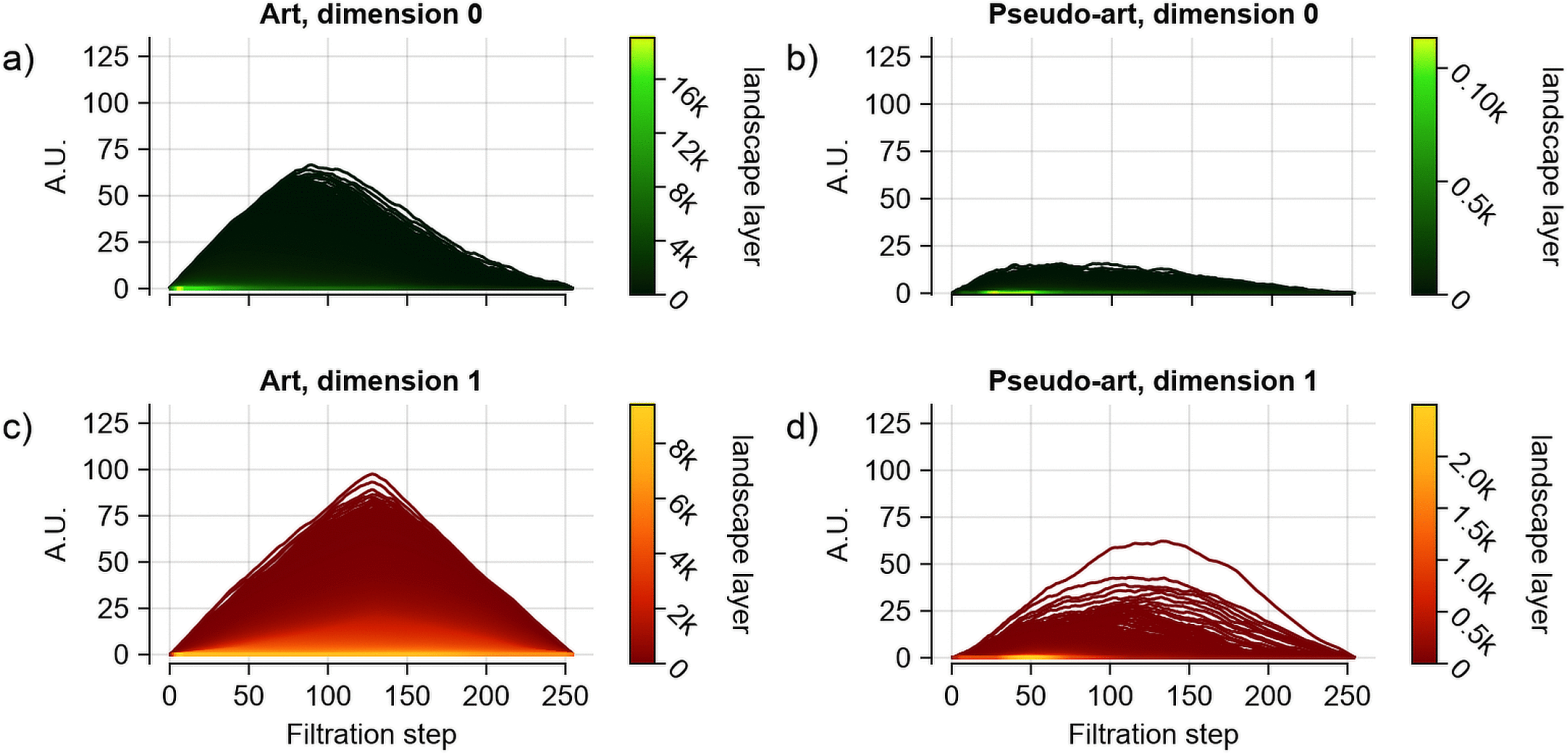
Average group persistence landscapes for BW filtration. The average persistence landscape was computed for each group: art (a,c) and pseudo-art images **(b,d)**, with results for dimensions 0 shown in the top row and dimension 1 in the bottom row. Every individual landscape was constructed from cycles of persistence greater than 5 pixel intensity values [54]. The average landscapes for artistic and pseudo-artistic images were significantly different for each dimension under the non-parametric Wilcoxon test with permutation, both *p* < 0.001. A marked difference in structural richness between the two groups of images is again evident, as persistence landscapes in both dimensions contain significantly more layers for artistic images.

For pseudo-artistic images, however, the barcodes were shorter and born in a narrow range, indicating lower persistence as compared to the art images, as shown in an example in Fig 12b (the visualisation of properties for all pseudo-artistic images is shown in S6 Fig). Also, the number of cycles for pseudo-artistic images was lower by an order of magnitude than that for artistic images in both dimensions (the mean numbers of cycles were: $mean_{d=0}^{Art}=29046\pm 21542$, $mean_{d=1}^{Art}=26775\pm 14941$, $mean_{d=0}^{AG}=1725\pm 1501$, $mean_{d=1}^{AG}=2206\pm 1462$, where the subscript *d* is used to denote dimension); this is reflected in the landscapes by the number of layers (Fig 13).

**Fig 14:**
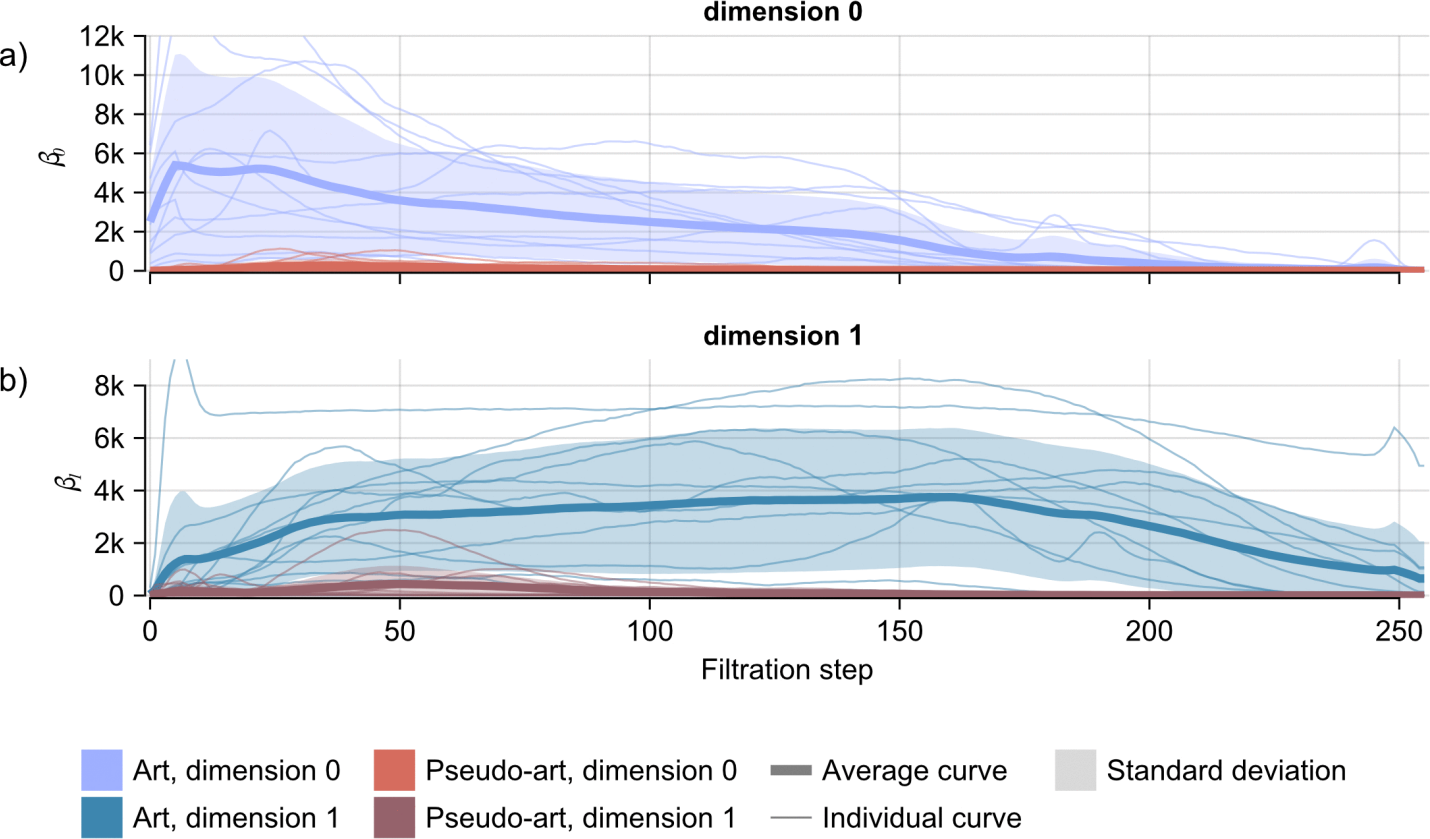
Average Betti curves with 1 standard deviation above and below shaded, together with individual Betti curves for each image for the BW filtration. The average Betti curve was computed for each group: artistic and pseudo-artistic images, with results for dimension 0 (a) and dimension 1 **(b)**. S8 Fig shows Betti curves for the WB filtration. The numbers of holes and cycles reflected here by the Betti curves is significantly different for artistic and pseudo-artistic images.

To statistically assess these apparent differences, we averaged the landscape heights of each layer at each filtration step separately over each set of images. The results are shown in Fig 13. A visual comparison of the averaged topological properties of the artistic and pseudo-artistic images already shows a stark difference. The permutation test yielded highly significant differences between the two groups with *p* = 0.0011 and *p* = 0.0014 for dimension 0 and dimension 1, respectively (10000 permutations). Furthermore, as shown in Fig 14 the Betti curves have dramatically different forms with very little overlap between the two groups for the majority of the filtration steps.

So far, the study of persistence landscapes from one filtration was sufficient to capture the differences between topological properties across all images, for example, average landscapes from the BW filtration are sufficient to describe topological differences between the two groups of images.

The same differences can be observed for the WB filtration, for example in Fig 12 the persistence landscapes for WB filtration dimension 0 are similar in shape to those for BW filtration dimension 1 (the influence of Alexander duality can be seen in the reflected bar codes and landscapes for all images, both art S5 Fig and pseudo art S6 Fig, in S8 Fig we show Betti curves for WB filtration and in S7 Fig we show average persistence landscapes for WB filtration).

Therefore, by Alexander duality, it is possible to capture nearly all topological structures in a single image by using only a pair of topological structures- same filtration, both dimensions, or a selected dimension and both filtrations. The latter approach (with dimension 1 cycles) is later utilised in the feature map construction.

### 3.4 Violations of Alexander duality

As shown in Fig 12, although the columns displaying barcodes and landscapes for the BW and WB filtrations largely mirror each other, they are not perfect reflections and exhibit discrepancies, indicating violations of Alexander duality.

Under perfect duality, every dimension-1 persistent cycle in the forward filtration (for example, BW) should correspond to a dimension-0 persistent cycle in the reverse filtration (for example, WB). Violations occur when cycles intersect the image frame.

**Fig 15:**
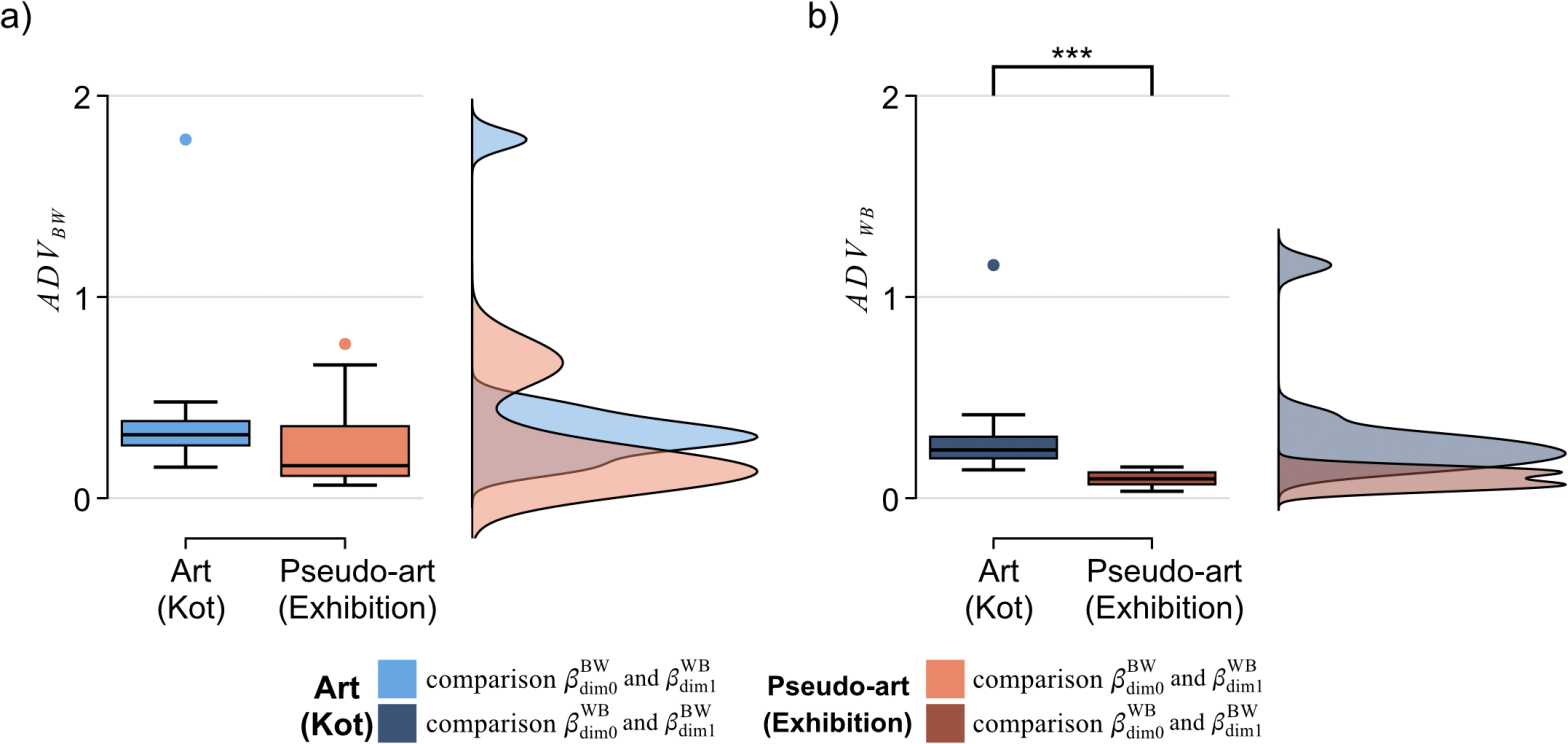
The distribution of violation of Alexander duality demonstrated as the normalised differences in the area under the Betti curve for (a) black to white filtration $\beta_{dim0}^{BW}$ and $\beta_{dim1}^{WB}$ and (b) white to black filtration $\beta_{dim0}^{WB}$ and $\beta_{dim1}^{BW}$, images used in exhibition – art [11] and pseudo-art. The vertical axis is Alexander duality violation measure, that is, the difference between the area of the Betti curve one filtration in dimension 0 and the area of the Betti curve reverse filtration in dimension 1, then normalised by their mean area, as in Equation 1. There is a significant difference between the two groups in the second comparison (Mann-Whitney U test, (a) and **(b)** – statistic value: 103 and 140; two-sided: *p* = 0.078 and *p* < 0.001; point estimate: 0.1536 and 0.1435; rank sums: (181, 119) and (218, 82); number of observations in each group: 12 and 12). The statistics for the distributions are presented in S3 Table.

To quantify these violations, we compared the distribution of the differences in the area under Betti curves (see Equation 1), linked by Alexander duality, for both art and pseudo-art as described in Section 2.6.3. The results are presented in Fig 15.

This investigation revealed that, apart from differences shown for every dimension, such as differences in persistence landscapes and Betti curves, there are also differences (one trending and one significant) in the extent to which Alexander duality is violated, indicating that art images have more cycles that have no counterparts under Alexander duality. The asymmetry in the significance is due to one anomalous pseudo-art image with an unusually high value of *ADV*_*BW*_. It shall be shown in Section 3.6 that this result generalises in a way that is symmetric with regard to the duality violation measures *ADV*_*BW*_ and *ADV*_*WB*_.

### 3.5 Comparison of the persistent homology with current state-of-the-art methods

To evaluate the ability of topological versus conventional statistical features to discriminate between image sets, we performed a series of comparative analyses. We chose two simple summary statistics derived from the persistent homology computations, namely, total landscape area in dimensions 0 and 1. This, of course, captures a fraction of the richness of the topological approach (for example, no shape information). We compared these to a set of metrics frequently used in artistic image analysis, including Fourier slope, self-similarity, complexity, anisotropy, and edge density. To provide a more comprehensive assessment, we also examined basic statistical descriptors such as mean, median, standard deviation, kurtosis, and skewness. The non-parametric Wilcoxon test was applied to determine whether differences between groups were statistically significant. For metrics that showed significant effects, we quantified the magnitude of group separation using Hedges’ g, an effect size measure that reflects the difference in terms of standard deviation units. The results are shown in Table 2.

**Table 2:** Summary of Hedges’ *g* effect sizes and Kruskal–Wallis tests.

Parameter	Dim 0	Complexity	std	Edge-density	Dim 1	Fourier Slope	Skewness	Kurtosis
Hedges’ *g*	1.73	1.64	1.59	1.37	0.91	0.89	0.72	0.66
*p*‑uncor.	<0.001	<0.001	0.002	0.006	0.001	0.046	0.033	0.003
test stats	10.82	10.83	9.363	7.68	10.45	10.45	4.083	0.013

The comparison of metrics revealed that the strongest separation between the analyzed image sets was achieved using the persistent homology method. Interestingly, among the eight metrics that produced significant results, some simple statistical measures—such as the standard deviation and skewness—also demonstrated strong performance. However, while these simple statistics are inherently single-value descriptors, more complex metrics like those derived from persistent homology, edge density, and complexity capture richer information about the structural properties of the images. For the purposes of this analysis, these complex metrics were reduced to single-value representations to enable direct comparison.

### 3.6 Extending comparisons to other abstract artists

In the final step of our comparisons between art and pseudo-art, we sought to verify whether the proposed method and tools generalise to other abstract artists representing diverse styles and other non-intentionally produced pseudo-artistic images. To this end, we selected five renowned figures representing different styles of abstract painting: Kandinsky – Spiritual Abstraction, Rothko – Color Field Painting, Malevich – Suprematism, Jarema – Abstraction with Surrealist and Theatrical Influences, and Pollock – Abstract Expressionism / Action Painting (referred to as ‘All Art’). For each artist, we collected 12 publicly available images of resolution comparable to that used in previous analysis (we include lists with the titles of the paintings used in S6 Appendix. For comparison, we used all 4500 images generated by perturbed BigGAN networks (referred to as ‘All Pseudo-art’).

We chose Alexander duality violation as the method of comparison as it elegantly combines both dimensions of persistent homology (Betti curves of dimension 0 and 1) and both directions of filtration. We also hypothesized that while images generated by randomly perturbed GANs should largely respect Alexander duality, images created by artists should deviate from it, reflecting the individual characteristics of the artists and their ideas about framing compositions.

**Fig 16:**
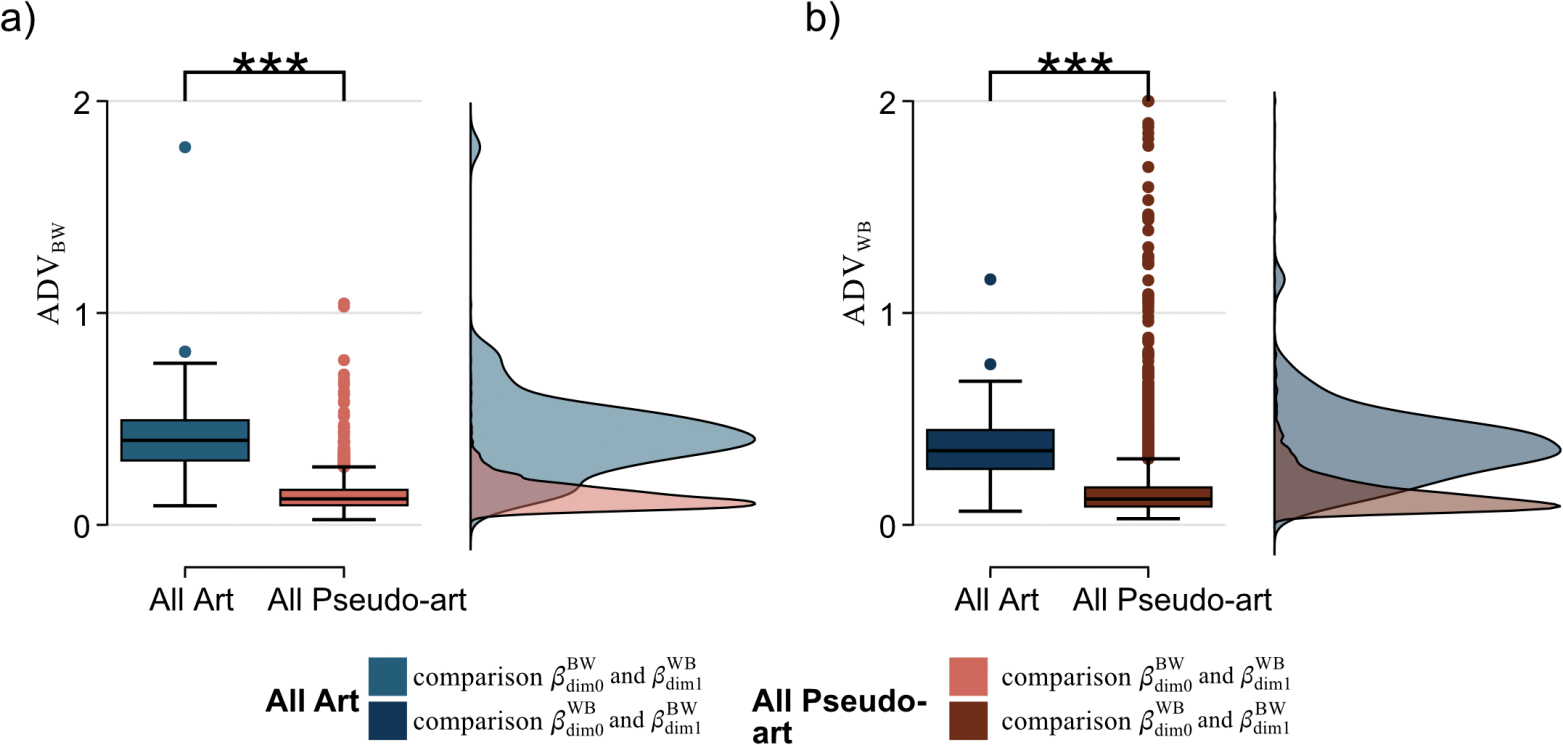
The distribution of violation of Alexander duality demonstrated as the normalised differences in the area under the Betti curves for: for (a) $\beta_{dim0}^{BW}$ and $\beta_{dim1}^{WB}$ and (b) $\beta_{dim0}^{WB}$ and$\beta_{dim1}^{BW}$, all art images and all pseudo-art. The vertical axis shows the Alexander duality violation measure *ADV*, as defined in Equation 1. The data presented are art images extended by a group of paintings of popular abstract painters (we include a list of paintings in S6 Appendix) and all 4500 generated pseudo-art images. There is a significant difference between the two groups in both comparisons (Mann-Whitney U test, (a) and **(b)** – statistic values: 356695 and 338429; two-sided: *p* < 0.001 and *p* < 0.001; point estimate (median of all possible pairwise differences): 0.2764 and 0.2282; rank sums: $\left(0.360\times 10^{7},1.014\times 10^{7}\right)$ and $\left(0.341\times 10^{7},1.016\times 10^{7}\right)$; number of observations in each group: 844500 and 844500). The statistics for the distributions are presented in S3 Table.

The results are presented in Fig 16, where we see that for the ‘All Art’ images, the violation of Alexander duality is significantly greater than for ‘All Pseudo-art’. The results are significantly different (*p* < 0.001) under the Mann-Whitney U test. The positive difference means that artists include a higher proportion of structures in the frames of their images as compared to BigGAN generated pseudo-art. This generalises the observation seen with the work of Kot (art) vs the matched pseudo art, as shown in Fig 15.

### 3.7 Topologically derived properties

To systematically decompose abstract images, we focused on three topologically derived properties: persistence, the density of cycles, and the perimeter of 1-dimensional cycles. Cycles are representatives of each topological invariant (homology class) identified by the method. Long-lived or ‘persistent’ cycles are objects that are the most salient in terms of the filtration and are, therefore, the easiest to see, i.e., they are bounded by pixels with a large difference in contrast. The ‘richness’ of an image, i.e., the number of individual structures such as holes and cycles when changing the filtration parameter, is revealed by the Betti curve and the number of layers in the persistence landscape. Cycle density can be considered a coarse proxy for texture (two distinct textures may have the same cycle density but consist of differently shaped cycles). Cycle perimeter is a measure of spatial extent, as in general, cycles with large spatial extent will have a larger perimeter (in general, it will differ according to fractality present in the image). Cycle perimeter, therefore, reveals mesoscale spatial structures that compose an image.

### 3.8 Topologically driven eye movement patterns

To examine the putative relationship between viewers’ eye fixation patterns and the topological properties of the images, we used empirical cumulative distribution functions (ECDFs). Spatial distributions of key topological features—density, maximal persistence, and cycle perimeter—were analyzed through the computation and comparison of ECDFs. To assess the robustness of these distributions, we evaluated the effect of varying window sizes, which had minimal influence on the shape of the ECDFs (see S30 Fig). This suggests a scale-invariant property with respect to window size and confirms that the distinction between the two image sets remains unaffected. Ultimately, we identified significant differences in the density distributions between the artistic and pseudo-artistic image sets.

**Fig 17:**
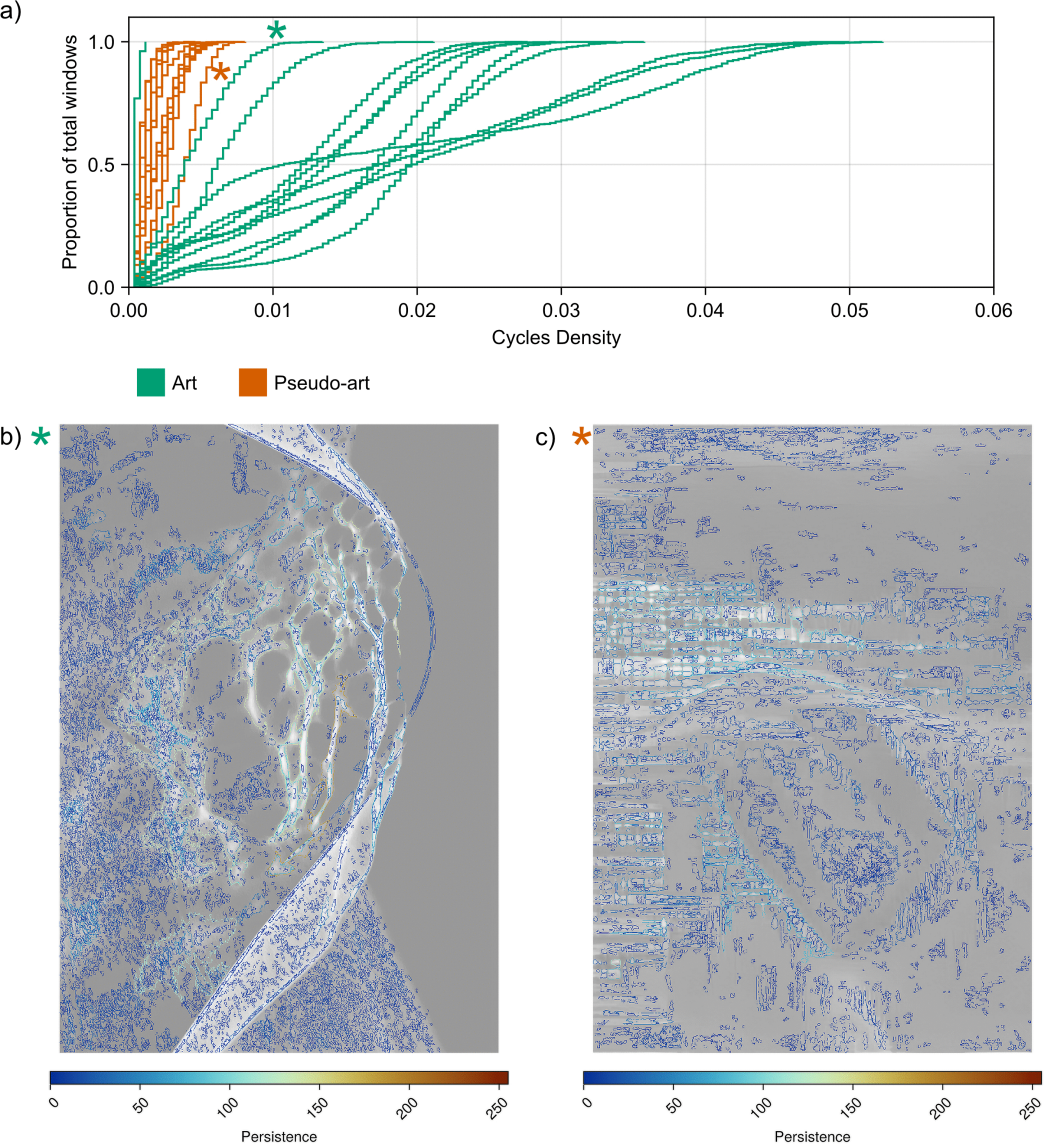
Cycle density comparison for all images (a) and the two closest cross-group samples of cycles visualisation (b,c). Every curve in (a) represents the ECDF of the cycle density distribution for images for both exhibitions; the density used for ECDF is the sum of densities from BW and WB filtrations; artistic (green) and pseudo-artistic (orange). The vertical axis is the proportion of all windows from a grid mesh. All curves were statistically different under the Kolmogorov-Smirnov test. Images for which cycles were visualised are those marked with ‘*’ in the figure with ECDF plots. The cycle density reflects the texture of an image, i.e., the spatial distribution of changes in contrast.

Fig 17 shows ECDF curves for cycle density (both filtrations) computed for each of the 24 analyzed images, highlighting the discernible disparities between the two categories resulting in an almost clean separation (more details are presented in S31 Fig). A clear separation could not be achieved for the ECDFs of maximal persistence (shown in S32 Fig) or for those of cycle perimeter (shown in S33 Fig).

In the next step, we investigated whether the observed differences in the topological properties of the two image sets corresponded to distinct relationships between these properties and the eye movement patterns recorded during image viewing.

First, we wanted to determine whether participants paid attention to the same topological properties in the artistic and pseudo-artistic images. To this end, we overlaid individual fixation heatmaps onto the topological feature maps generated for each image and computed mean squared errors (MSEs) between the ECDF derived from the same feature map weighted by the fixation heatmaps and ECDFs derived from the image’s feature map (i.e., weighted by a uniform gaze, $ECDF(M_{k},U)$). We then compared the resulting MSEs of the ECDF curves (see Methods section for details) of the two groups.

**Fig 18:**
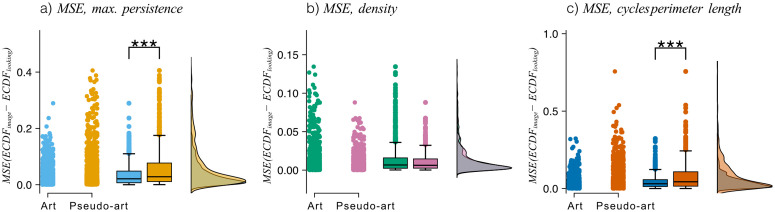
Comparison of how topological features were explored in both exhibitions. **MSE between image features distribution ECDF and the features weighted by each participant’s gaze duration data.** MSEs between two ECDFs can only lie between 0 and 1. Results for: **(a)** Persistence; **(b)** Cycle density; **(c)** Cycle perimeter. (Exhibitions are indicated on the axis; only laboratory data was used for this comparison). The line-up of plots within each subfigure is as in Fig 11. The Mann-Whitney U test, comparing MSE distribution for the artistic images to MSE distribution for the pseudo-artistic images, yielded significance for a) and **c)**, *** *p* < 0.001.

The results shown in Fig 18 indicate that averaged MSEs calculated for maximal persistence and maximum cycle perimeter were significantly higher for pseudo-artistic images than for artistic ones. In contrast, no significant difference was observed for MSEs corresponding to differences in the cycle density ECDFs (see Table 3 below for the results of the Mann-Whitney U tests).

**Table 3:** Results of Mann-Whitney U test.

Property	*U*	Median (art)	Median (pseudo-art)	*p*
Max. Persistence	196250	0.0211	0.0284	<<0.001
Density	245019	0.0066	0.0062	0.346
Cycle perimeter	196624	0.0309	0.0436	<<0.001

In all cases from Fig 18 $n_{Art}=693$ and $n_{A.G.}=687$.

**Fig 19:**
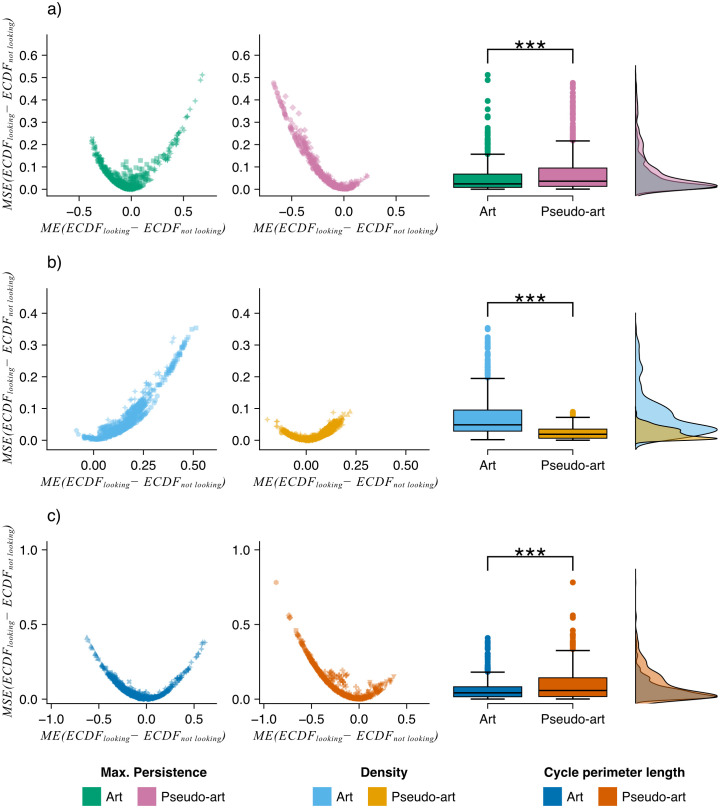
Analysis of topologically derived features that participants were attracted to. Each point on the plots is derived from the ECDFs obtained from a single participant viewing a single image. For every image and every participant who viewed the image, a weighted feature map distribution was obtained from: (1) the regions where the participant looked (weighted by gaze duration) and (2) the regions where the participant did not look (uniform weighting). (a) results for persistence feature maps. (b) results for cycle density feature maps. (c) results for cycle perimeter derived maps (exhibitions are indicated in the legends). In each plot in the first two columns, we show MSE on the vertical axis and ME on the horizontal axis, both computed from the difference $ECDF_{looking}-ECDF_{not\;looking}$ for each participant, i.e., it compares the ECDF of regions that a participant looked at with the regions that the same participant did not look at. Note that the MSEs between two ECDFs can only lie between 0 and 1, whereas the ME can lie between −1 and 1. The third column shows the distribution of MSEs on which significance tests were conducted. Statistics performed with Mann-Whitney U test, comparing MSE distribution of ECDF between the artistic and pseudo-artistic images, *** *p* < 0.001.

Next, we sought to identify whether participants are drawn to specific topologically derived properties within a viewed image. To this end, we compared the distributions of persistence, cycle density, and cycle perimeter of the image area covered by each person’s gaze heatmap (‘looking’ $ECDF(M_{k},G_{s})$ for person *s* looking at image *k*) with the distributions for the same derived properties but of the uniformly weighted areas outside the person’s gaze heatmap (‘not looking’ $ECDF(M_{k},\tilde{G_{s}})$). The MSE between these two ECDFs was computed and plotted for each viewing of each image by each participant and plotted in Fig 19.

The results indicated that pseudo-artistic images had significantly higher MSE for maximal persistence than the artistic images (Mann-Whitney U test, two-sided *p*<<0.001; Fig 19). At the same time, a significant difference was observed for MSE of the cycle density (Mann-Whitney U test, two-sided *p*<<0.001) and for cycle perimeter ECDF (Mann-Whitney U test, two-sided *p*<<0.001). All details about the tests are presented in Table 4.

**Table 4:** Results of Mann-Whitney U test for MSE of ECDF for looking and not looking.

Parameter	*U*	Median (art)	Median (pseudo-art)	*p*
Max. Persistence	200488	0.0240	0.0362	<<0.001
Density	377815	0.0486	0.0189	<<0.001
Cycle perimeter	204175	0.0423	0.0578	<<0.001

In all cases from Fig 19 $n_{Art}=693$ and $n_{A.G.}=687$.

Summarizing the analyses of the topological properties being of special interest for artistically oriented participants, we found that both groups preferentially fixated on the image areas characterized by higher persistence as shown by S34 Fig. Here, the blue ECDF for each image, $ECDF(M_{k},U)$, is intrinsic to the image itself and can be thought of as arising from a ‘gaze’ that is a uniform scan of the entire image. These results appear to correspond with the differences in fixation duration observed in the laboratory setting, where participants exhibited significantly longer average fixations on artistic images – images that also displayed higher homology persistence across both dimensions. However, the reverse was observed in the gallery, where the perception conditions of the images changed with the movement of the participants, introducing an element of filtration not available under laboratory settings.

In the case of cycle density, the preference for areas of higher density was found only in the group viewing the pseudo-artistic, artificially generated images. This pattern, together with viewers’ stronger preference for features such as topological persistence and cycle perimeter in pseudo-art images (see Table 5),was further accentuated in the gallery setting through natural perceptual filtering. This may account for the higher average visual intake duration observed for these images (see S35 Fig and S36 Fig for all ECDFs for cycle perimeter and cycle density, respectively).

**Table 5:** Summary of image property preferences from Fig 19.

Property	Art	Pseudo-art
Max. Persistence	No clear preference	**Preference for more persistent cycles**
Cycle Density	**Preference for lower density**	No clear preference
Max. Cycle Perimeter	No clear preference	**Preference for cycles with greater spatial extent**

Notably, viewers’ preferences for specific topological features appear to be closely linked to the overall topological structure of the images, highlighting a relationship between visual perception and underlying image topology.

Note that the contents of Table 5, presented for convenience, can be gleaned from Fig 19 by the following reasoning steps: if $ME(ECDF_{looking}-ECDF_{not\;looking})>0$, then $ECDF_{looking}$ mostly lies below and to the right of $ECDF_{not\;looking}$ which will mean that the features being looked at will have higher values (of whichever property) compared to the features ignored. ‘Smiles’ or parabolic shapes denote ‘no clear preference’; asymmetric smiles with only a right (left) side signify a preference for low (high) values.

### 3.9 Influence of topological features on subjective perception

In the preceding section, we demonstrated the relationship between topological features, namely maximum persistence, cycle density and maximum cycle perimeter length, and patterns of eye movements. Building on these findings, we next examined how these features influence subjective perceptual experiences. Given that persistent homology captures the underlying structural organization of visual forms, we focused on participants’ laboratory responses to the AEQ–S questionnaire item “It is easy for me to identify and recognize the significance of individual elements (colour, composition, etc.)”—hereafter referred to as Elements—which then served as the reference category for the remaining dimensions of the flow questionnaire (Emotions, Intentions, Context, Understanding, and Flow).

To this end, we fitted a linear mixed-effects model (LMM) separately for each standardised (*z*-scored) topological predictor to assess their main and interaction effects with the categorical factor Measure, consisting of all six items of the flow questionnaire, on the dependent variable Score. The model incorporated random intercepts for Image (stimulus-level variability) and for Participant ID (individual-level variability), thereby accounting for the hierarchical structure of the data. To account for the general effect of the topological features, we included all 24 images (both art and pseudo-art) and AEQ-S ratings provided by all 58 participants in the model. Model estimation was performed using Restricted Maximum Likelihood (REML) with the Powell optimisation algorithm to ensure convergence. The model can be expressed as (note that it is common for both Betti numbers and coefficients in REML to use the same Greek letter; to avoid confusion, we use β for the former and β for the latter with $\hat{\beta }$ for its estimates):


$$\begin{array}{l} {\text{Score}}_{ij}=\beta_{0}+\beta_{1}\;{\text{TopologicalFeature}}_{ij}+\sum_{k=1}^{5}\beta_{2k}\;{\text{Measure}}_{k,ij}\\ \;+\sum_{k=1}^{5}\beta_{3k}\;({\text{TopologicalFeature}}_{ij}\times {\text{Measure}}_{k,ij})\\ \;+u_{0i}+v_{0j}+\varepsilon_{ij} \end{array}$$ (2)

where:

- ${\text{Score}}_{ij}$ is the standardized rating for participant *i* and image *j*,
- ${\text{TopologicalFeature}}_{ij}$ denotes a standardized predictor (either maximum persistence, density scaled or cycle perimeter),
- ${\text{Measure}}_{k,ij}$ are dummy-coded questionnaire dimensions (the 5 values of *k* are Intentions, Flow, Understanding, Emotions, Context; with Elements as the reference),
- $u_{0i}\sim 𝒩(0,\sigma_{u}^{2})$ is the random intercept for participant *i*,
- $v_{0j}\sim 𝒩(0,\sigma_{v}^{2})$ is the random intercept for image *j*,
- $\varepsilon_{ij}\sim 𝒩(0,\sigma^{2})$ is the residual error term.

**Fig 20:**
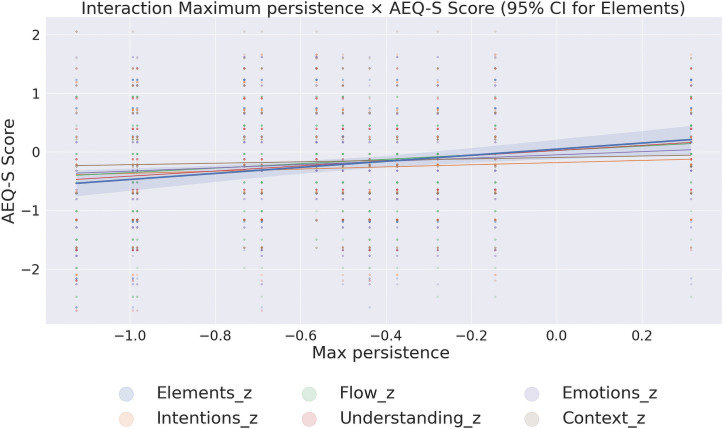
Linear mixed-effects regression for Cycles density and AEQ–S questionnaire categories with Elements being the reference item for all images (art and pseudo-art) and all 58 participants.

Results for the model including cycle density as a predictor appeared to be the strongest and showed a significant positive main effect of persistence on subjective scores ($\hat{\beta }=0.265$, *SE* = 0.037, *z* = 7.147, *p* < .001), indicating that images with higher cycle densities were generally rated higher across all perceptual measures. This effect represents a medium, domain-general influence. A significant interaction with Context ($\hat{\beta }=-0.096$, *SE* = 0.033, *z* = −2.908, *p* = 0.004) was observed, suggesting that the positive effect of cycle density on subjective ratings was attenuated by the above item. No other measure-specific interactions reached statistical significance (see Fig 20).

**Fig 21:**
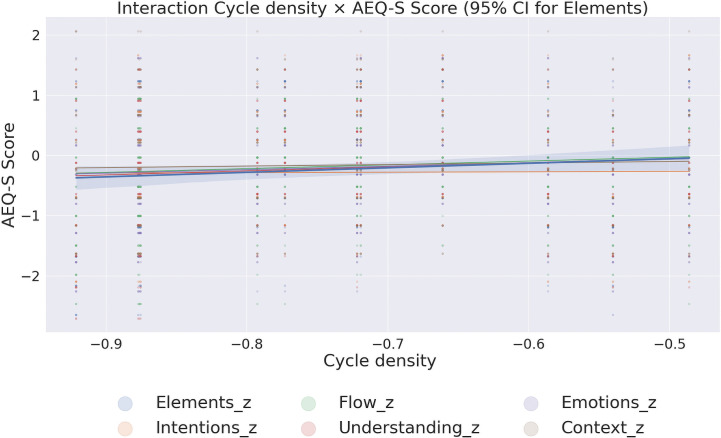
Linear mixed-effects regression for Maximum persistence and AEQ–S questionnaire categories with Elements being the reference item for all images (art and pseudo-art) and all 58 participants.

Also, significant, although not as strong, results were obtained for the maximum persistence ($\hat{\beta }=0.147$, *SE* = 0.038, *z* = 3.880, *p* < 0.001), indicating that images characterised by greater maximum persistence were associated with higher subjective ratings across all measures. This effect represents a small general influence. A significant interaction with any other items were not observed, suggesting that the positive effect of maximum persistence on subjective ratings was not affected by other questionnaire dimensions, see Fig 21. Cycle perimeter showed no significant effects.

When considered together, both topological descriptors — density and persistence — showed significant positive associations with subjective perceptual ratings, indicating that visual structures with richer and more stable topological organisation were generally perceived more favourably across evaluative dimensions.

## 4 Discussion

Understanding the complex interaction between art and human perception has long fascinated researchers. Over the past 150 years, numerous efforts have been made to employ scientific methods to decode the human aesthetic experience [1,55–59]. Within visual art, research has predominantly focused on the relationship between image properties and both subjective human experience and objectively measurable behavioural or neural responses to viewing such images [56,60–62].

A consistent body of evidence shows that brain regions respond more strongly to objects defined by luminance, texture, illusory contours, motion, stereo, or colour than to random stimuli or uniform textures derived from the same cues [63–68]. Intriguingly, holistic representations of objects are recognized earlier than their constituent parts [69–73], a finding confirmed by numerous neuroimaging studies using both EEG (for a review, see [74]) and fMRI (for a review, see [75]).

Despite these advances, the relationship between statistical image properties (SIPs) of an artwork and the subjective experience it elicits remains weak or, at best, moderate [3]. This gap highlights the need for analytical approaches that more effectively capture the perception of shape and structure in visual art and their connection to human response. To address this, we employed persistent homology [10], a method that systematically characterizes topological structures of an image across multiple spatial scales.

Our findings revealed significant differences between artistic and pseudo-artistic images. These differences manifested first in persistence barcodes—capturing the stability of topological features—and second in topologically derived descriptors, such as cycle density (Fig 17). These descriptors provided greater discriminative power than conventional SIPs (2), revealing richer insights into the images’ structural organization.

Furthermore, the mathematical duality inherent in constructing “opposite” filtrations of cubical complexes captures additional image characteristics. Since this duality holds only for cycles that do not touch image boundaries, it inherently encodes the influence of framing—one of the most crucial aspects of image composition. Artists have long placed key visual elements in relation to geometric divisions within the frame [76,77]. Considering the significance of framing, its topological representation is of particular artistic and analytical interest.

Our Alexander duality–based comparison between canonical abstract paintings and 4,500 pseudo-artistic images generated by randomly disturbed GANs revealed a clear distinction between these two sets. Interestingly, the works of eminent abstract painters converged toward a specific level of Alexander duality violation around 0.4, expressed as a dimensionless value derived from topological invariants (areas under Betti curves; Fig 16). This may suggest that artists intuitively adhere to a compositional balance analogous to the “golden rule”, and that this rule itself can be expressed through persistent homology—thus offering a mathematical foundation for classical principles of visual composition.

The advantage of employing paired, opposing filtrations (from low to high pixel values and vice versa) is that the union of the sets of dimension 1 cycles from both filtrations provides a comprehensive representation of salient features, as well as providing a uniform way to measure spatial extent (via cycle perimeter). Using both dimension 0 and 1 cycles would not allow this. This approach allows for systematic interrogation of image structures across multiple spatial and intensity scales.

An important part of our study was to explore the relationship between topologically derived image features, eye movement, and aesthetic experience. As noted in the Methods section, we avoided direct comparisons between the laboratory and gallery settings to prevent confounding effects arising from different display modalities. Consequently, all analyses examining correlations and dependencies between eye-tracking measures and topological image properties were conducted exclusively using laboratory data. We examined whether regions fixated on by viewers differed structurally from those ignored. As shown in Fig 19 and summarized in Table 5, when viewing artistic images, participants exhibited no clear preference for regions of maximum persistence or cycle perimeter but consistently preferred areas with lower cycle density. In contrast, during pseudo-art viewing, participants favoured regions with higher persistence and larger spatial cycles, showing no consistent preference regarding cycle density. These findings imply that the perceptual importance of specific image features depends on the overall image context, challenging studies that propose absolute preferences for particular features (for example, complexity [78,79]).

Regression analyses between topological features and AEQ-S questionnaire categories, also performed on laboratory data, confirmed a relationship between image structure and aesthetic response. Two of three features, cycle density and maximum persistence, were positively correlated with the “Elements” score, supporting the idea that topological organization influences human aesthetic experience. Thus, topological descriptors not only distinguish art from pseudo-art but also meaningfully relate to subjective experience, positioning them as tools for the analysis of artistic imagery.

However, only the results from the laboratory experiment fully confirmed our hypothesis stating that intentionally generated artistic images will elicit different aesthetic and eye-tracking responses when compared to those elicited by pseudo-art, which is deprived of any human intent. The longer fixation durations observed in the laboratory for artistic images likely reflect heightened visual interest and deeper cognitive processing [80,81]. This interpretation is further supported by the AEQ-S questionnaire results, which showed consistently higher ratings across all six categories for the artistic images. The weaker gamma-band connectivity observed during art viewing may initially appear contradictory, given the association of gamma activity with visual attention [82,83] and art perception [84]. However, gamma oscillations have also been linked to the integration of ambiguous visual information and perceptual switching (for review see Ghiani et al. 2021 [85]). In this context, increased gamma connectivity in the pseudo-art condition may reflect greater perceptual uncertainty rather than enhanced engagement. This interpretation aligns with findings by Yuval-Greenberg et al. (2008) [86], who associated induced gamma activity with increased saccade rates. Saccades were also more frequent during pseudo-art viewing in our study, suggesting a more exploratory viewing strategy. Further supporting the notion of greater attentional engagement during art viewing is the stronger beta-1 band connectivity observed in these participants. Beta activity has been linked to top-down projection of visual representations from early visual areas to the prefrontal cortex, facilitating the formation of initial interpretative hypotheses about visual input [87]. Taken together, the laboratory findings suggest that viewing artistic images involves more stable and integrative processing consistent with deeper aesthetic engagement.

In contrast, the gallery data revealed longer visual intake durations for pseudo-artistic images during the first visit, accompanied by consistently shorter average saccade durations and significantly longer total viewing times per image across both visits. Interestingly, comparison of the AEQ-S questionnaire responses between the two exhibitions conducted in the gallery showed no significant differences. This apparent paradox most likely arises from environmental differences between laboratory and gallery settings. In the laboratory, static and controlled lighting conditions enhanced contrast, stabilizing the detection of highly persistent topological features in artistic images. In the gallery, however, illumination and continuous viewer movement introduced a form of natural perceptual filtering, altering the visibility of specific topological features over time. Because the same print technology was used for both image sets, micro-scale surface variations that might otherwise modulate light reflection were largely suppressed; consequently, differences in colour coverage did not translate into pronounced changes in surface texture or reflection angle. Under these conditions, pseudo-art images characterized by lower persistence may have produced a subtle “flickering” perceptual effect, as topological cycles intermittently appeared and disappeared with changing lighting and viewing angles. This transient instability—particularly salient in peripheral vision—may have drawn and sustained viewers’ attention despite the images’ lower global persistence.

Although Pelowski’s study [88] found no significant impact of lighting on aesthetic experience, its stable illumination conditions and lack of topological or eye-tracking analyses limit its relevance here. In contrast, our findings suggest that lighting variation and viewer movement critically shape perception. Historically, artists created works under dynamic illumination and movement, likely exploiting these conditions to influence perception at different times of day or under varying light sources. Presenting artworks under uniform, artificial light may therefore suppress perceptual nuances originally intended by the artist. This distinctive feature of our research – the direct comparison between the gallery and laboratory viewing conditions not only provides a more comprehensive validation of our framework but also reveals clear contextual distinctions in AEQ-S responses and eye-movement patterns. Together, these findings underscore the importance of environmental context in studies of aesthetic perception and suggest that controlled laboratory settings and naturalistic gallery conditions may evoke fundamentally different modes of visual and emotional engagement.

In summary, by analogy with thermodynamics—where macroscopic properties like temperature and pressure emerge from the collective behaviour of microscopic particles—SIPs can be viewed as macroscopic descriptors of an image, with pixels representing microscopic components. Between these scales lies a mesoscale level of organization, comparable to that found in disciplines such as fluid mechanics, where coherent patterns (for example, boundary layers or vortex sheets) arise from coordinated local interactions. We propose that persistent homology implemented through cubical complexes provides a robust framework for analyzing mesoscale visual structures and patterns, bridging the gap between local pixel-based measures and global image statistics. While handcrafted local metrics (for example, edge density) can produce topographic maps, they lack the capacity to systematically capture mesoscale organization. In contrast, persistent homology—combined with fixation-based mappings—offers a coherent framework linking visual structure to perception.

This new approach, along with the toolkit we developed, allows for the following future directions:

1. It provides an approach that takes into account shape preferences in human perception based on objective mathematical methods.
2. It captures the significance of individual image features in relation to the properties of the whole image.
3. Together with appropriate psychophysical experiments, it enables us to grasp the influence of viewer movement and lighting conditions on physiological responses to artistic stimuli.
4. It will enable experimenters to fine-tune their visual stimuli more carefully in experiments and could lead to the development of more interesting derived measures based on this topological decomposition of the visual stimulus. The alignment and arrangement of cycles can lead to new ways of characterising and systematically generating and modulating shapes and textures found in images, for example, violations of Alexander duality.

### 4.1 Limitations

Our method converts images to greyscale using a weighted sum of the RGB channels, which limits our ability to attribute observer responses to specific colours. While the greyscale representation preserves shapes from the original image, it prevents us from disentangling the individual influence of colour on perception. However, for two measures, cycle density and perimeter length, based on the location and size of topological features, greyscale offers a reliable summary, as demonstrated in S16 up to S23 Figs. In these cases, cycles either appear in the same locations across channels or are absent entirely, making greyscale a sufficient representation.

That said, maximal persistence may be influenced by colour-specific intensity, since it depends on high values across all channels (i.e., bright regions). Thus, subtle colour effects might be underrepresented in our framework. Investigating colour as an independent variable is a promising avenue for future work. For instance, recent advances such as the chromatic alpha complex [89] offer the potential for incorporating inter-channel relationships, though current methods are not yet adapted for grid-based image data and would require theoretical development.

Additionally, our psycho-physiological experiment focused on artworks from a single abstract artist, and each participant viewed only one exhibition. Broader generalization would require replicating the experiment with a diverse range of artists and artworks, which is not a trivial task, as famous paintings are held in numerous prestigious galleries around the world. Nonetheless, the single-blind design ensured that participants believed all artworks were human-made. Importantly, despite significant differences in topological features—such as an order-of-magnitude increase in cycle counts and density—no participant questioned the authenticity of the pseudo-art, suggesting strong perceptual plausibility.

Although a full replication of the study with more artists was not feasible, our analysis of a diverse range of paintings produced by eminent abstract artists revealed a significant difference in the violation of Alexander Duality. This finding generalizes the observation initially made when comparing Kot’s work to pseudo-art.

Finally, this study does not yet include additional analyses examining the effects of topology on human perception and aesthetic experience. This would, of course, necessitate a full replication of the psycho-physiological experiment for multiple artists, which would be prohibitively costly.

## Supporting information

S1 Fig Differences in the strength of EEG connectivity (wPLI) between the artistic and pseudo-artistic groups in beta 1 EEG bands. A red colour denotes higher connectivity strength in the artistic group, blue in the pseudo-artistic group. All differences are significant at *p* < 0.05 (FDR corrected).
(PDF)

S2 Fig Differences in the strength of EEG connectivity (wPLI) between the artistic and pseudo-artistic groups in gamma EEG bands. A red colour denotes higher connectivity strength in the artistic group, blue in the pseudo-artistic group. All differences are significant at *p* < 0.05 (FDR corrected).
(PDF)

S3 Fig All artistic images [11]. Original tiles are given under each image.
(PDF)

S4 Fig All pseudo-artistic images. Original tiles are given under each image.
(PDF)

S5 Fig Persistence barcodes and landscapes for the artistic [11] images (BW filtration). (PDF)

S6 Fig Persistence barcodes and landscapes for the pseudo-artistic images (BW filtration). (PDF)

S7 Fig Average group persistence landscapes for WB filtration. The average persistence landscape was computed for each group: Art (a,c) and pseudo-art images (b,d), with results for dimensions 0 shown in the top row and dimension 1 in the bottom row. Every individual landscape was constructed from cycles of persistence greater than 5 pixel intensity values.
(PNG)

S8 Fig Average Betti curves with their standard deviation, together with individual Betti curves for each image for the WB filtration. The average Betti curve was computed for each group: Art and pseudo-art images, with results for dimensions 0 (a) and dimension 1 (b).
(PDF)

S9 Fig An example of duality how duality affects cycles and their lifetime. The image used for black-to-white filtration is presented in the first column, top row; the dual filtration is done with the inverse of the original image, with the filtration again going from black to white (this was done so that it is easier to notice starting points, while the results of the filtration are the same after inverting the horizontal axis on the plots). For both images, results are presented in dimensions 0 and 1 in the form of persistence landscapes, barcodes and Betti curves, as indicated with the labels. The results for dimension 1, top row and dimension 0, bottom row, are the same with respect to inverting the horizontal axis. The results for dimension 0, top row, have 2 more cycles, one is the infinite cycle (which contributes to the outermost layer in the landscape), second missing cycle is related to one of the barcodes starting at step 0 and finishing at step 4 (dimension 0, top filtration).
(PDF)

S10 Fig Comparison of *L*^1^ distance matrices between filtration and demonstration of duality. To justify the sufficiency of only selecting dimension 1 cycles for our analysis, we computed *L*^1^ pairwise distance between all 24 persistence landscapes within each dimension and for both filtrations. The duality between the topological invariants derived from the two filtrations predicts (up to boundary cycles) that these *L*^1^ distance matrices will be almost identical. A: filtration from black to white. B: filtration from white to black. Each heatmap shows *L*^1^, pairwise differences between the persistence landscapes of all images in dimensions 0 (left) and 1 (right). Every row (or column) in the heatmap corresponds to an image annotated between the matrices. The top 12 labels (coloured orange) are from pseudo-artistic images, and the next 12 labels (coloured green) are from artist images. While not all cycles are reflected in the duality, the overall shape differences captured with *L*^1^ distance between landscapes are mirrored when the filtration of the images is inverted. This verifies the expected similarity between the following pairs of matrices: ((1) BW filtration in dimension 0 and WB filtration in dimension 1; (2) BW filtration in dimension 1 and WB filtration in dimension 0). This is a powerful demonstration of the duality.
(PDF)

S11 FigEffect of rescaling all the images on the persistence features, shown as the trajectory of changes in areas under the landscapes in dimensions 0 and 1.The horizontal and vertical coordinates are areas under the landscape in dimensions 0 and 1, respectively. A: results for artistic images. B: results for pseudo-artistic images. Persistence properties were computed for each image (marked with different colours) after resizing (size indicated by markers), creating “landscape trajectories.” All of the images from both exhibitions were upscaled or downscaled while preserving the image’s aspect ratio (image sizes are shown in the upper part of each legend). The upscaling of the images did not change the topological properties significantly- for both groups, the markers occupy the same space. For downscaling, however, the area under the persistence landscape for both data sets is decreasing. It is important to note that at every level of resizing, the relative location of both datasets in the area area-under-landscape space was preserved- the artistic images have higher area-under-landscape than the pseudo-artistic images (except for 3 cases- image number 9 being significantly lower than any other image, and images 4 and 8 being very close to the pseudo-artistic images). It should be noted that for most of the images, downsizing by a factor of 4 (resulting in image size 512×359) did not change the area-under-landscape by less than one order of magnitude.(PDF)

S12 Fig Visualisation of all cycles in dimension 1, artistic images [11] with filtration from black to white colours. (PNG)

S13 Fig Visualisation of all cycles in dimension 1, pseudo-artistic images with filtration from black to white colours. (PNG)

S14 Fig Visualisation of all cycles in dimension 1, artistic images [11] with filtration from white to black colours. (PNG)

S15 Fig Visualisation of all cycles in dimension 1, pseudo-artistic images with filtration from white to black colours. (PNG)

S16 Fig Persistence barcodes, persistence landscapes, and Betti curves for the artistic image ‘Black wash’ [11], obtained by applying a filtrations (of a single colour) to each of the RGB channels separately. Each row corresponds to one colour channel: red (a), green (b), and blue (c). Left column: greyscale representation of the respective channel; middle column: topological features in dimension 0; right column: features in dimension 1.
(PNG)

S17 Fig Persistence barcodes, persistence landscapes, and Betti curves for the artistic image ‘Black hole’ [11], obtained by applying a filtrations (of a single colour) to each of the RGB channels separately. Each row corresponds to one colour channel: red (a), green (b), and blue (c). Left column: greyscale representation of the respective channel; middle column: topological features in dimension 0; right column: features in dimension 1.
(PNG)

S18 Fig Persistence barcodes, persistence landscapes, and Betti curves for the artistic image ‘The Inside’, obtained by applying a filtrations (of a single colour) to each of the RGB channels separately. Each row corresponds to one colour channel: red (a), green (b), and blue (c). Left column: greyscale representation of the respective channel; middle column: topological features in dimension 0; right column: features in dimension 1.
(PNG)

S19 Fig Persistence barcodes, persistence landscapes, and Betti curves for the pseudo-artistic image ‘Vibrations of time’, obtained by applying a filtrations (of a single colour) to each of the RGB channels separately. Each row corresponds to one colour channel: red (a), green (b), and blue (c). Left column: greyscale representation of the respective channel; middle column: topological features in dimension 0; right column: features in dimension 1.
(PNG)

S20 Fig Persistence barcodes, persistence landscapes, and Betti curves for the artistic image ‘Black wash’ [11], obtained by applying the reverse filtration to each of the RGB channels separately. Each row corresponds to one colour channel: red (a), green (b), and blue (c). Left column: greyscale representation of the respective channel; middle column: topological features in dimension 0; right column: features in dimension 1.
(PNG)

S21 Fig Persistence barcodes, persistence landscapes, and Betti curves for the artistic image ‘Black hole’ [11], obtained by applying the reverse filtration to each of the RGB channels separately. Each row corresponds to one colour channel: red (a), green (b), and blue (c). Left column: greyscale representation of the respective channel; middle column: topological features in dimension 0; right column: features in dimension 1.
(PNG)

S22 Fig Persistence barcodes, persistence landscapes, and Betti curves for the artistic image ‘The Inside’, obtained by applying the reverse filtration to each of the RGB channels separately. Each row corresponds to one colour channel: red (a), green (b), and blue (c). Left column: greyscale representation of the respective channel; middle column: topological features in dimension 0; right column: features in dimension 1.
(PNG)

S23 Fig Persistence barcodes, persistence landscapes, and Betti curves for the pseudo-artistic image ‘Vibrations of time’, obtained by applying the reverse filtration to each of the RGB channels separately. Each row corresponds to one colour channel: red (a), green (b), and blue (c). Left column: greyscale representation of the respective channel; middle column: topological features in dimension 0; right column: features in dimension 1.
(PNG)

S24 Fig Effect of histogram manipulation on the topological properties for artistic image. Different histogram transformations are indicated in the titles above the transformed images. Details about histogram manipulations are presented in S2 Table For each transformation, topological features are extracted for BW filtration and for each transformation, persistence landscapes, persistence barcodes, and Betti curves are shown for dimensions 0 and 1.
(PNG)

S25 Fig Effect of histogram manipulation on the pseudo-artistic image. Different histogram transformations are indicated in the titles above the transformed images. Details about histogram manipulations are presented in S2 Table. For each transformation, topological features are extracted for BW filtration and for each transformation, persistence landscapes, persistence barcodes, and Betti curves are shown for dimensions 0 and 1.
(PNG)

S26 Fig Effect of histogram manipulation on the artistic image, reverse filtration. Different histogram transformations are indicated in the titles above the transformed images. Details about histogram manipulations are presented in S2 Table. For each transformation, topological features are extracted for BW filtration and for each transformation, persistence landscapes, persistence barcodes, and Betti curves are shown for dimensions 0 and 1.
(PNG)

S27 Fig Effect of histogram manipulation on the pseudo-artistic image, reverse filtration. Different histogram transformations are indicated in the titles above the transformed images. Details about histogram manipulations are presented in S2 Table. For each transformation, topological features are extracted for BW filtration and for each transformation, persistence landscapes, persistence barcodes, and Betti curves are shown for dimensions 0 and 1.
(PNG)

S28 Fig Comparison of *L*^1^ distance matrices for image transformations- contrast stretching. All presented results are for filtration from black to white. Each heatmap displays *L*^1^ pairwise differences between the persistence landscapes of all images in dimensions 0 (left) and 1 (right). Every row (or column) in the heatmap corresponds to an image, annotated between the matrices, to which a transformation was applied as indicated by the titles. The top 12 labels (coloured orange) are from pseudo-artistic images, and the next 12 labels (coloured green) are from artistic images. The overall shape differences captured with the *L*^1^ distance between landscapes across transformation are preserved. The relation between images is preserved, that is, in all cases, the distance between pseudo-artistic images was lower than for art images. More details about how the distance is computed is presented in S10 Fig.
(PNG)

S29 Fig Comparison of *L*^1^ distance matrices for different image transformations, linear stretching and gamma correction. All presented results are for filtration from black to white. Each heatmap displays *L*^1^ pairwise differences between the persistence landscapes of all images in dimensions 0 (left) and 1 (right). Every row (or column) in the heatmap corresponds to an image, annotated between the matrices, to which a transformation was applied as indicated by the titles. The top 12 labels (coloured orange) are from pseudo-artistic images, and the next 12 labels (coloured green) are from artistic images. The overall shape differences captured with the *L*^1^ distance between landscapes across transformation are preserved. More details about how the distance is computed is presented in S10 Fig.
(PNG)

S30 FigCycle density intrinsic ECDF curves, $ECDF(M_{density},U)$.The ECDFs for artistic images are marked green and the pseudo-artistic images orange for different window sizes (plots from top to bottom): 21, 51, 101, 151, 201, 401.(PDF)

S31 FigIntrinsic ECDF curves for cycle density feature map.$ECDF(M_{density},U)$, for: BW filtration (top row), WB filtration (middle row), combined BW and WB filtration (bottom row).(PDF)

S32 FigECDF curves for maximal persistence feature map.$ECDF(M_{persistence},U)$, for: BW filtration (top row), WB filtration (middle row), combined BW and WB filtration (bottom row).(PDF)

S33 FigIntrinsic ECDF curves for cycle perimeter feature map.$ECDF(M_{perimeter},U)$, for: BW filtration (top row), WB filtration (middle row), combined BW and WB filtration (bottom row).(PDF)

S34 FigECDFs for maximal persistence for each image and each participant.(a) data shown for art [11]. (b) data shown for pseudo art. Both sessions. Within a column the plots are (from left to right): scatterplot of the Kolmogorov-Smirnov statistic between the image’s ECDF vs ‘looking’ ECDF and images’s ECDF vs ‘not looking’ ECDF for each person; bar plot of the same; the ‘Looking ECDF’ for each person; ‘Not looking ECDF’ for each person; the image itself. The blue ECDF is intrinsic to the image itself and can be thought to be arising from a ‘gaze’ that is a uniform scan of the entire image. Results for the full set of ECDFs. For each image *k*, feature map *M* and participant *s*, we have $ECDF(M_{k},U)$ (‘intrinsic’), $ECDF(M_{k},G_{s})$ (where the participant was ‘looking’), $ECDF(M_{k},\tilde{G_{s}})$ (where the participant was ‘not looking’).(PDF)

S35 Fig ECDFs for cycle perimeter for each image and each participant. (a) data shown for art [11]. (b) data shown for pseudo art. Both sessions. Within a column, the plots are (from left to right): scatterplot of the Kolmogorov-Smirnov statistic between the image’s ECDF vs ‘looking’ ECDF and images’s ECDF vs ‘not looking’ ECDF for each person; bar plot of the same; the ‘Looking ECDF’ for each person; ‘Not looking ECDF’ for each person; the image itself. The blue ECDF is intrinsic to the image itself and can be thought to be arising from a ‘gaze’ that is a uniform scan of the entire image.
(PDF)

S36 Fig ECDFs for cycle density for each image and each participant. (a) data shown for art [11]. (b) data shown for pseudo art. Both sessions. Within a column, the plots are (from left to right): scatterplot of the Kolmogorov-Smirnov statistic between the image’s ECDF vs ‘looking’ ECDF and images’s ECDF vs ‘not looking’ ECDF for each person; bar plot of the same; the ‘Looking ECDF’ for each person; ‘Not looking ECDF’ for each person; the image itself. The blue ECDF is intrinsic to the image itself and can be thought to be arising from a ‘gaze’ that is a uniform scan of the entire image.
(PDF)

S1 Appendix EEG study. (PDF)

S2 Appendix Aesthetic Experience Questionnaires. (PDF)

S3 Appendix Persistent Homology Here we provide more details and definitions for Persistent Homology, Cubical Complexes, Betti curves and Persistence Landscapes. We also discuss the proof of Alexander duality in more detail.
(PDF)

S4 Appendix Heatmaps, feature maps and operations on ECDF. (PDF)

S5 Appendix The effect of image resizing and histogram manipulations. (PDF)

S6 Appendix Titles of artworks by Richter, Kandinsky, Rothko, Malevich, Jarema, and Pollock. (PDF)

S1 Table Feature maps, where *F* ∈ {*BW,WB*} is the filtration. (PDF)

S2 Table Overview of image contrast adjustment transformations, tested parameter configurations, and their short descriptions [90]. (PDF)

S3 Table Statistics for Alexander duality violation distributions. (PDF)
