# Supplementary figures and images for: Art’s hidden topology: A window into human perception
Source: PLoS Comput Biol. 2026 May 14;22(5):e1014156. doi: 10.1371/journal.pcbi.1014156 (PMC13175340; doi:10.1371/journal.pcbi.1014156)

# Beta waves

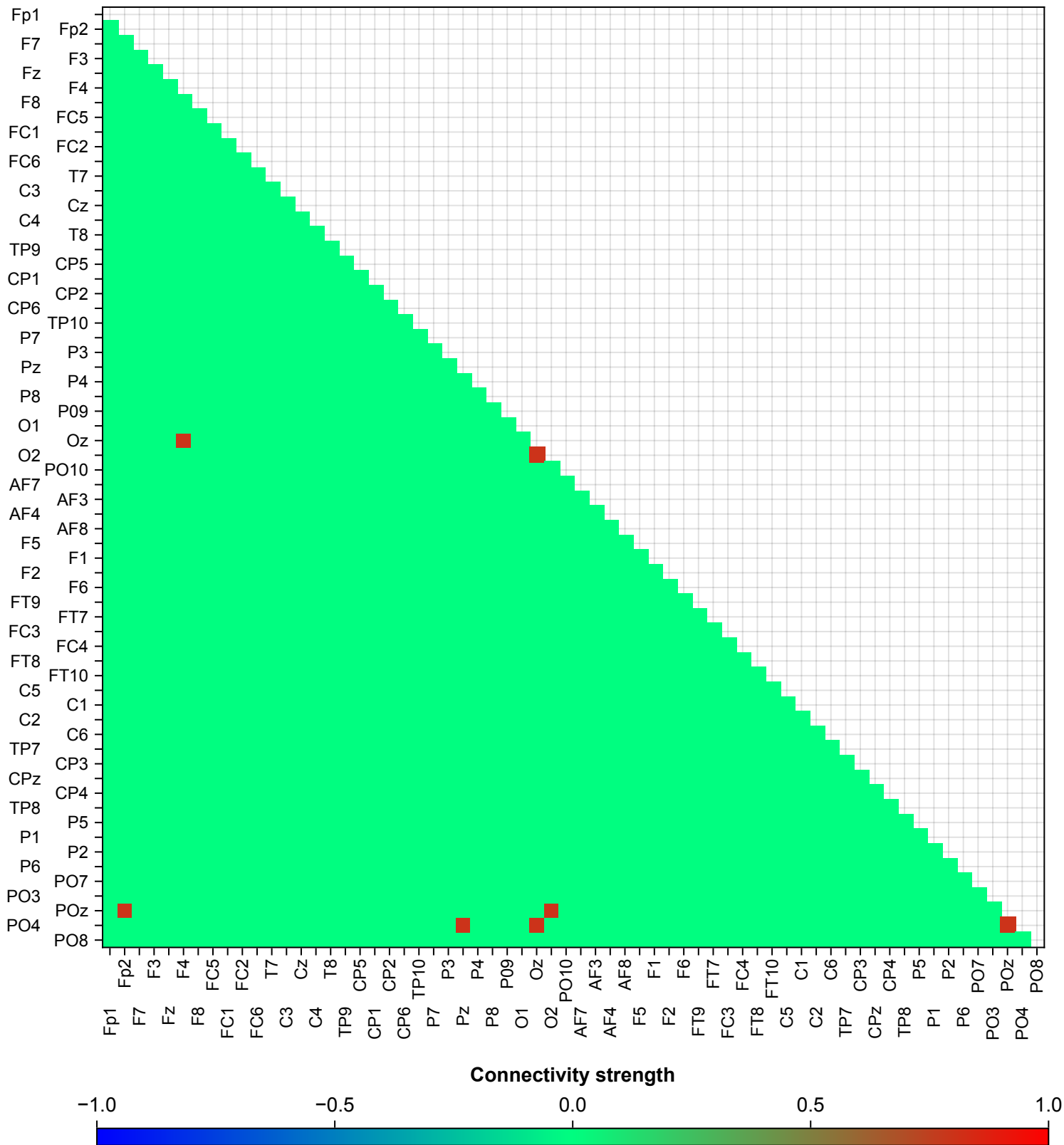

Supplement: S1 Fig — A red colour denotes higher connectivity strength in the artistic group, blue in the pseudo-artistic group. All differences are significant at p < 0.05 (FDR corrected). (PDF) [file pcbi.1014156.s001.pdf]

# Gamma waves

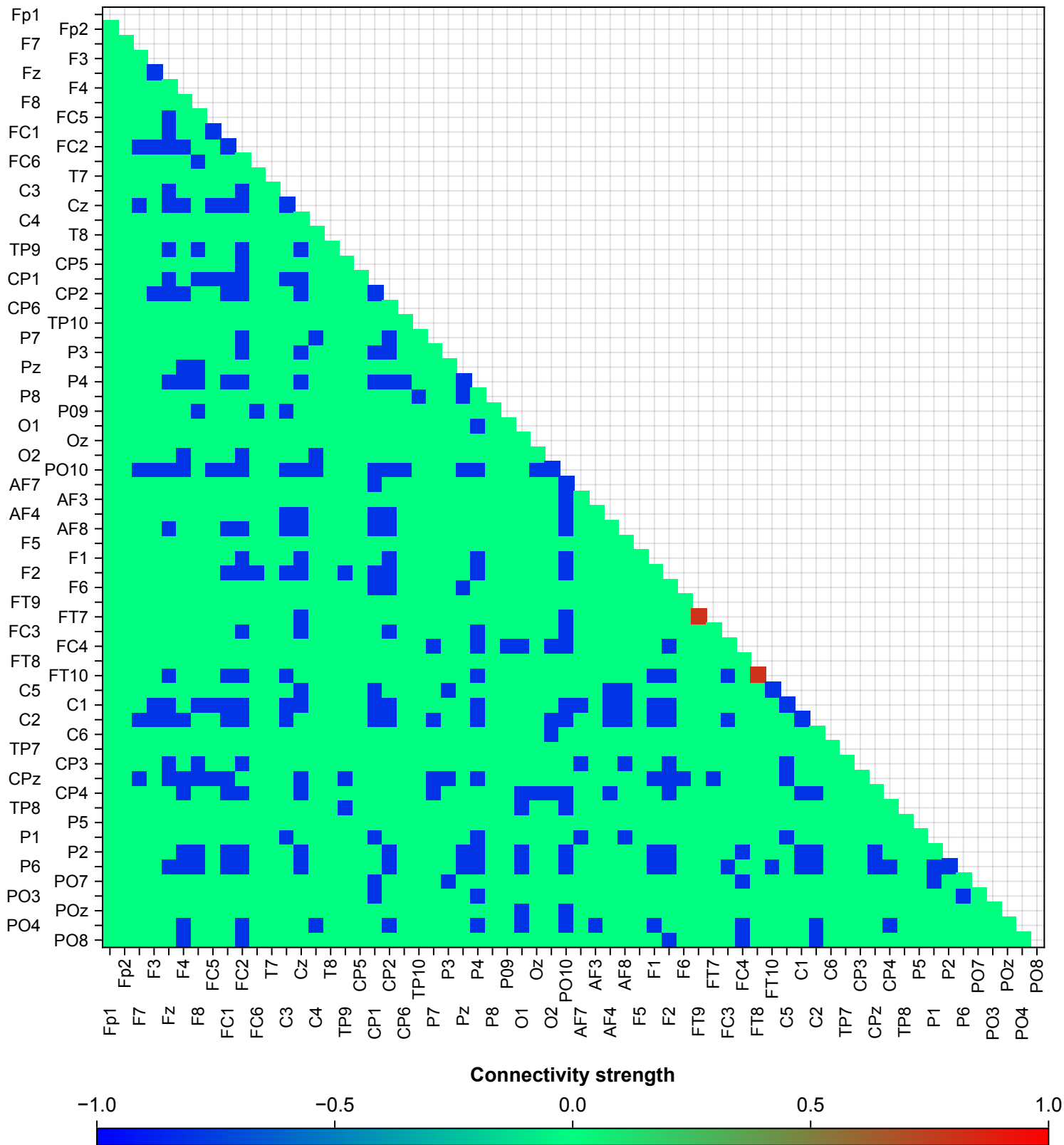

Supplement: S2 Fig — A red colour denotes higher connectivity strength in the artistic group, blue in the pseudo-artistic group. All differences are significant at p < 0.05 (FDR corrected). (PDF) [file pcbi.1014156.s002.pdf]

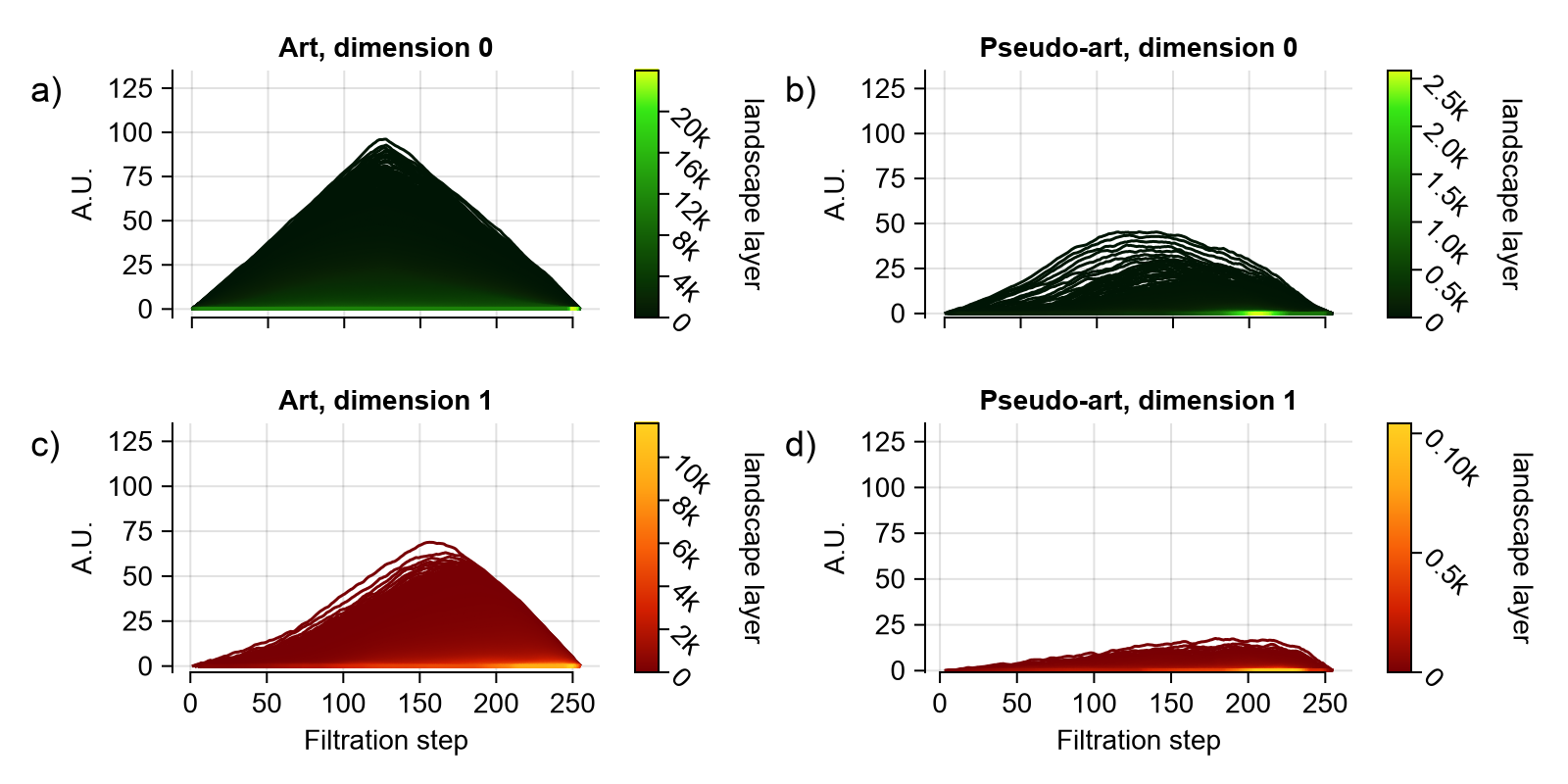

Supplement: S7 Fig — The average persistence landscape was computed for each group: Art (a,c) and pseudo-art images (b,d), with results for dimensions 0 shown in the top row and dimension 1 in the bottom row. Every individual landscape was constructed from cycles of persistence greater than 5 pixel intensity values. (PNG) [file pcbi.1014156.s007.png]

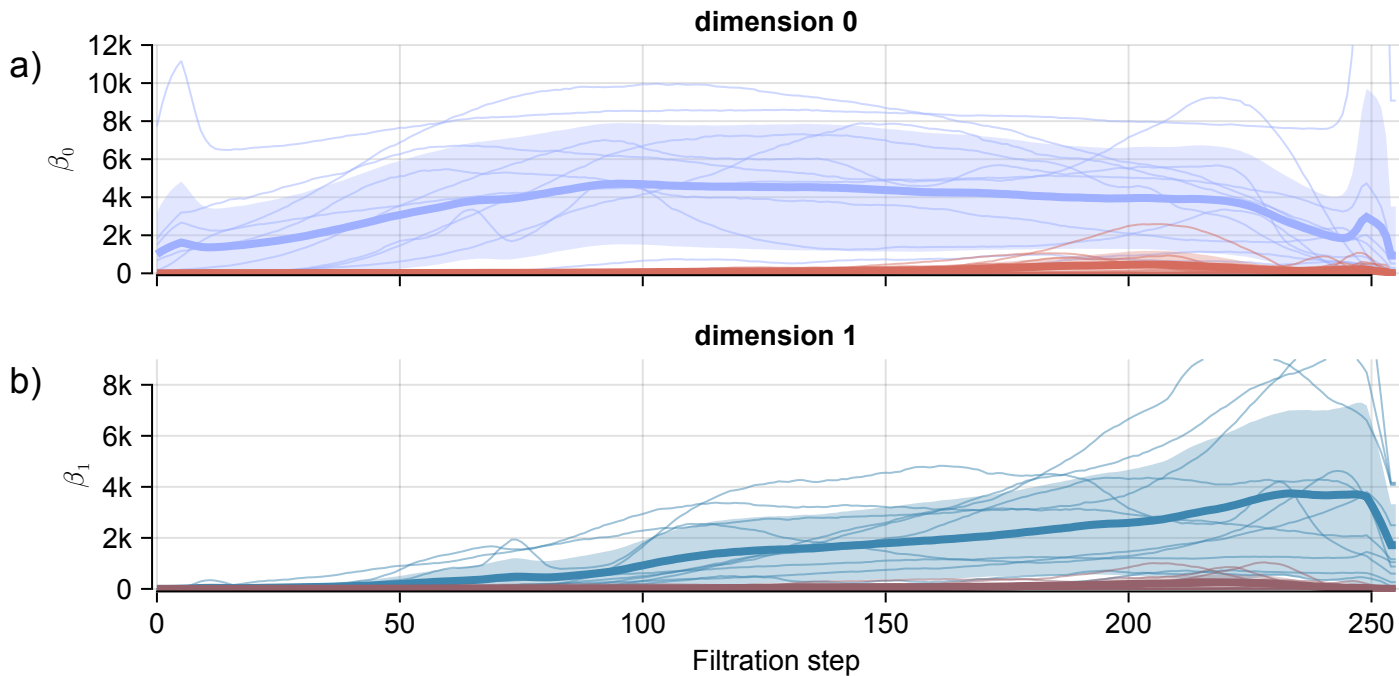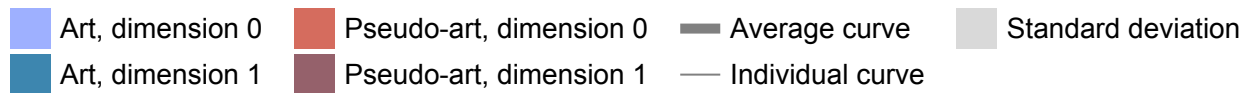

Supplement: S8 Fig — The average Betti curve was computed for each group: Art and pseudo-art images, with results for dimensions 0 (a) and dimension 1 (b). (PDF) [file pcbi.1014156.s008.pdf]

a) Art

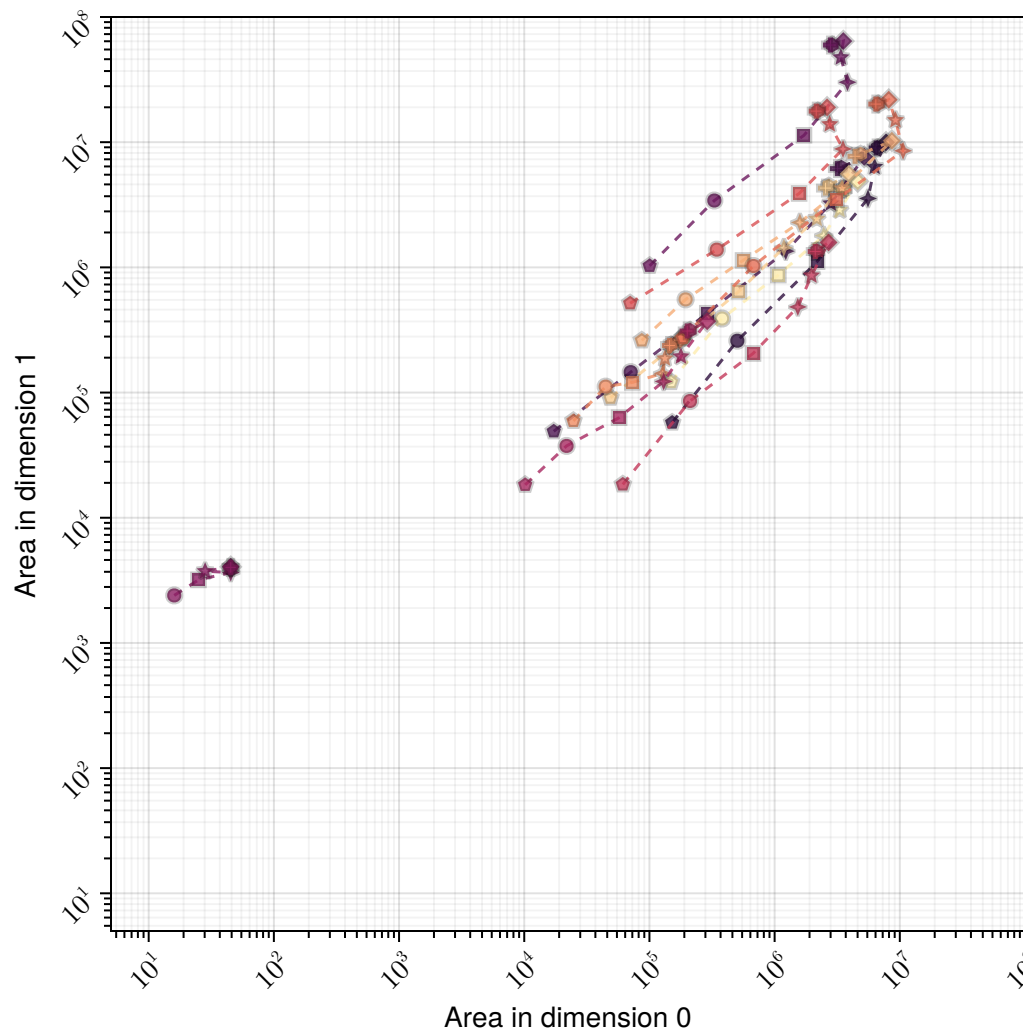

b) Pseudo-art

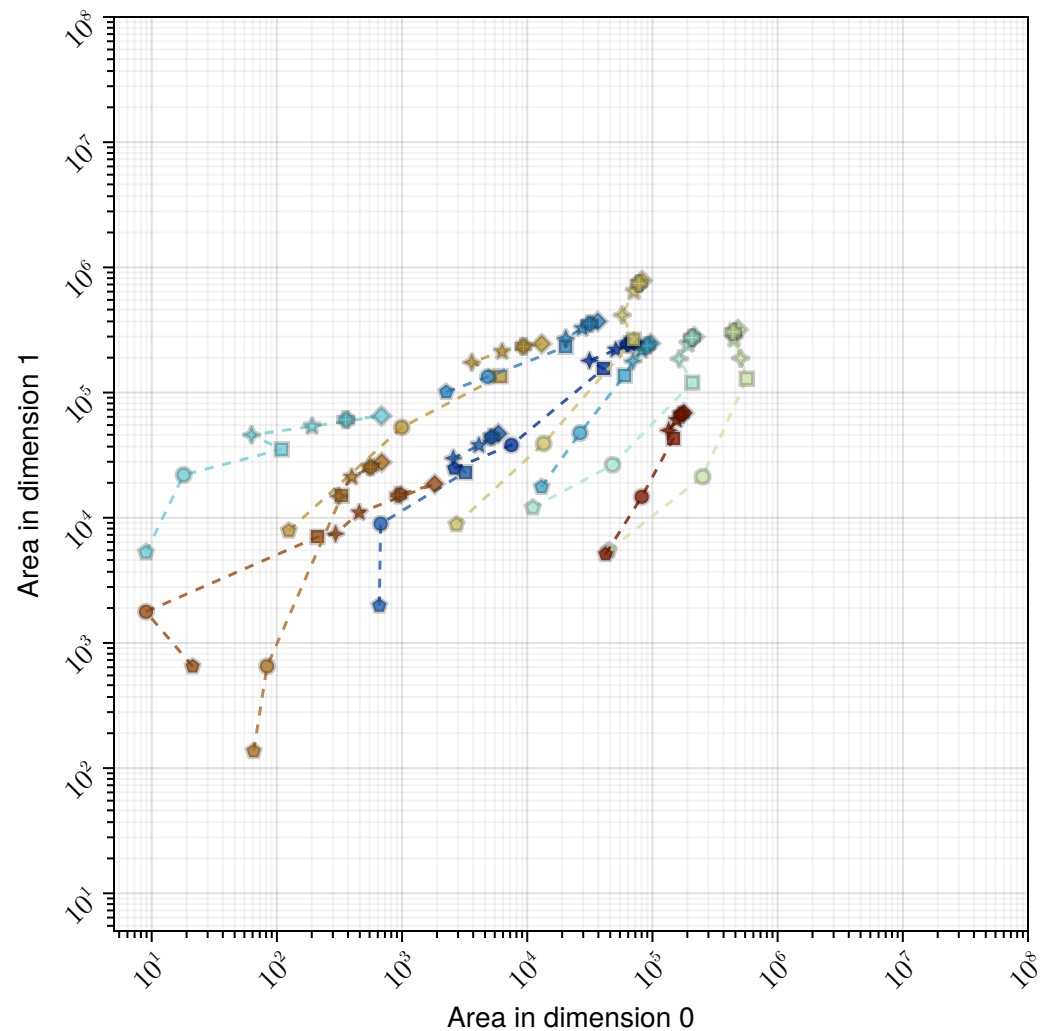

## Image size

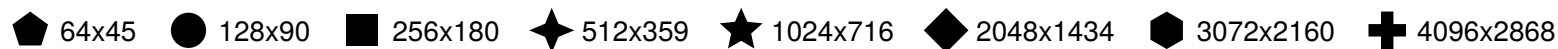

## Art images

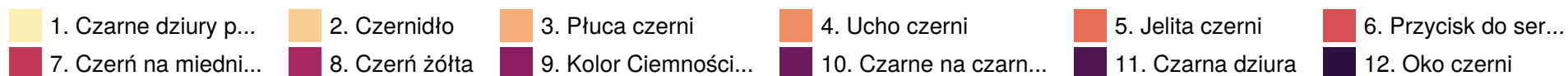

## Pseudo-art images

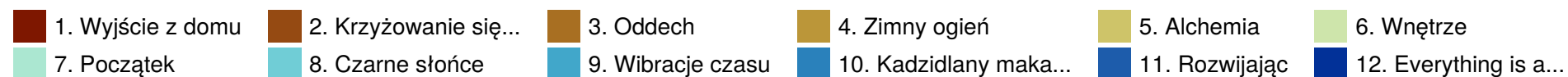

Supplement: S11 Fig — The horizontal and vertical coordinates are areas under the landscape in dimensions 0 and 1, respectively. A: results for artistic images. B: results for pseudo-artistic images. Persistence properties were computed for each image (marked with different colours) after resizing (size indicated by markers), creating “landscape trajectories.” All of the images from both exhibitions were upscaled or downscaled while preserving the image’s aspect ratio (image sizes are shown in the upper part of each legend). The upscaling of the images did not change the topological properties significantly- for both groups, the markers occupy the same space. For downscaling, however, the area under the persistence landscape for both data sets is decreasing. It is important to note that at every level of resizing, the relative location of both datasets in the area area-under-landscape space was preserved- the artistic images have higher area-under-landscape than the pseudo-artistic images (except for 3 cases- image number 9 being significantly lower than any other image, and images 4 and 8 being very close to the pseudo-artistic images). It should be noted that for most of the images, downsizing by a factor of 4 (resulting in image size 512×359) did not change the area-under-landscape by less than one order of magnitude. (PDF) [file pcbi.1014156.s011.pdf]

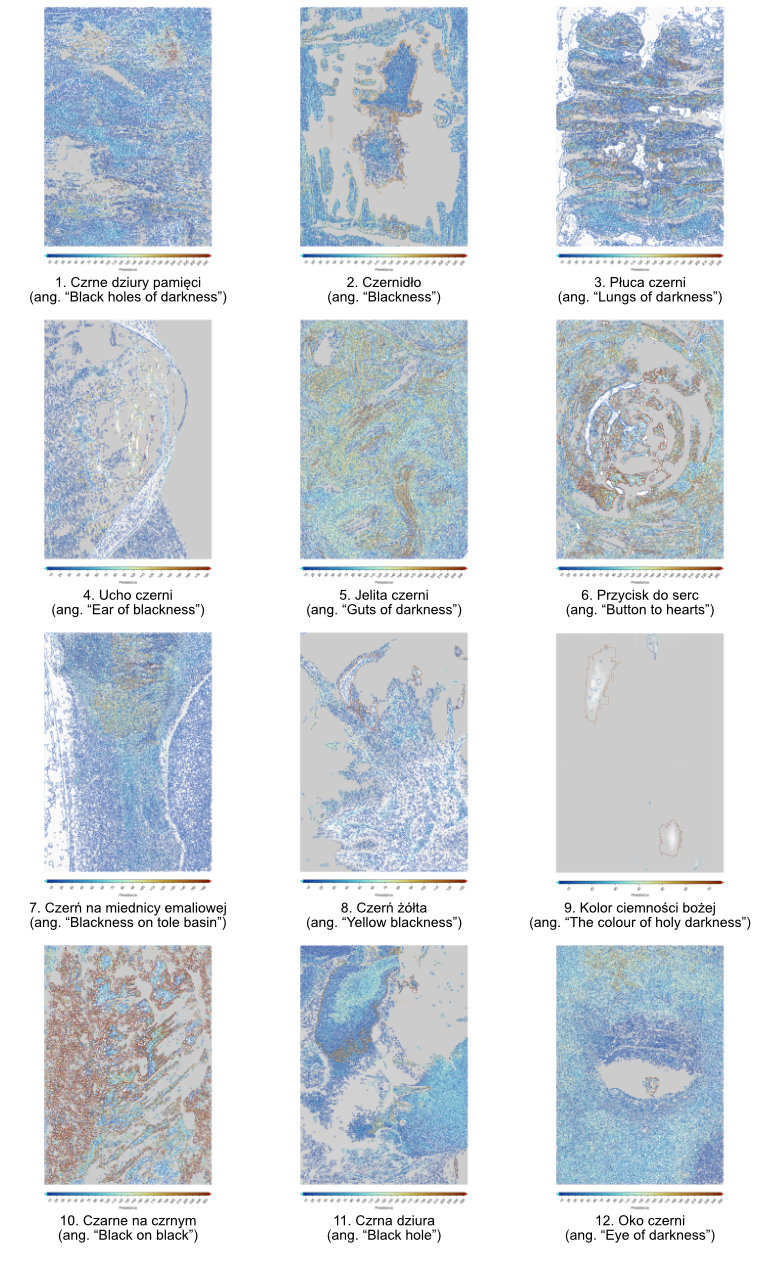

Supplement: S12 Fig — (PNG) [file pcbi.1014156.s012.png]

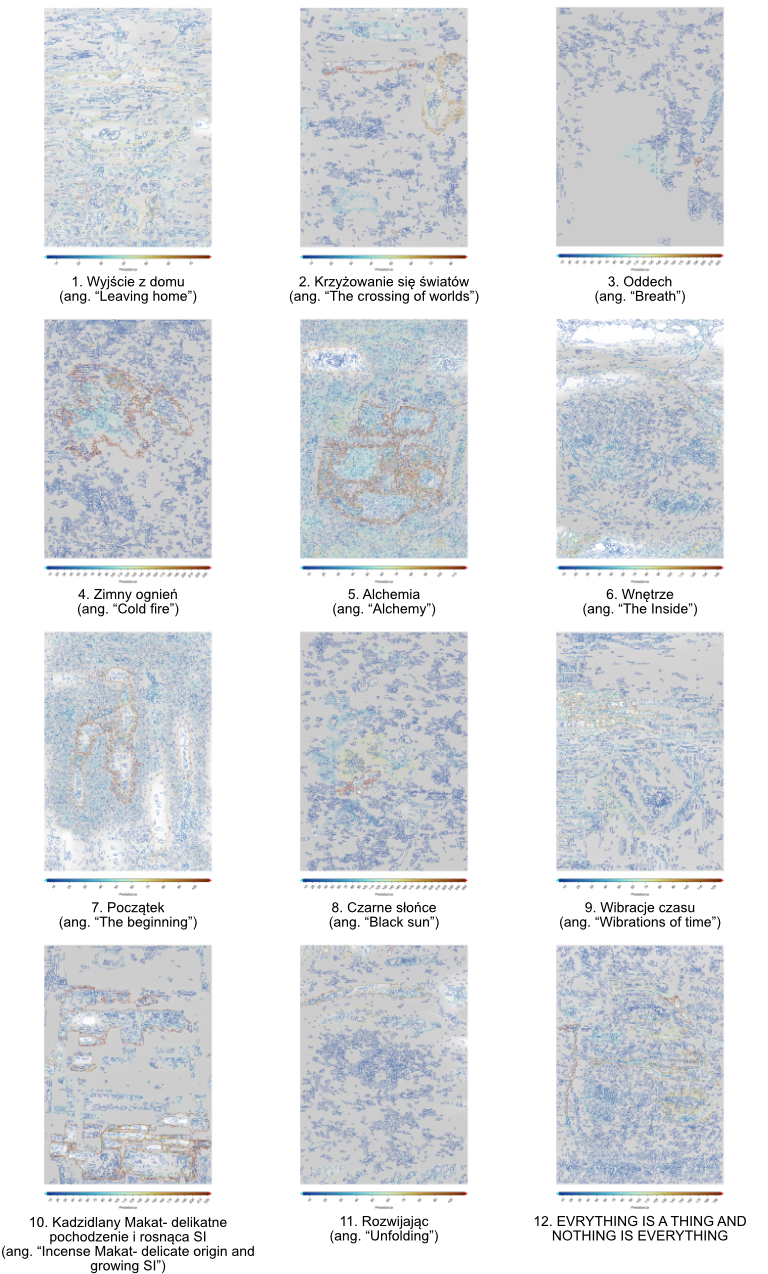

Supplement: S13 Fig — (PNG) [file pcbi.1014156.s013.png]

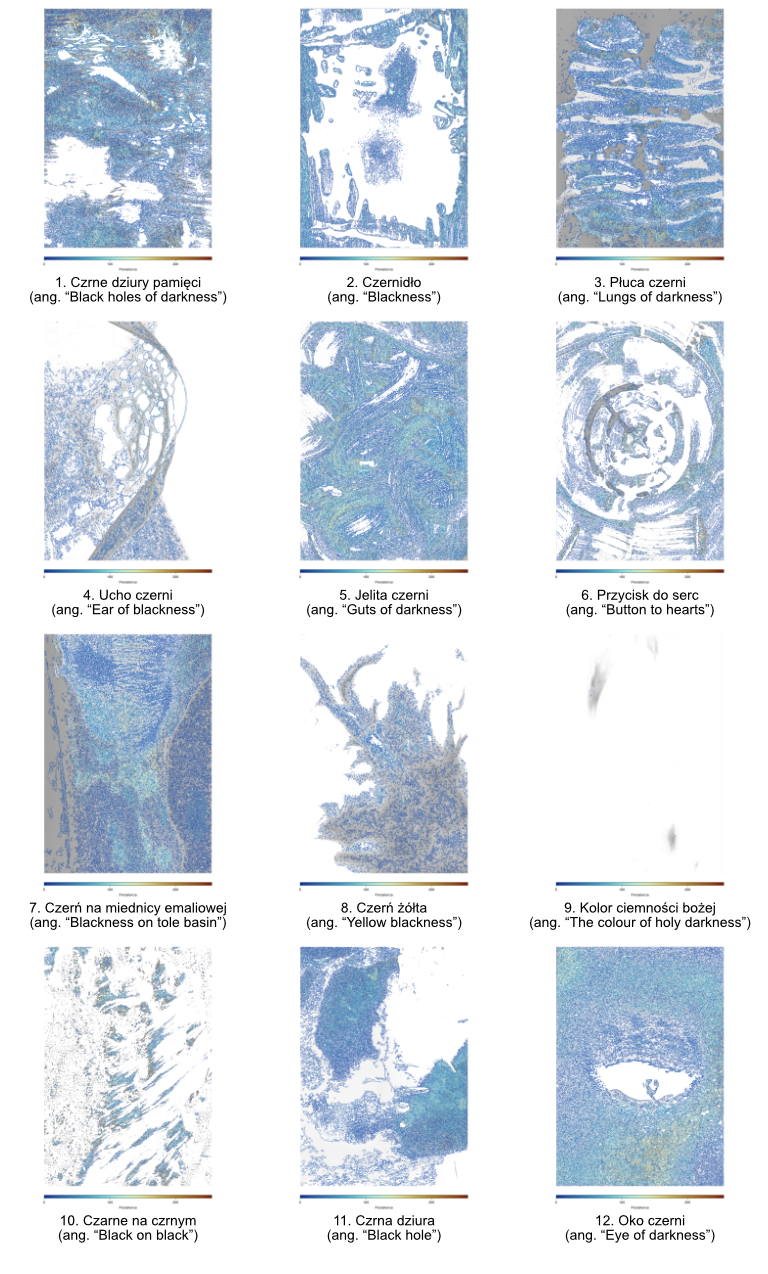

Supplement: S14 Fig — (PNG) [file pcbi.1014156.s014.png]

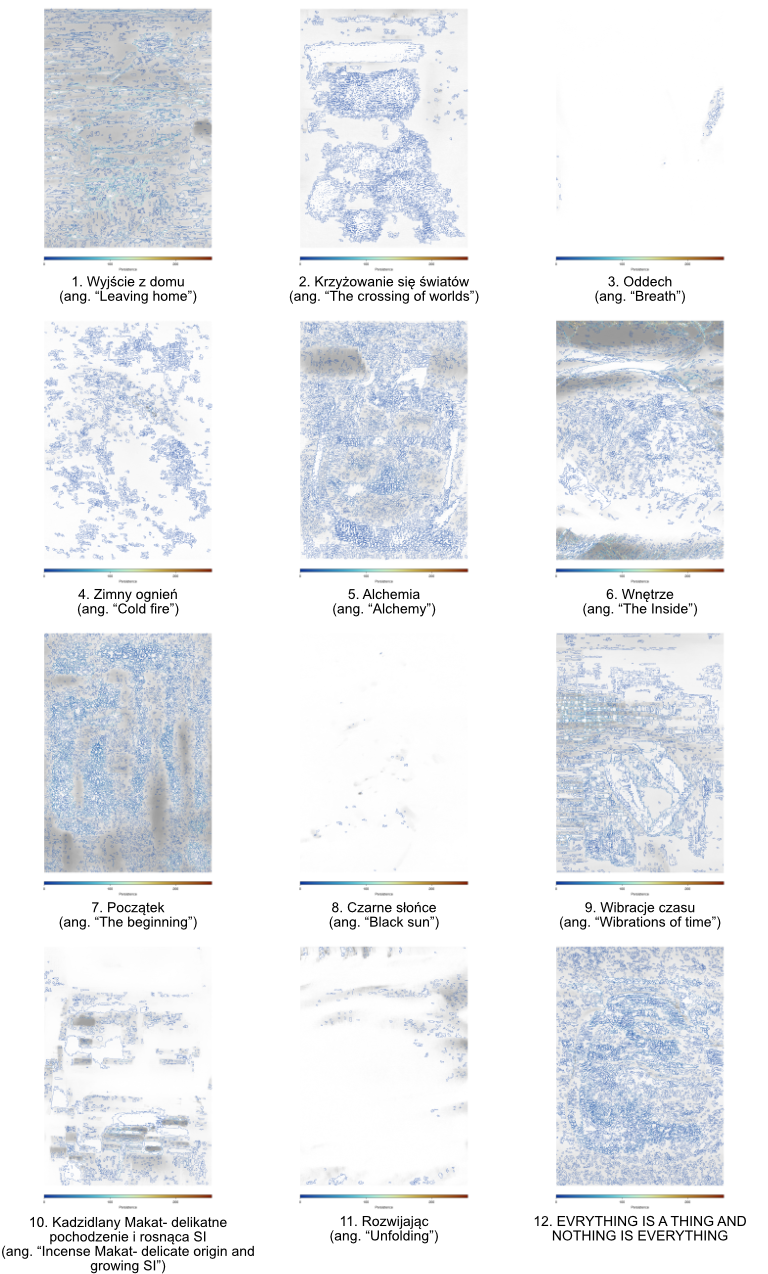

Supplement: S15 Fig — (PNG) [file pcbi.1014156.s015.png]

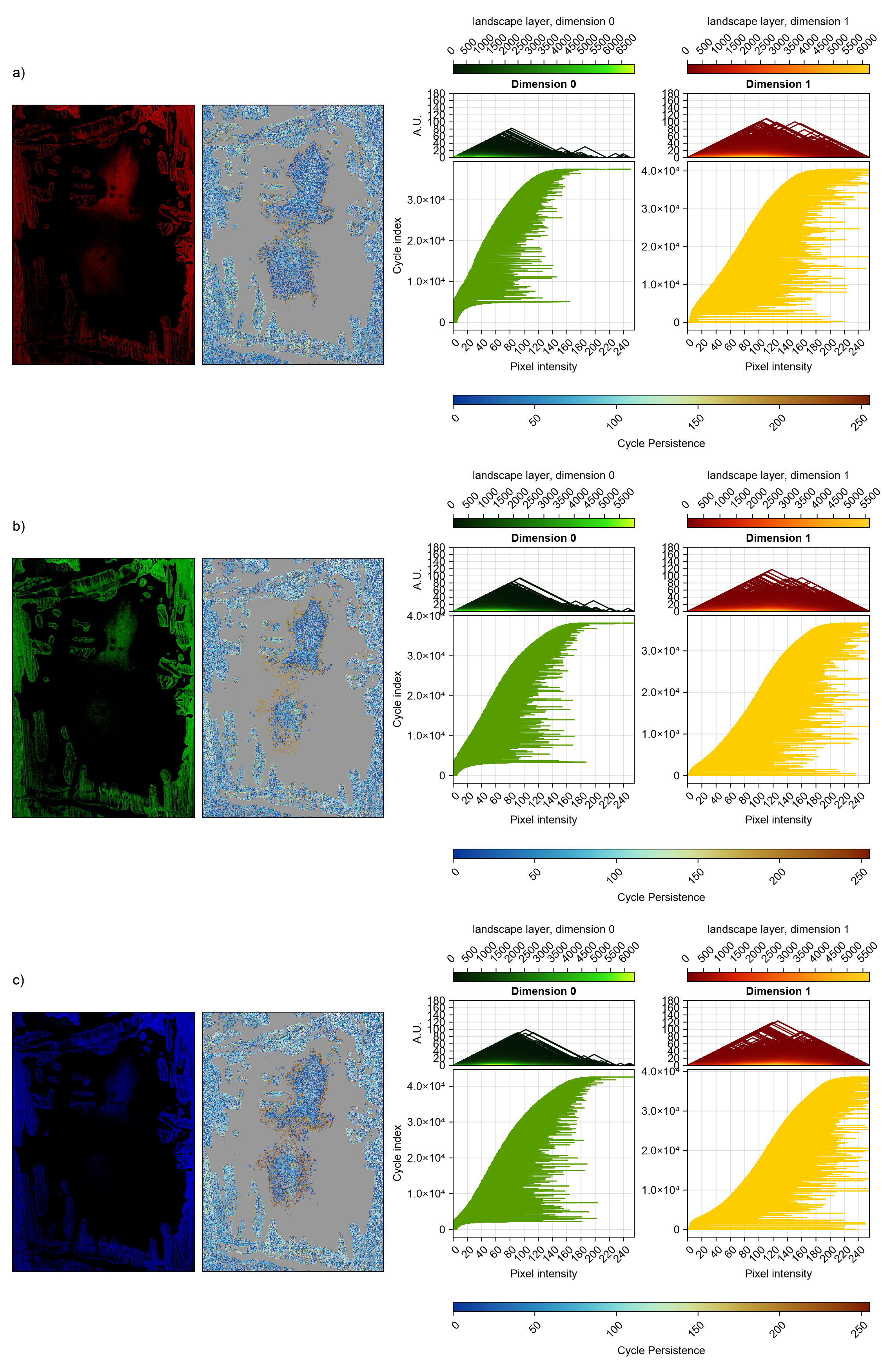

Supplement: S16 Fig — Each row corresponds to one colour channel: red (a), green (b), and blue (c). Left column: greyscale representation of the respective channel; middle column: topological features in dimension 0; right column: features in dimension 1. (PNG) [file pcbi.1014156.s016.png]

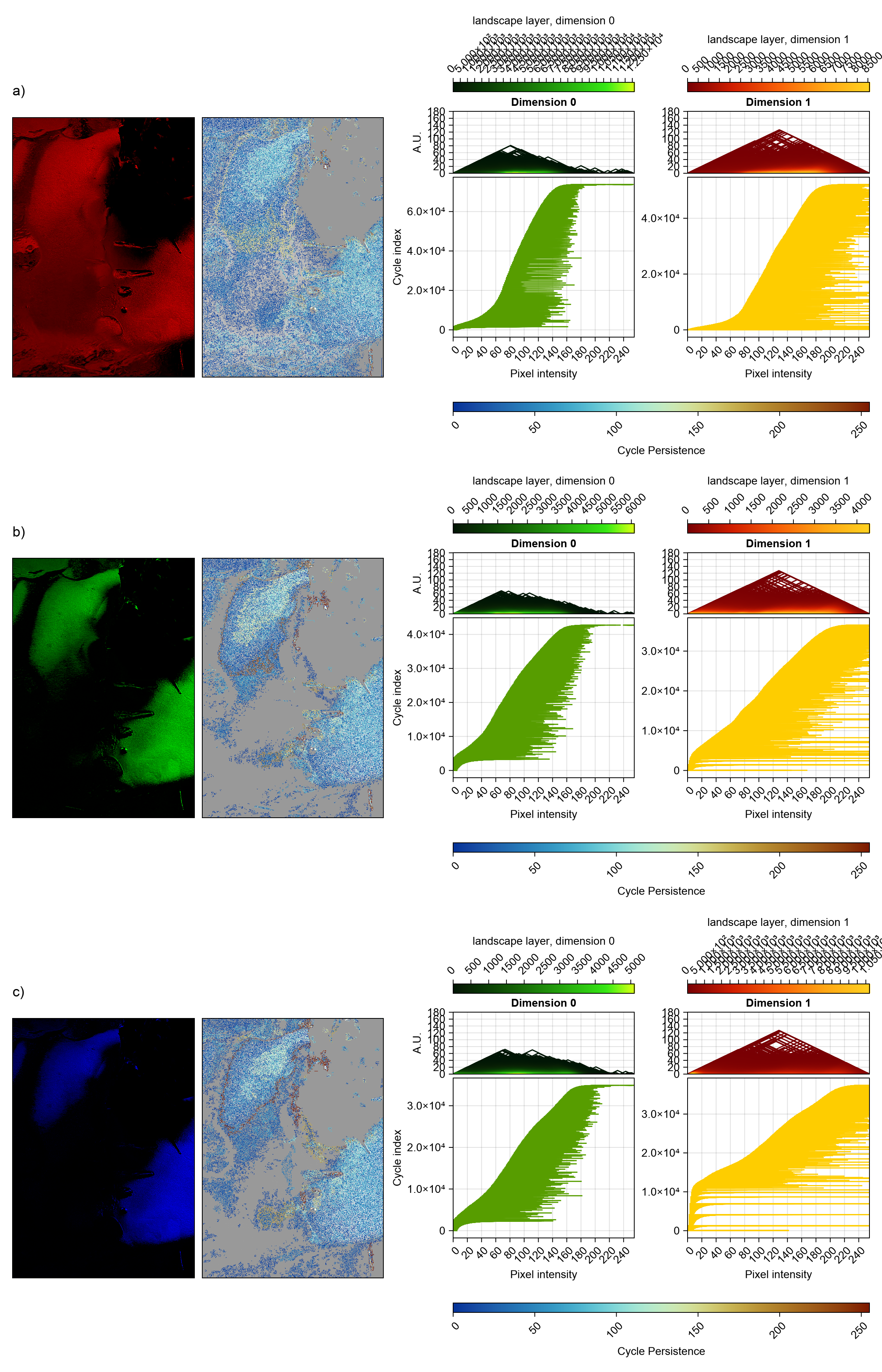

Supplement: S17 Fig — Each row corresponds to one colour channel: red (a), green (b), and blue (c). Left column: greyscale representation of the respective channel; middle column: topological features in dimension 0; right column: features in dimension 1. (PNG) [file pcbi.1014156.s017.png]

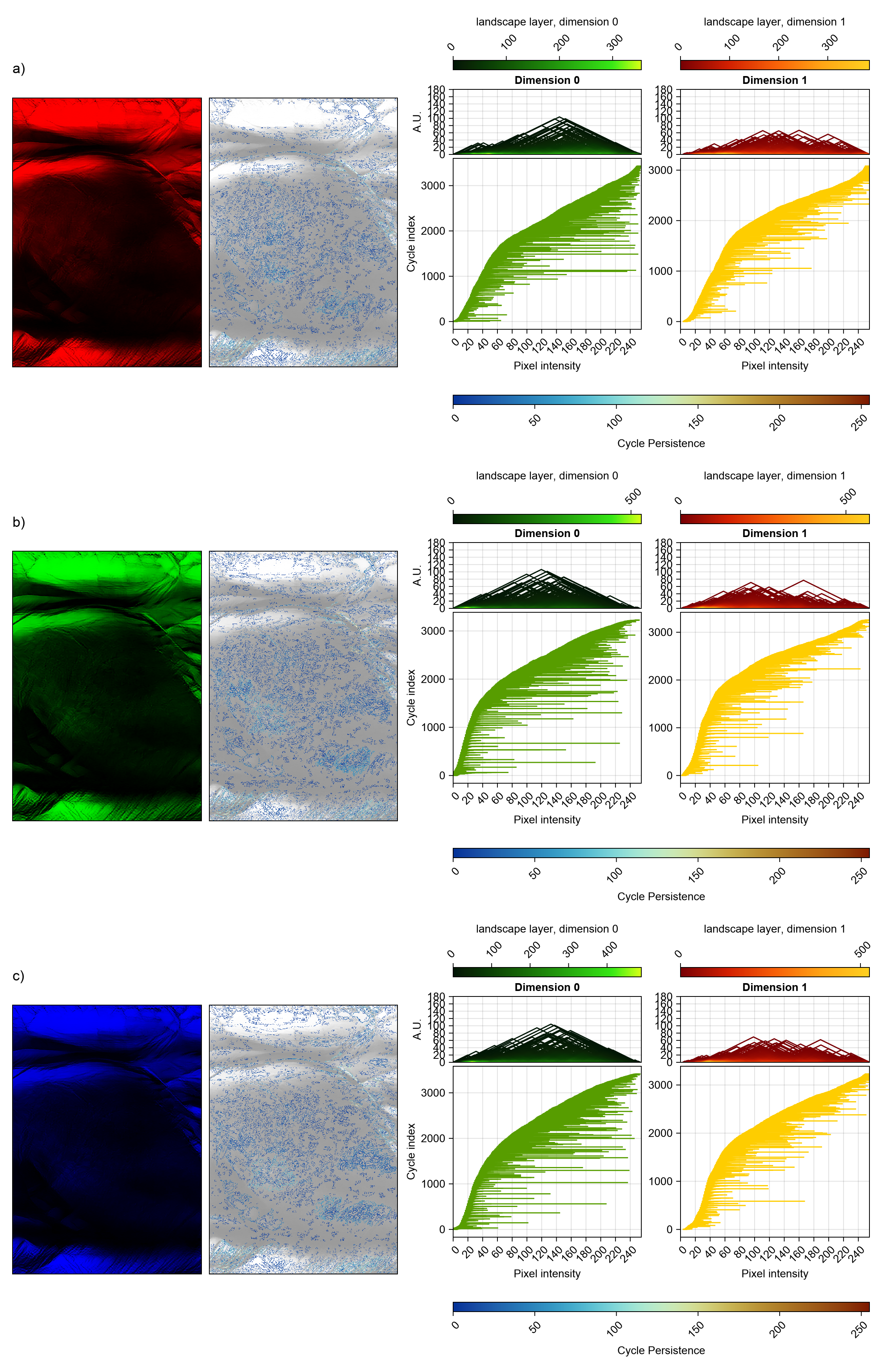

Supplement: S18 Fig — Each row corresponds to one colour channel: red (a), green (b), and blue (c). Left column: greyscale representation of the respective channel; middle column: topological features in dimension 0; right column: features in dimension 1. (PNG) [file pcbi.1014156.s018.png]

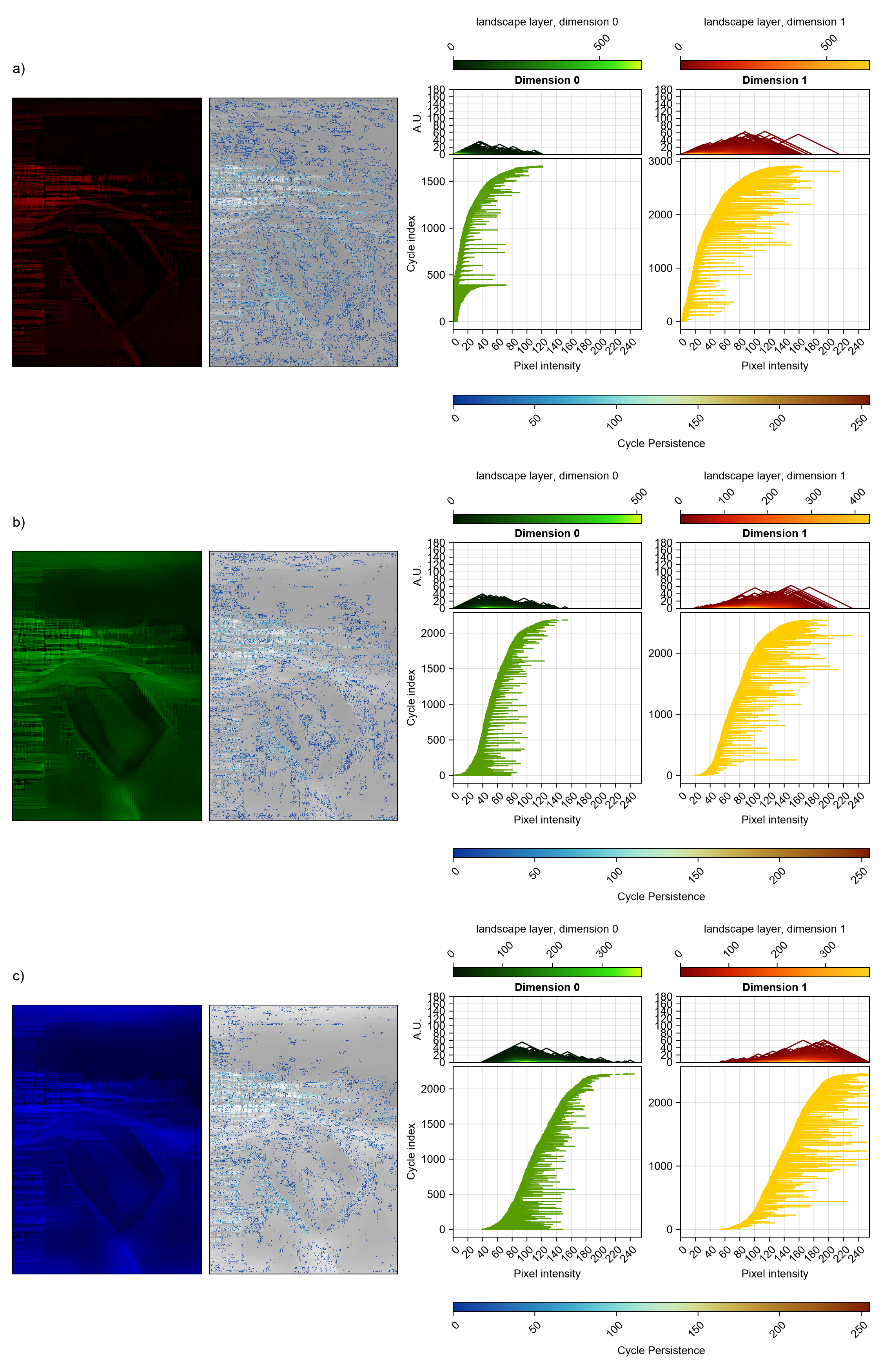

Supplement: S19 Fig — Each row corresponds to one colour channel: red (a), green (b), and blue (c). Left column: greyscale representation of the respective channel; middle column: topological features in dimension 0; right column: features in dimension 1. (PNG) [file pcbi.1014156.s019.png]

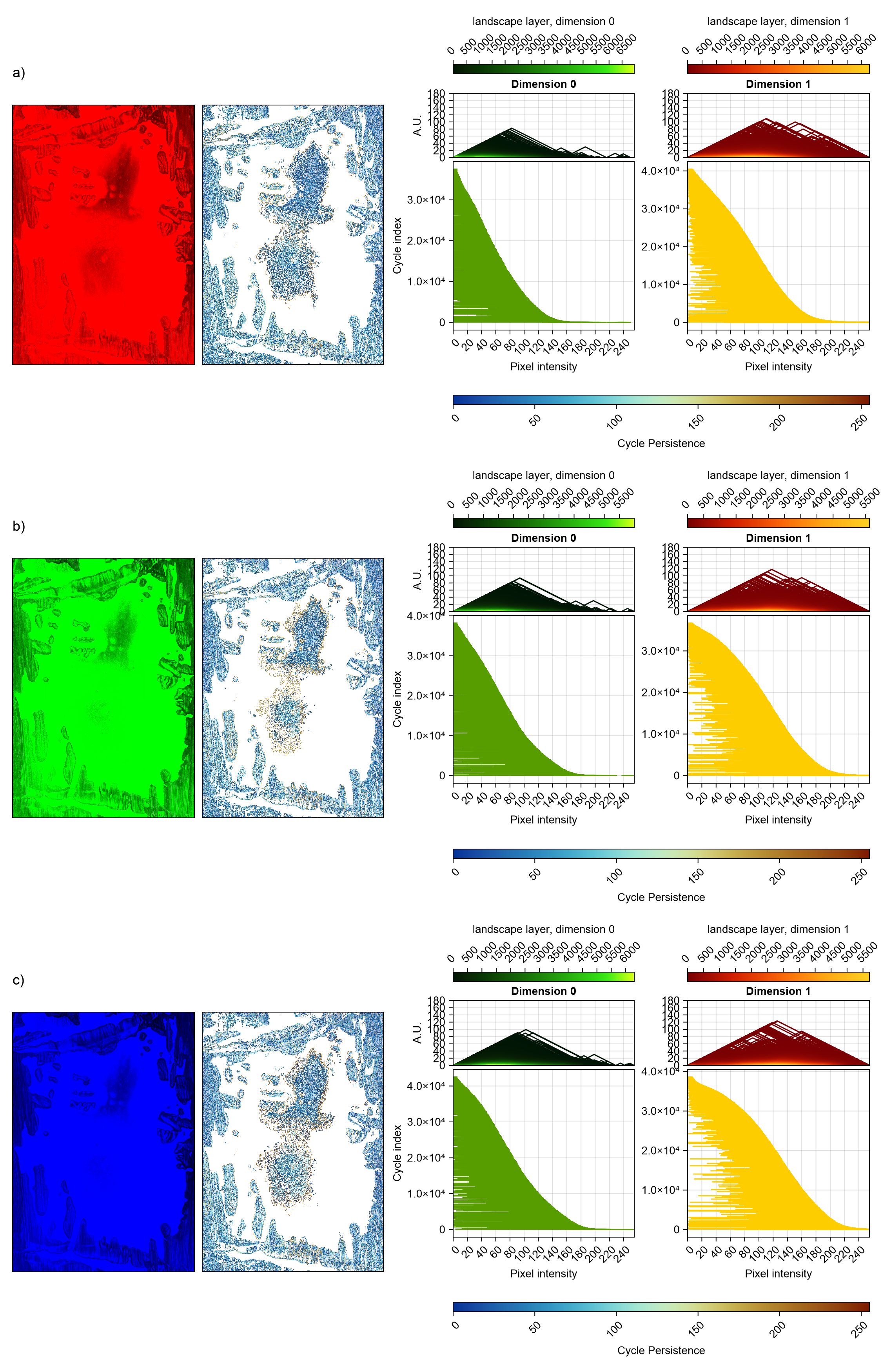

Supplement: S20 Fig — Each row corresponds to one colour channel: red (a), green (b), and blue (c). Left column: greyscale representation of the respective channel; middle column: topological features in dimension 0; right column: features in dimension 1. (PNG) [file pcbi.1014156.s020.png]

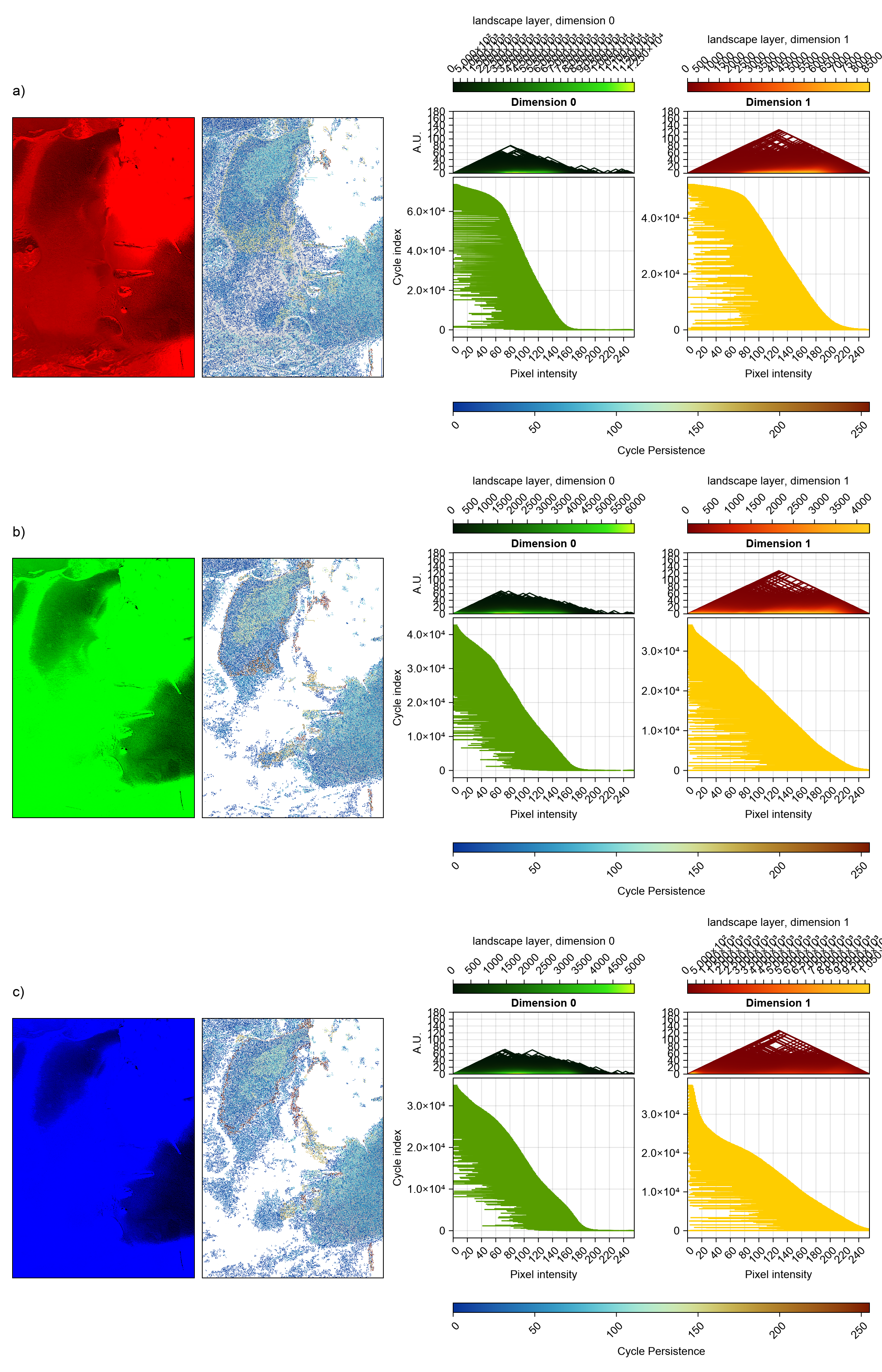

Supplement: S21 Fig — Each row corresponds to one colour channel: red (a), green (b), and blue (c). Left column: greyscale representation of the respective channel; middle column: topological features in dimension 0; right column: features in dimension 1. (PNG) [file pcbi.1014156.s021.png]

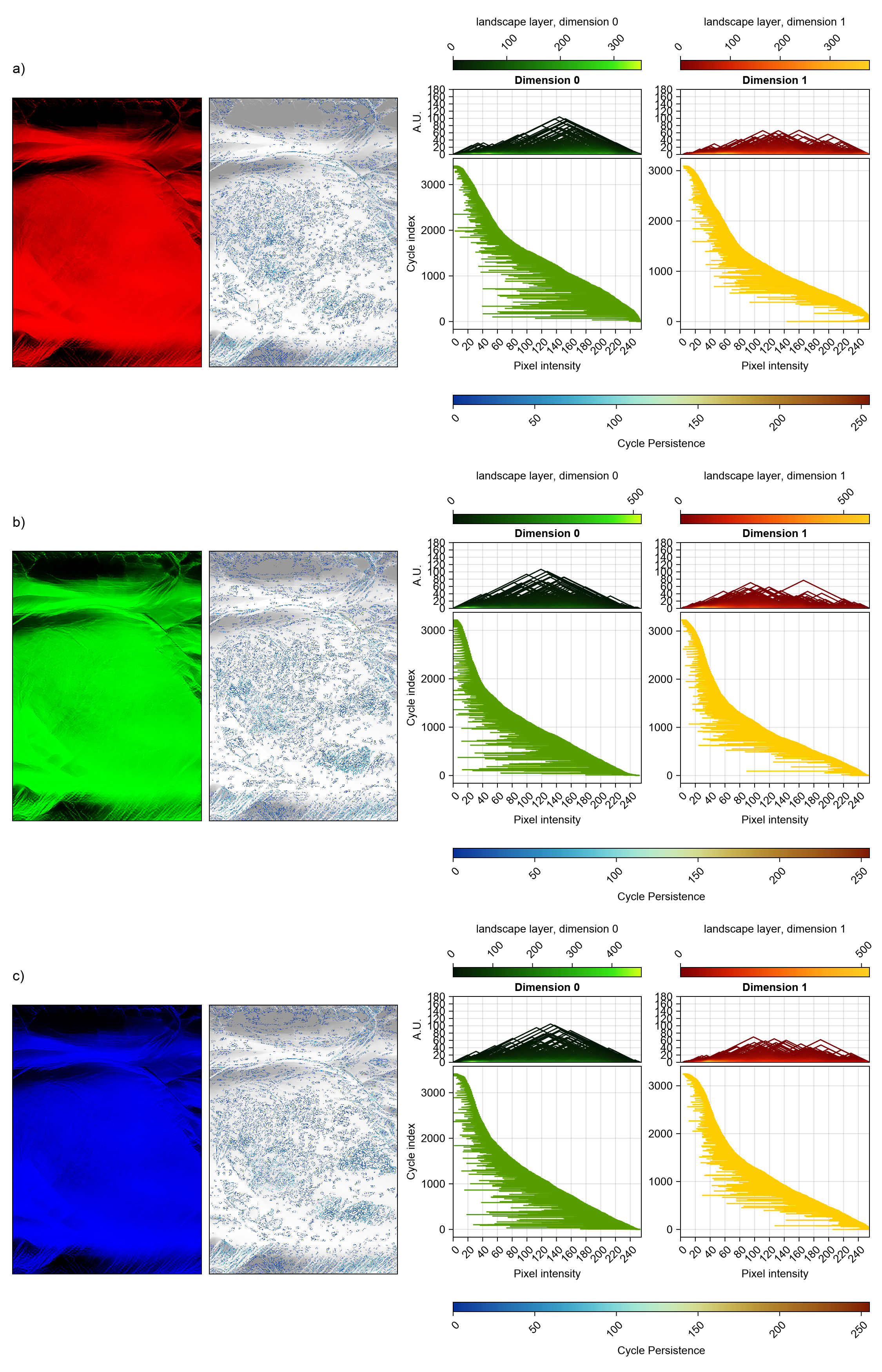

Supplement: S22 Fig — Each row corresponds to one colour channel: red (a), green (b), and blue (c). Left column: greyscale representation of the respective channel; middle column: topological features in dimension 0; right column: features in dimension 1. (PNG) [file pcbi.1014156.s022.png]

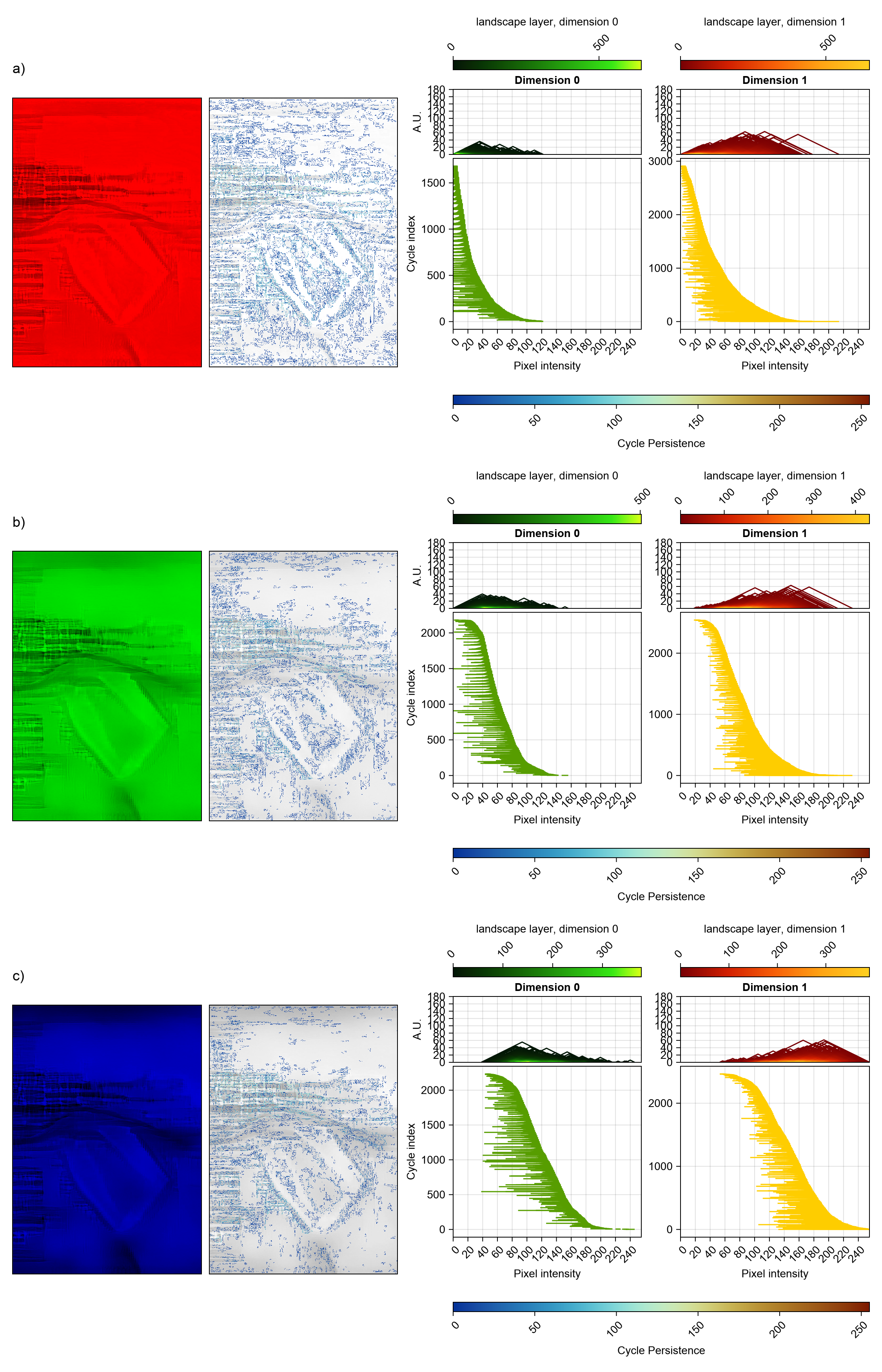

Supplement: S23 Fig — Each row corresponds to one colour channel: red (a), green (b), and blue (c). Left column: greyscale representation of the respective channel; middle column: topological features in dimension 0; right column: features in dimension 1. (PNG) [file pcbi.1014156.s023.png]

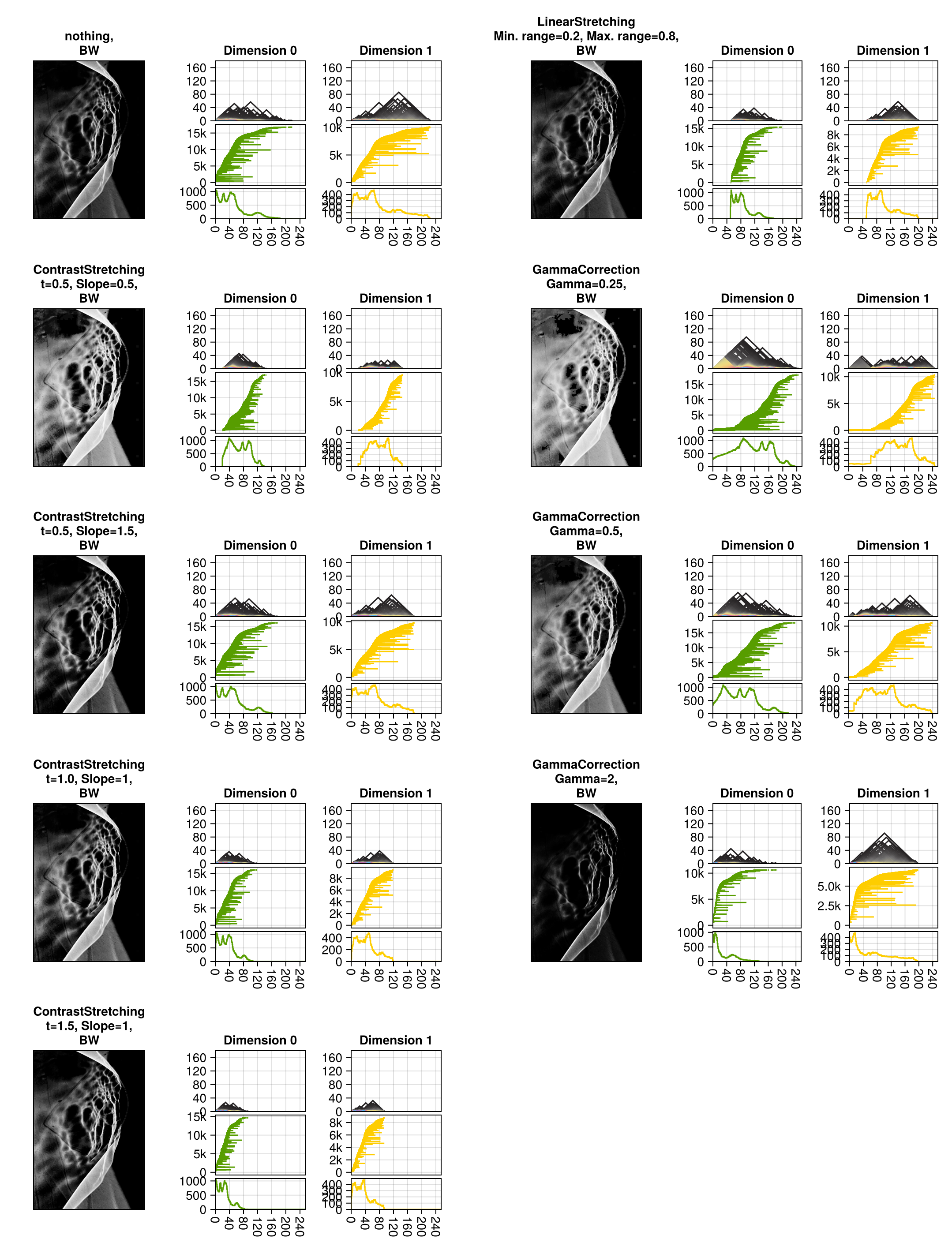

Supplement: S24 Fig — Different histogram transformations are indicated in the titles above the transformed images. Details about histogram manipulations are presented in S2 Table For each transformation, topological features are extracted for BW filtration and for each transformation, persistence landscapes, persistence barcodes, and Betti curves are shown for dimensions 0 and 1. (PNG) [file pcbi.1014156.s024.png]

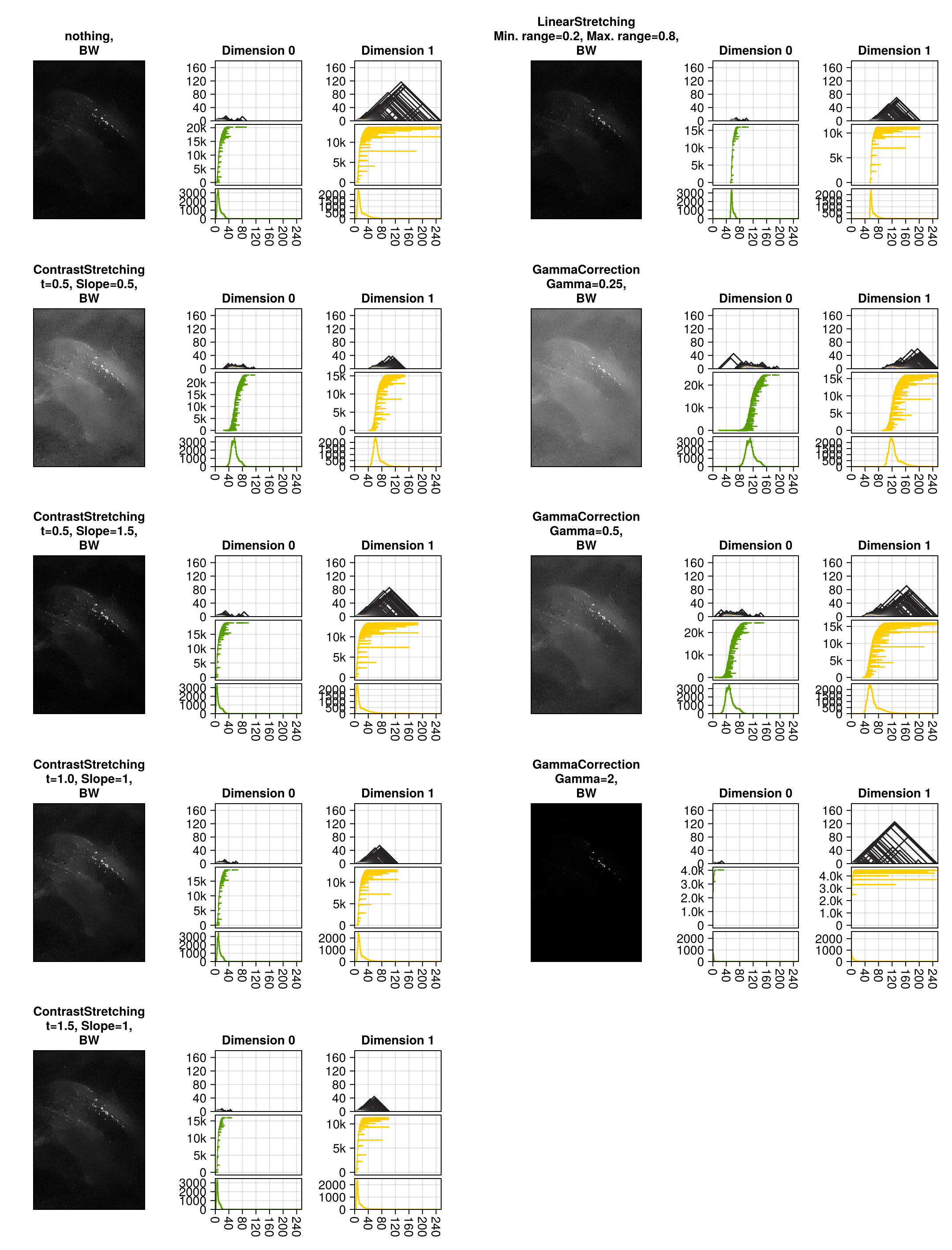

Supplement: S25 Fig — Different histogram transformations are indicated in the titles above the transformed images. Details about histogram manipulations are presented in S2 Table. For each transformation, topological features are extracted for BW filtration and for each transformation, persistence landscapes, persistence barcodes, and Betti curves are shown for dimensions 0 and 1. (PNG) [file pcbi.1014156.s025.png]

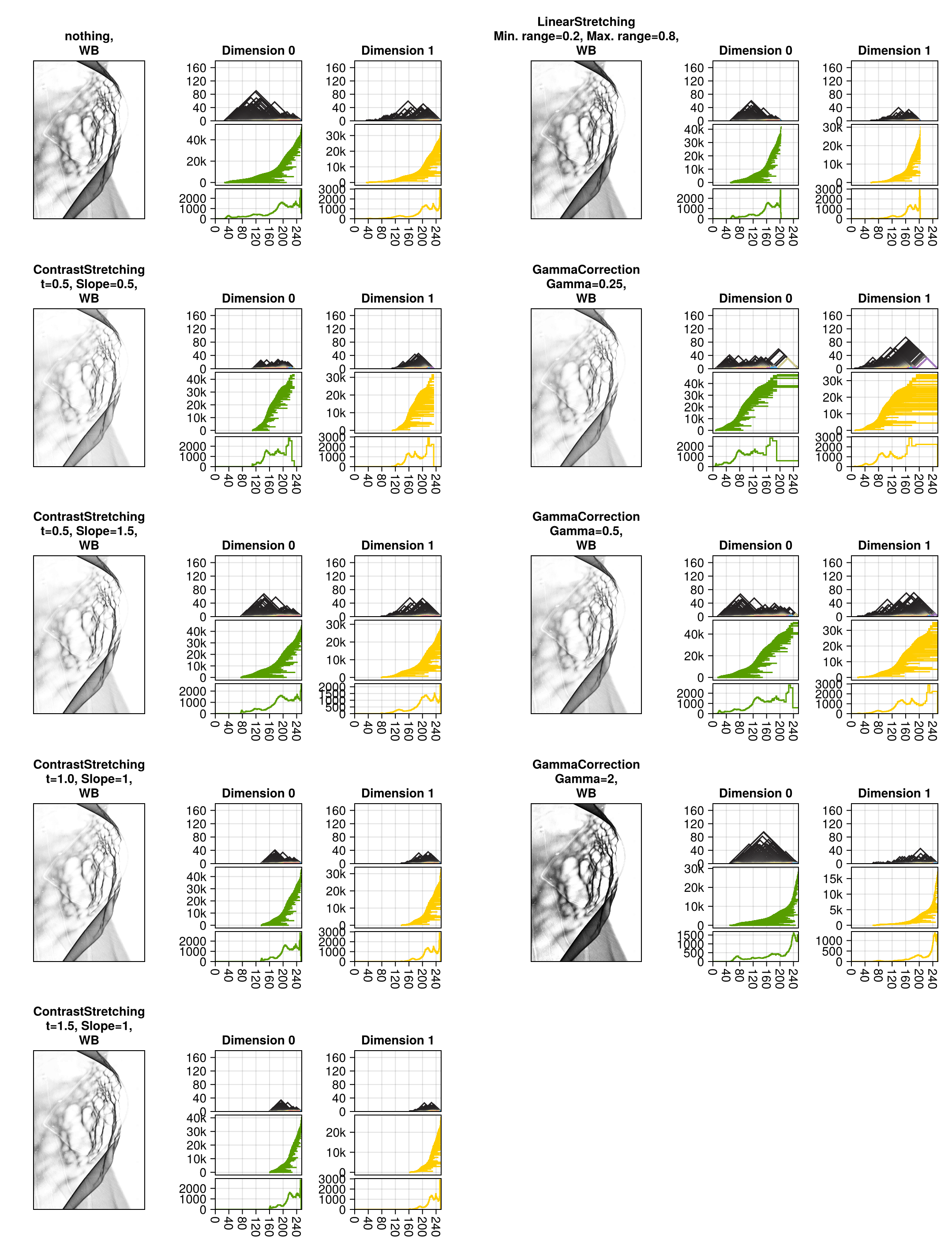

Supplement: S26 Fig — Different histogram transformations are indicated in the titles above the transformed images. Details about histogram manipulations are presented in S2 Table. For each transformation, topological features are extracted for BW filtration and for each transformation, persistence landscapes, persistence barcodes, and Betti curves are shown for dimensions 0 and 1. (PNG) [file pcbi.1014156.s026.png]

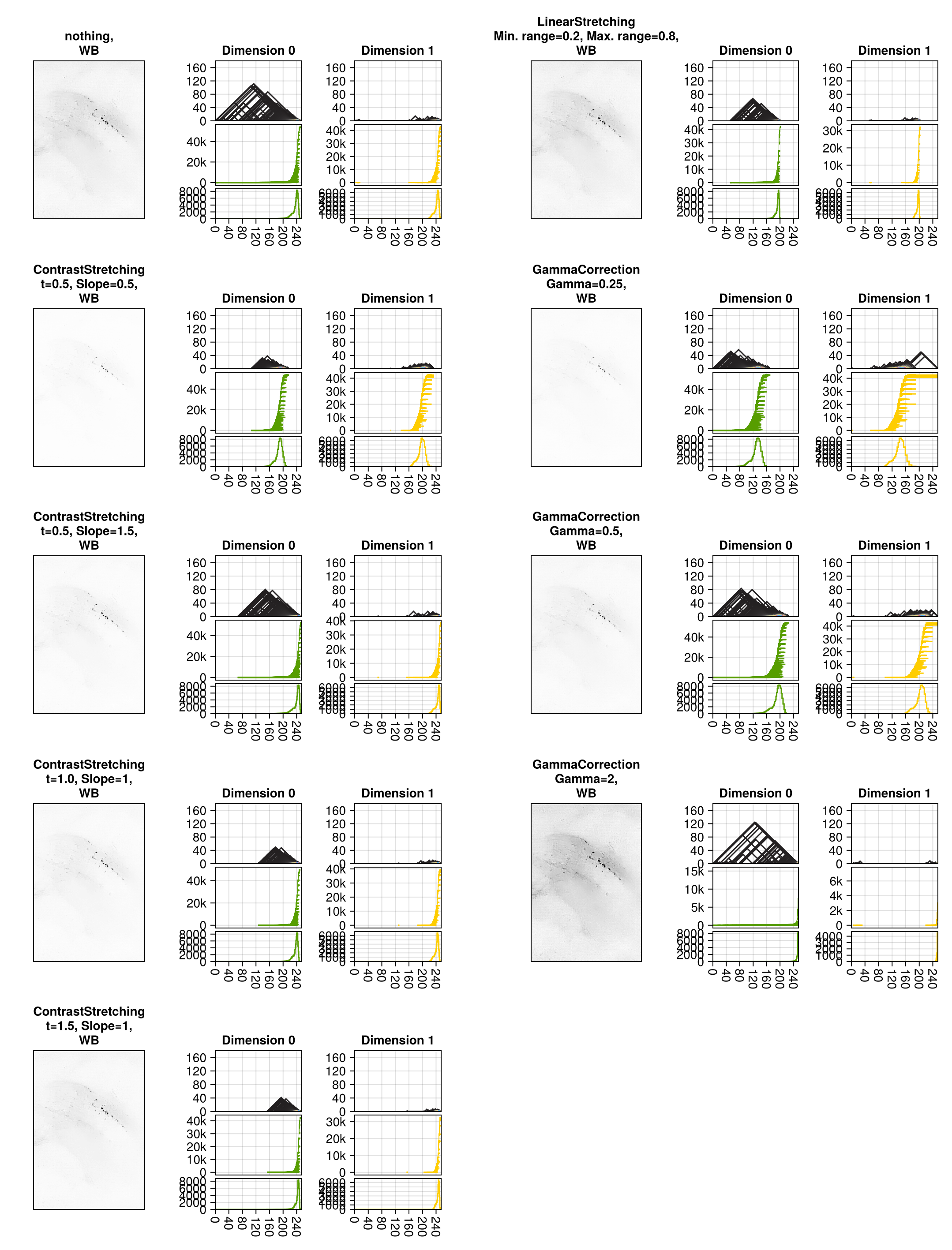

Supplement: S27 Fig — Different histogram transformations are indicated in the titles above the transformed images. Details about histogram manipulations are presented in S2 Table. For each transformation, topological features are extracted for BW filtration and for each transformation, persistence landscapes, persistence barcodes, and Betti curves are shown for dimensions 0 and 1. (PNG) [file pcbi.1014156.s027.png]

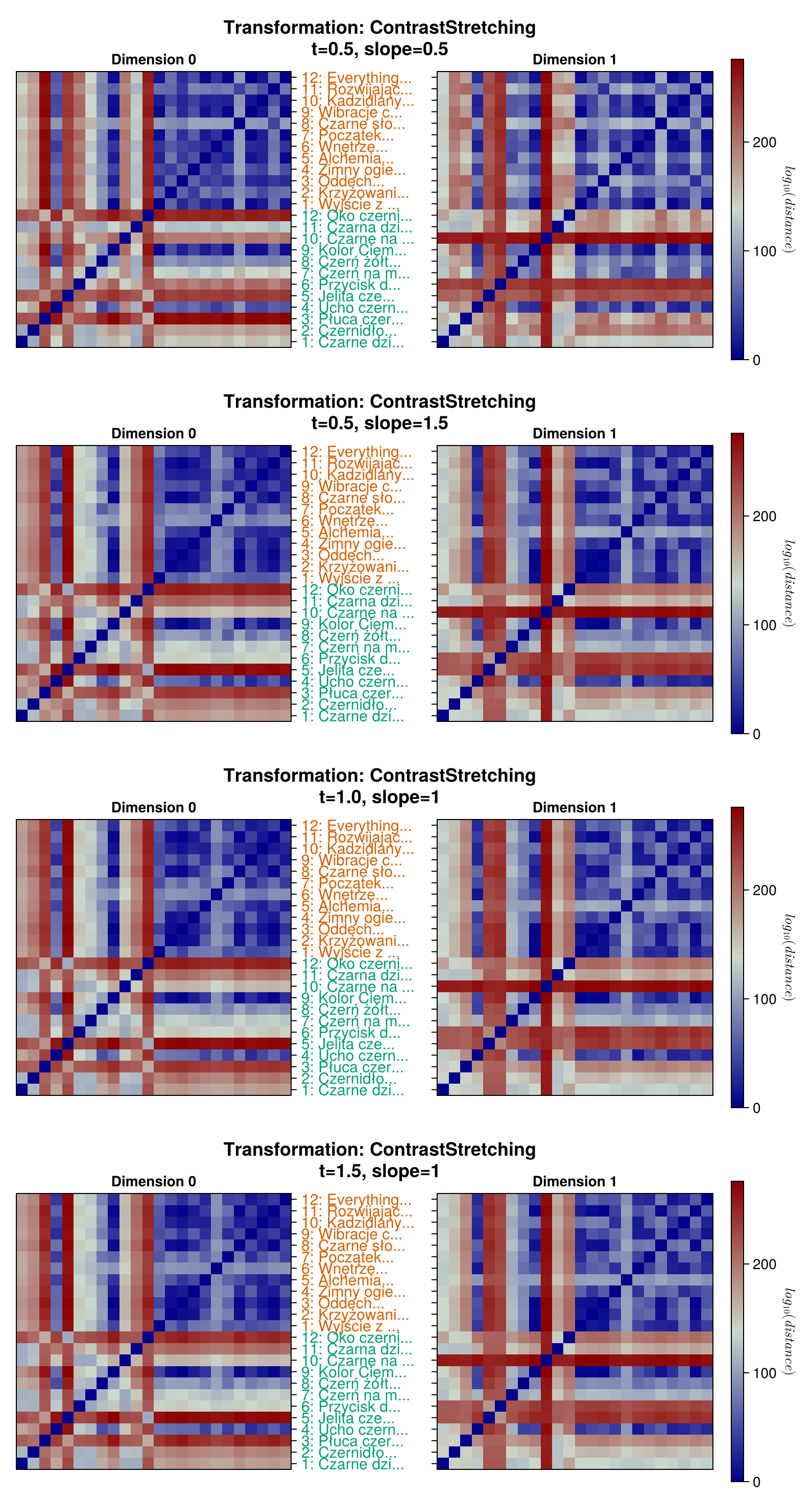

Supplement: S28 Fig — All presented results are for filtration from black to white. Each heatmap displays L1 pairwise differences between the persistence landscapes of all images in dimensions 0 (left) and 1 (right). Every row (or column) in the heatmap corresponds to an image, annotated between the matrices, to which a transformation was applied as indicated by the titles. The top 12 labels (coloured orange) are from pseudo-artistic images, and the next 12 labels (coloured green) are from artistic images. The overall shape differences captured with the L1 distance between landscapes across transformation are preserved. The relation between images is preserved, that is, in all cases, the distance between pseudo-artistic images was lower than for art images. More details about how the distance is computed is presented in S10 Fig. (PNG) [file pcbi.1014156.s028.png]

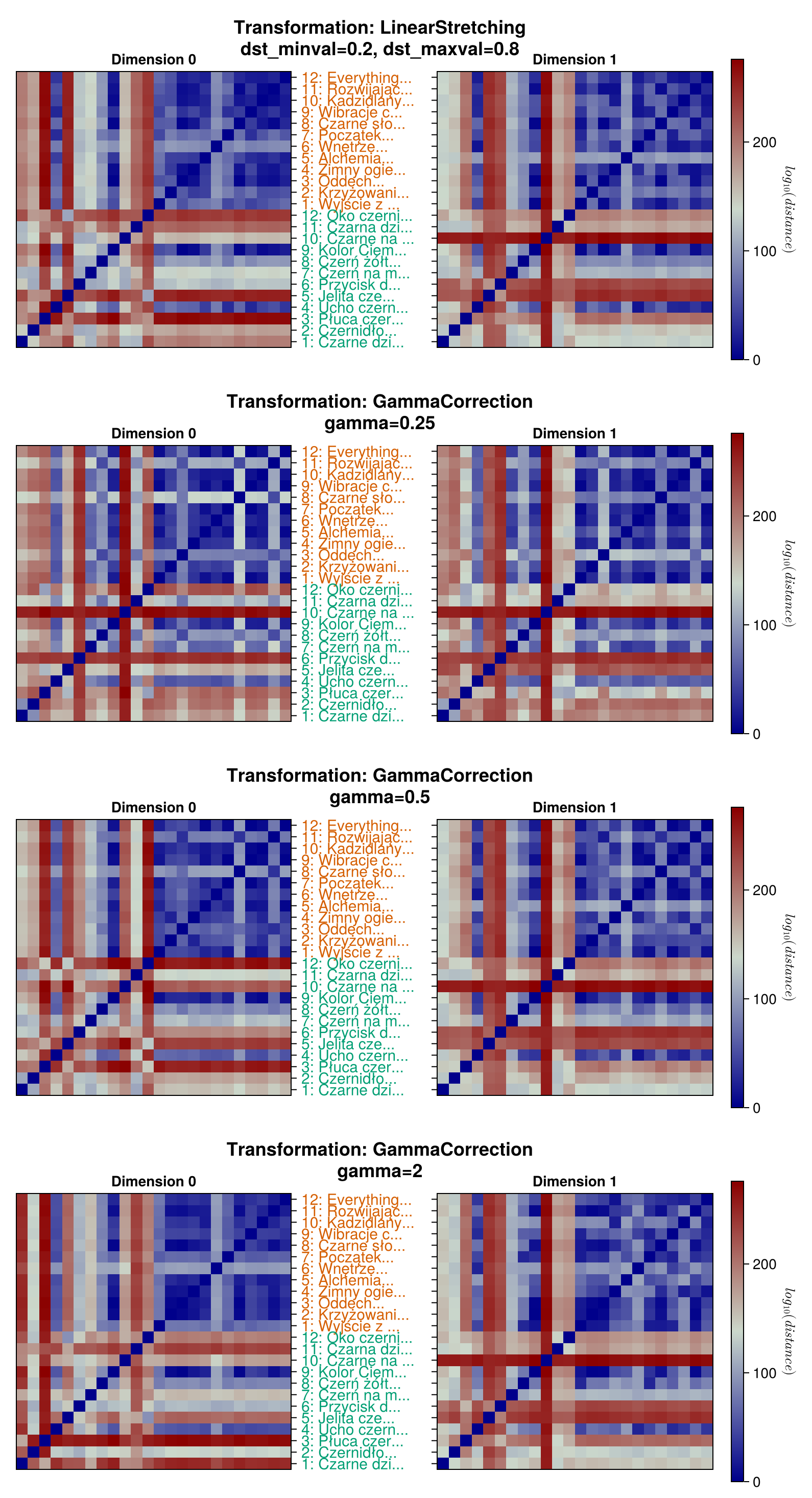

Supplement: S29 Fig — All presented results are for filtration from black to white. Each heatmap displays L1 pairwise differences between the persistence landscapes of all images in dimensions 0 (left) and 1 (right). Every row (or column) in the heatmap corresponds to an image, annotated between the matrices, to which a transformation was applied as indicated by the titles. The top 12 labels (coloured orange) are from pseudo-artistic images, and the next 12 labels (coloured green) are from artistic images. The overall shape differences captured with the L1 distance between landscapes across transformation are preserved. More details about how the distance is computed is presented in S10 Fig. (PNG) [file pcbi.1014156.s029.png]

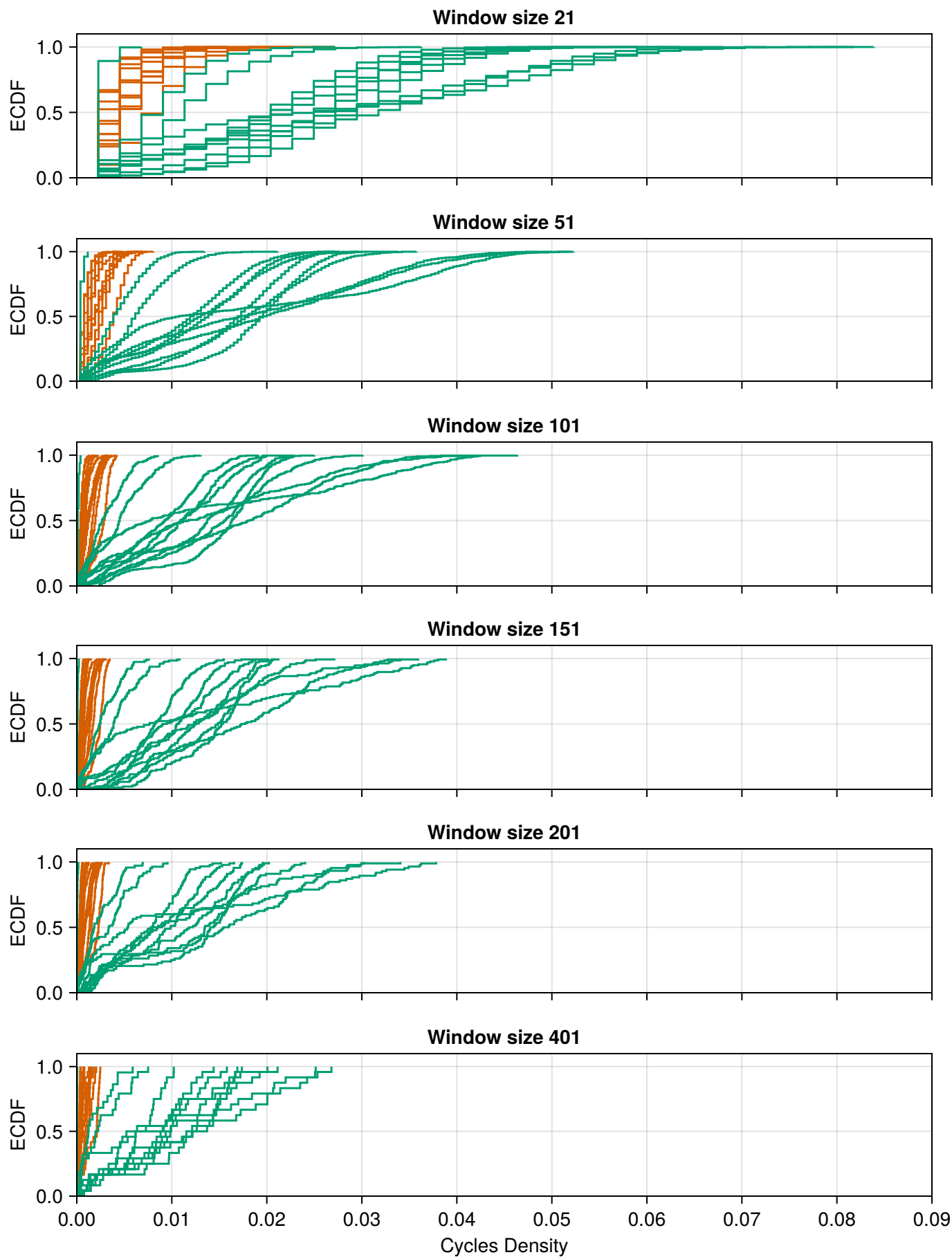

**Dataset**

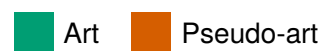

Supplement: S30 Fig — The ECDFs for artistic images are marked green and the pseudo-artistic images orange for different window sizes (plots from top to bottom): 21, 51, 101, 151, 201, 401. (PDF) [file pcbi.1014156.s030.pdf]

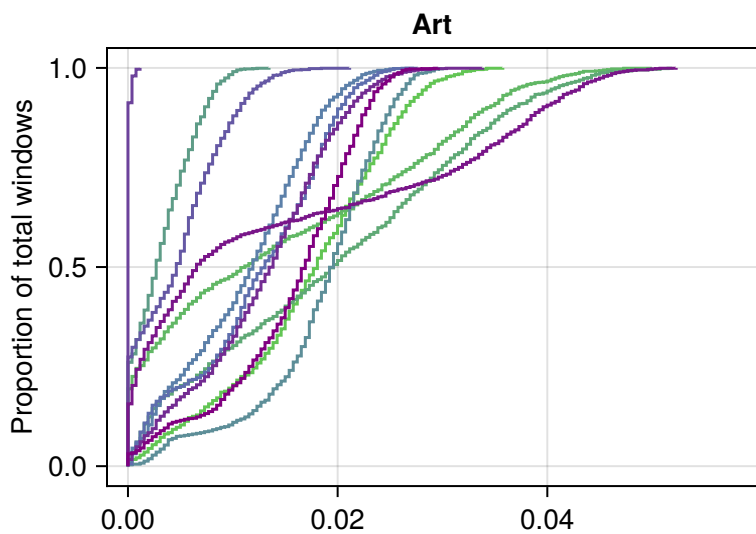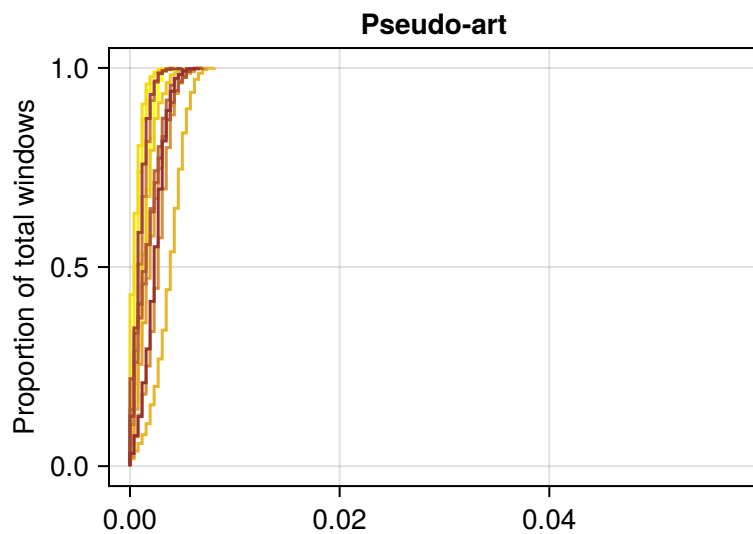

Black to white filtration

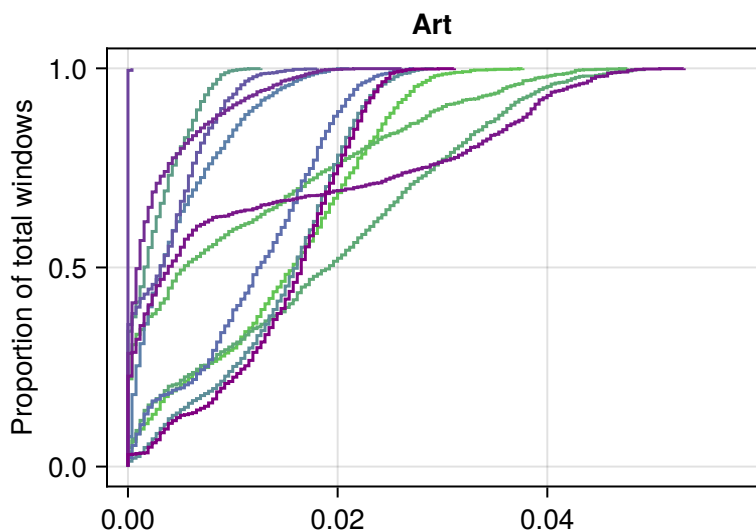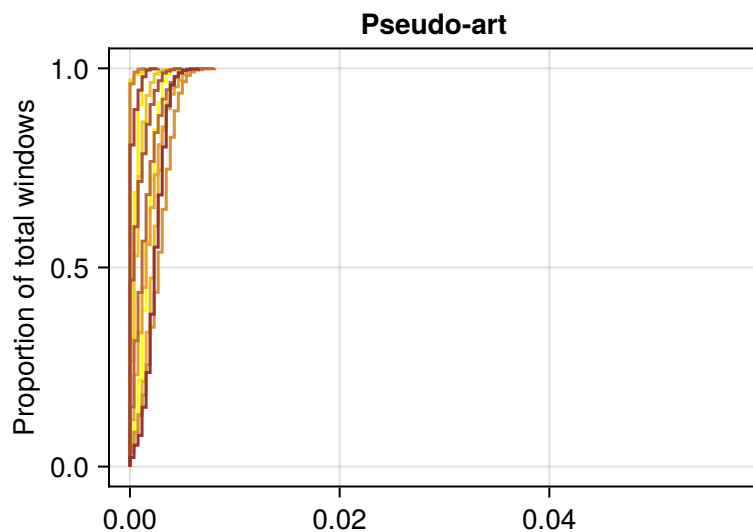

White to black filtration

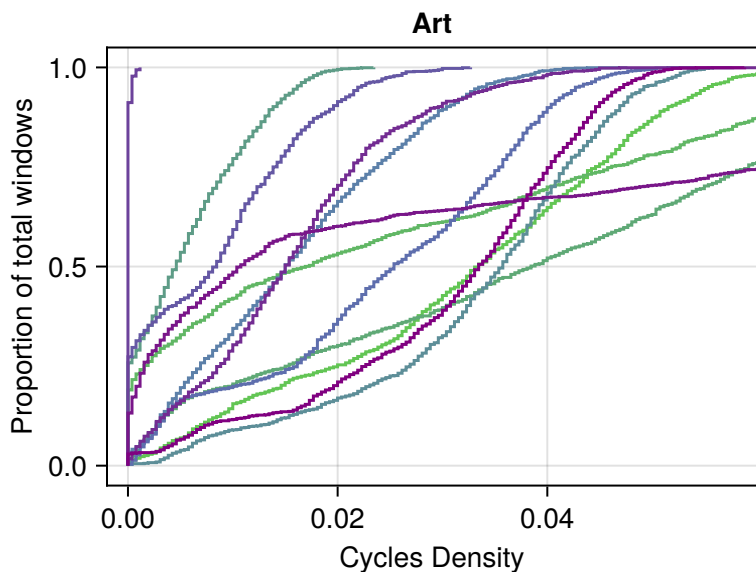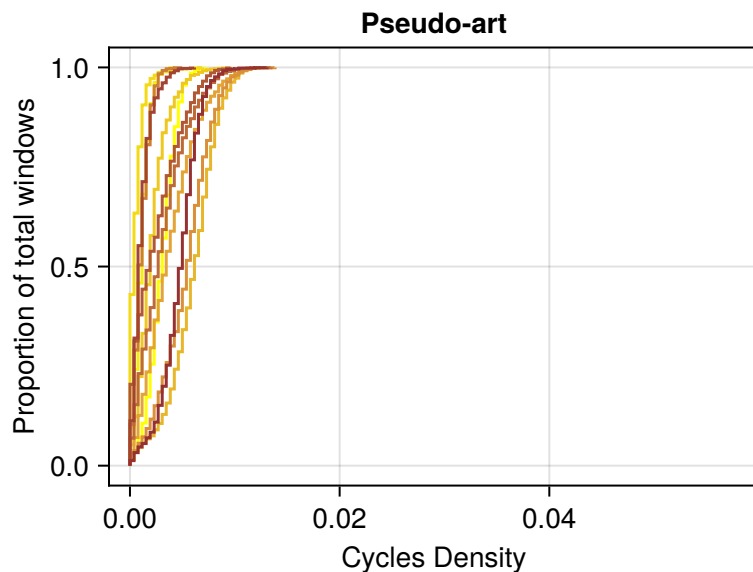

Combined filtration

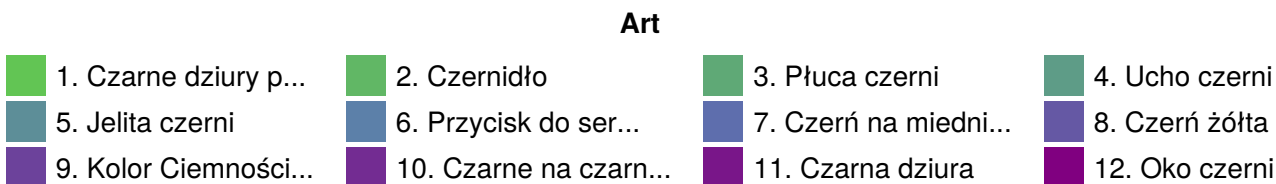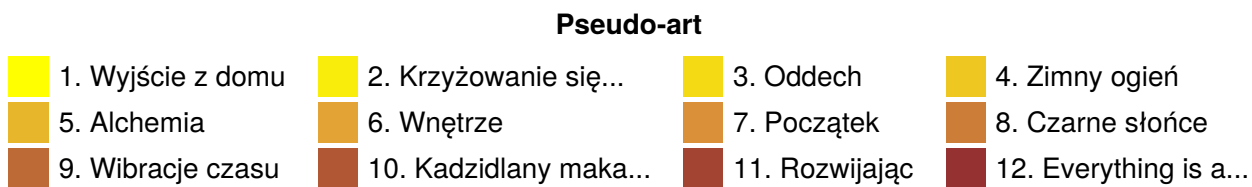

Supplement: S31 Fig — ECDF(Mdensity,U), for: BW filtration (top row), WB filtration (middle row), combined BW and WB filtration (bottom row). (PDF) [file pcbi.1014156.s031.pdf]

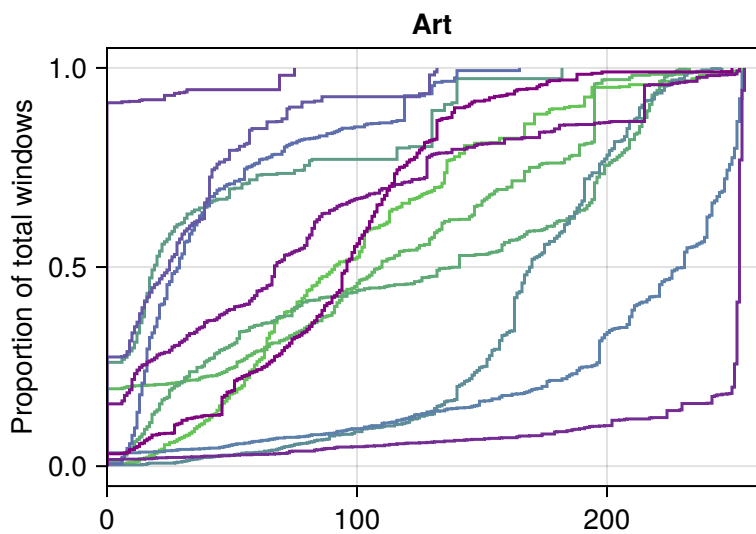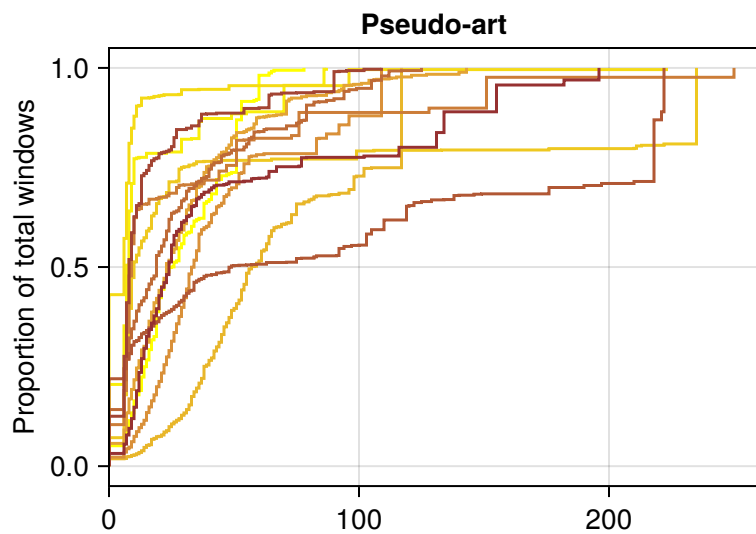

Black to white filtration

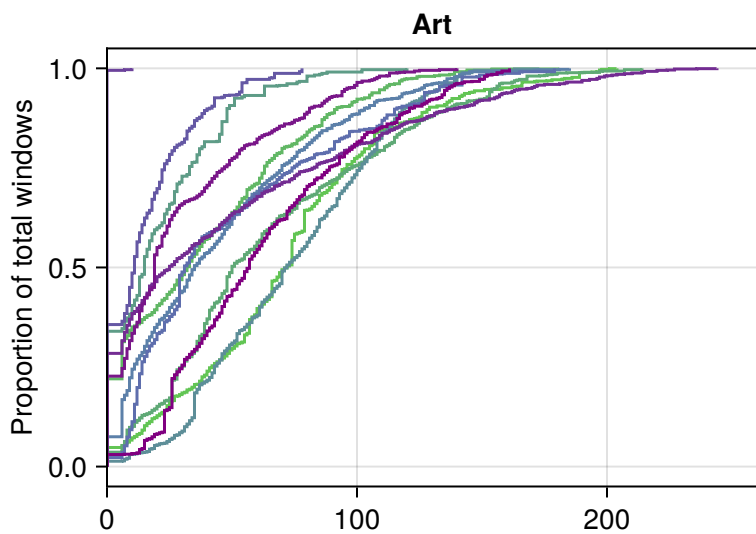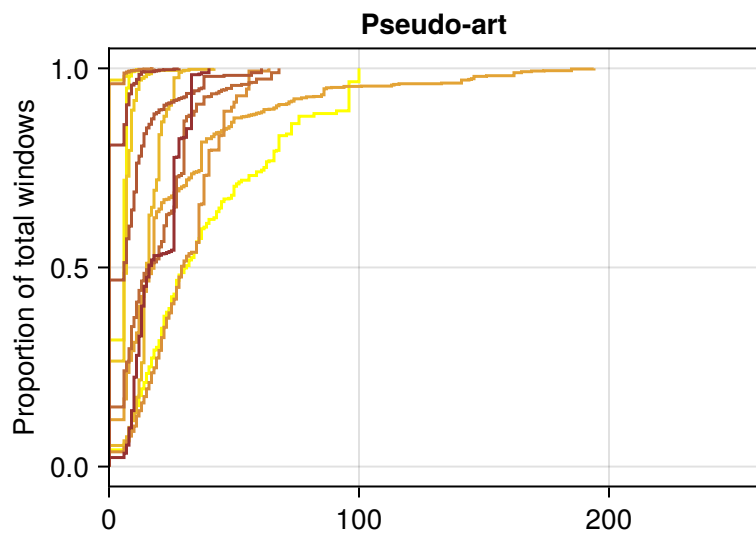

White to black filtration

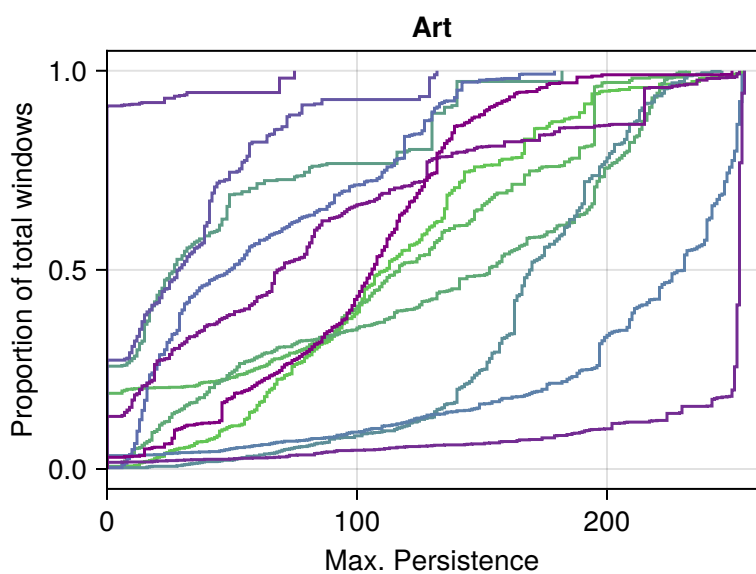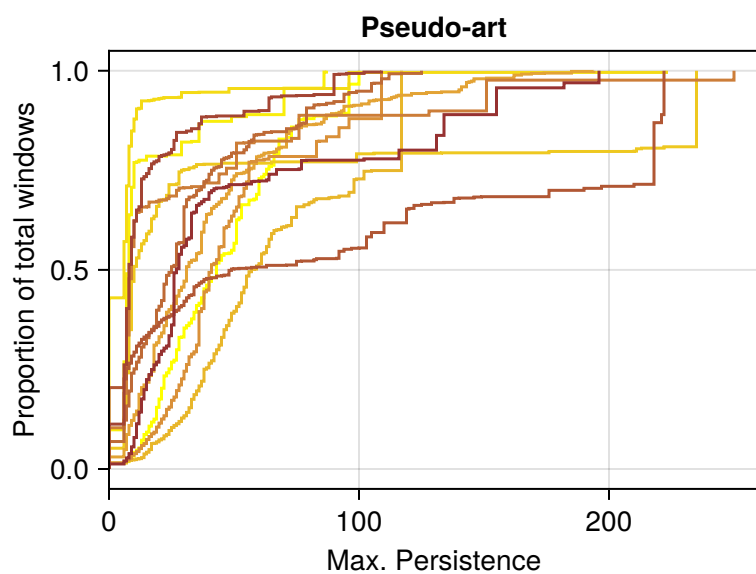

Combined filtration

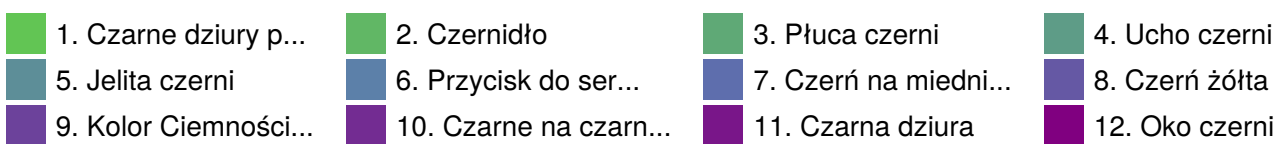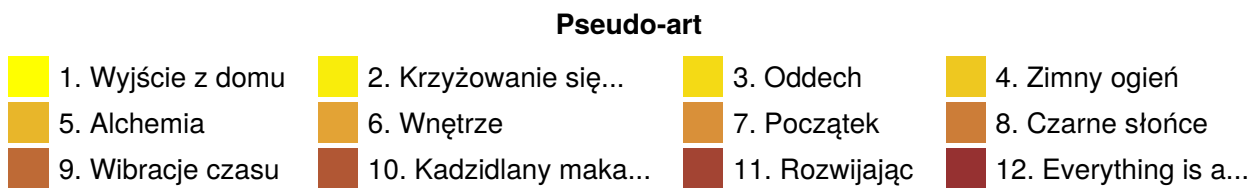

Supplement: S32 Fig — ECDF(Mpersistence,U), for: BW filtration (top row), WB filtration (middle row), combined BW and WB filtration (bottom row). (PDF) [file pcbi.1014156.s032.pdf]

a)

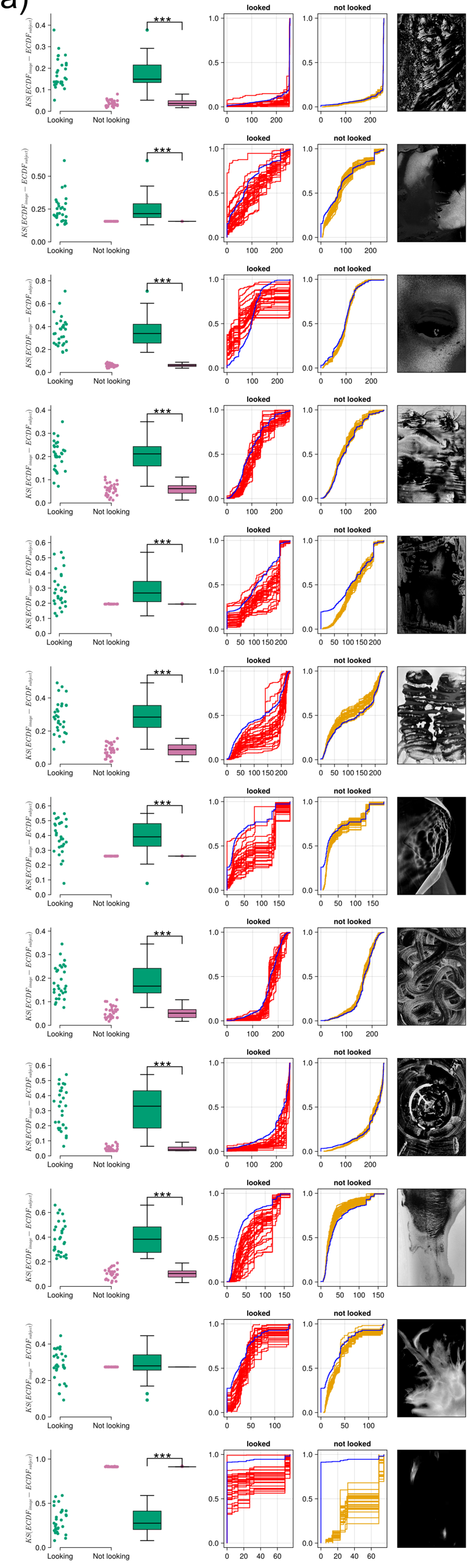

b)

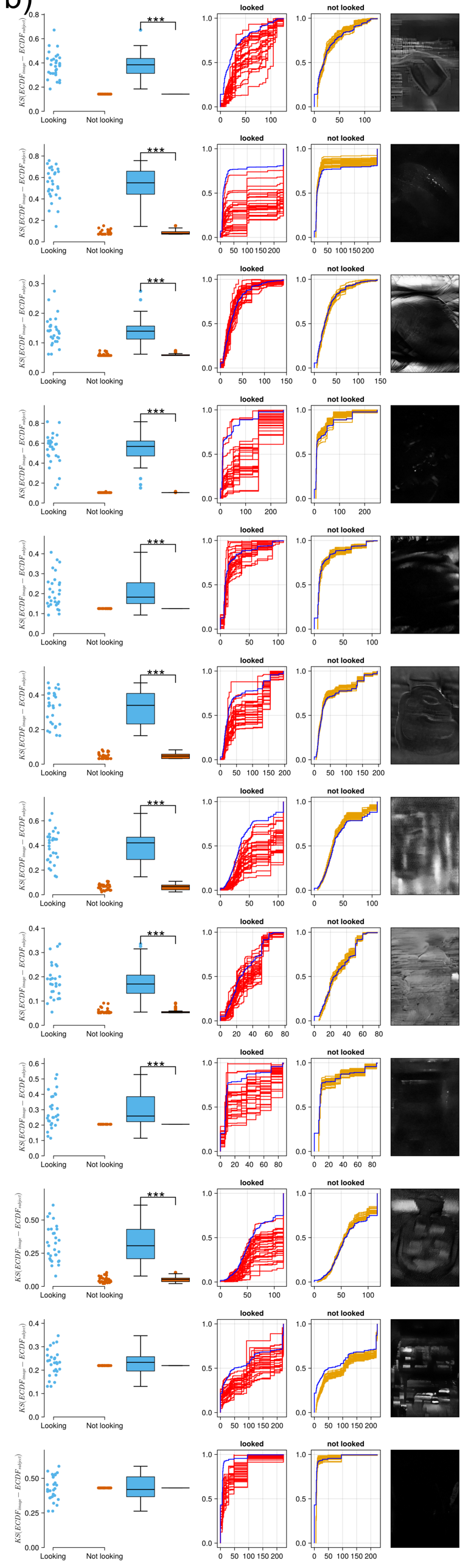

Supplement: S34 Fig — (a) data shown for art [11]. (b) data shown for pseudo art. Both sessions. Within a column the plots are (from left to right): scatterplot of the Kolmogorov-Smirnov statistic between the image’s ECDF vs ‘looking’ ECDF and images’s ECDF vs ‘not looking’ ECDF for each person; bar plot of the same; the ‘Looking ECDF’ for each person; ‘Not looking ECDF’ for each person; the image itself. The blue ECDF is intrinsic to the image itself and can be thought to be arising from a ‘gaze’ that is a uniform scan of the entire image. Results for the full set of ECDFs. For each image k, feature map M and participant s, we have ECDF(Mk,U) (‘intrinsic’), ECDF(Mk,Gs) (where the participant was ‘looking’), ECDF(Mk,Gs~) (where the participant was ‘not looking’). (PDF) [file pcbi.1014156.s034.pdf]

a)

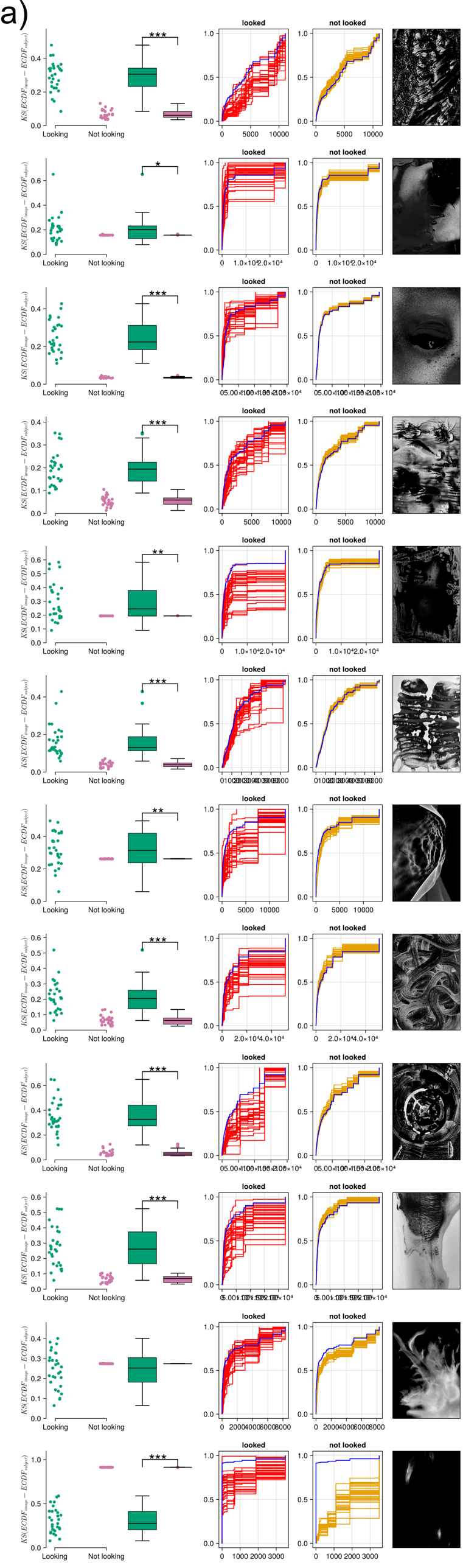

b)

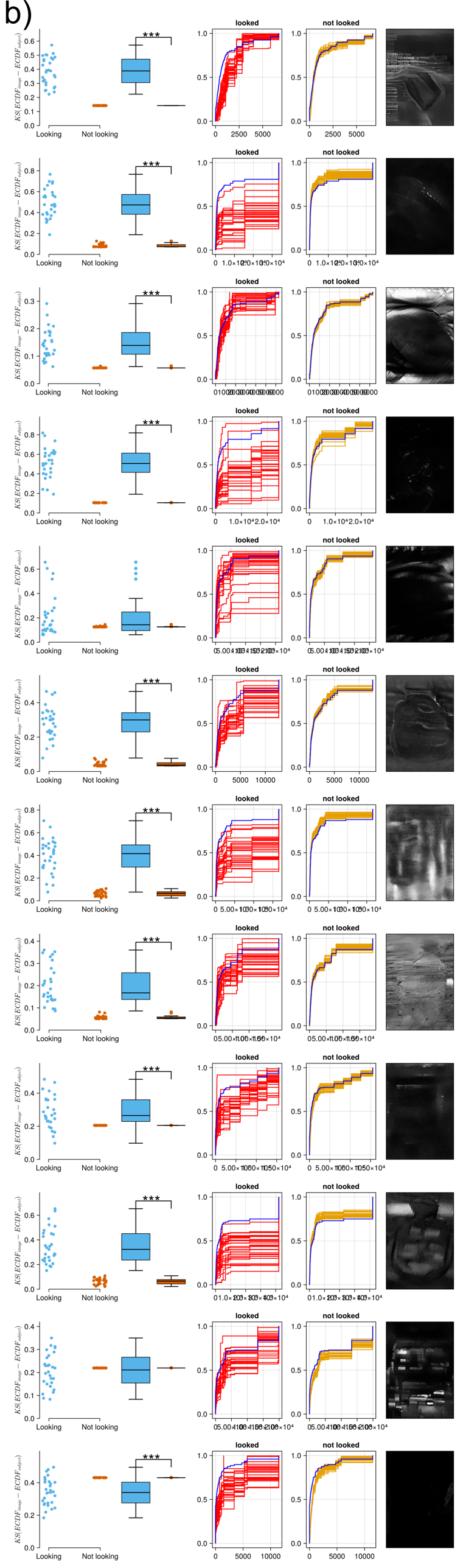

Supplement: S35 Fig — (a) data shown for art [11]. (b) data shown for pseudo art. Both sessions. Within a column, the plots are (from left to right): scatterplot of the Kolmogorov-Smirnov statistic between the image’s ECDF vs ‘looking’ ECDF and images’s ECDF vs ‘not looking’ ECDF for each person; bar plot of the same; the ‘Looking ECDF’ for each person; ‘Not looking ECDF’ for each person; the image itself. The blue ECDF is intrinsic to the image itself and can be thought to be arising from a ‘gaze’ that is a uniform scan of the entire image. (PDF) [file pcbi.1014156.s035.pdf]

a)

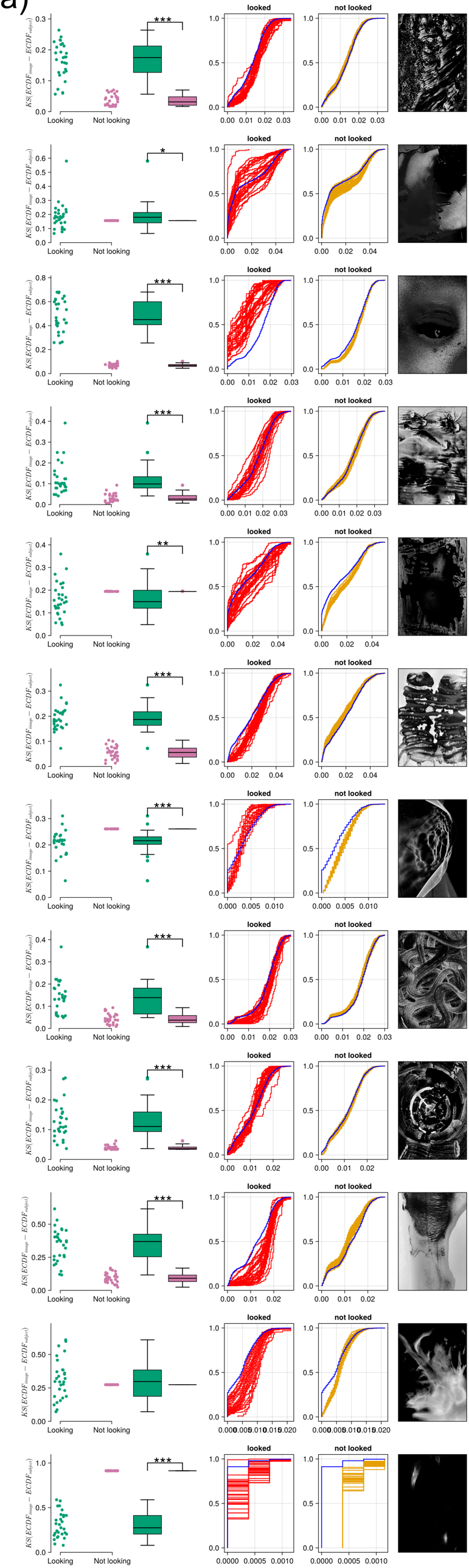

b)

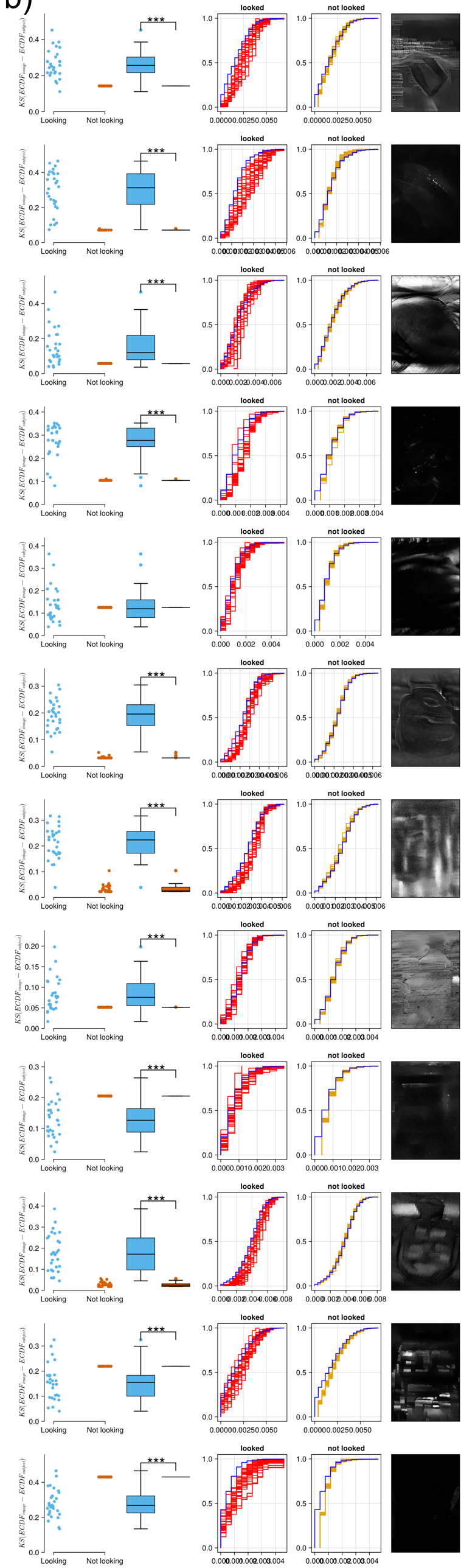

Supplement: S36 Fig — (a) data shown for art [11]. (b) data shown for pseudo art. Both sessions. Within a column, the plots are (from left to right): scatterplot of the Kolmogorov-Smirnov statistic between the image’s ECDF vs ‘looking’ ECDF and images’s ECDF vs ‘not looking’ ECDF for each person; bar plot of the same; the ‘Looking ECDF’ for each person; ‘Not looking ECDF’ for each person; the image itself. The blue ECDF is intrinsic to the image itself and can be thought to be arising from a ‘gaze’ that is a uniform scan of the entire image. (PDF) [file pcbi.1014156.s036.pdf]
