# Supplementary material for: Art’s hidden topology: A window into human perception
Source: PLoS Comput Biol. 2026 May 14;22(5):e1014156. doi: 10.1371/journal.pcbi.1014156 (PMC13175340; doi:10.1371/journal.pcbi.1014156)
Supplement: S3 Fig — Original tiles are given under each image. (PDF) [file pcbi.1014156.s003.pdf]

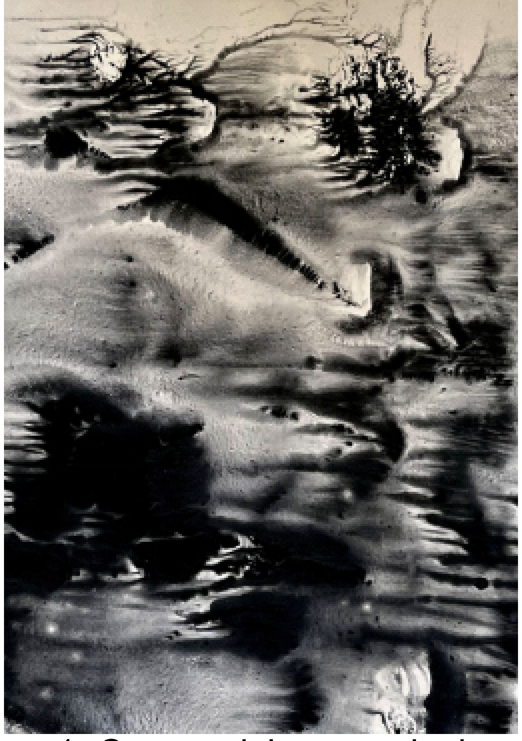

1. Czarne dziury pamięci
(eng. “Black holes of blackness”)

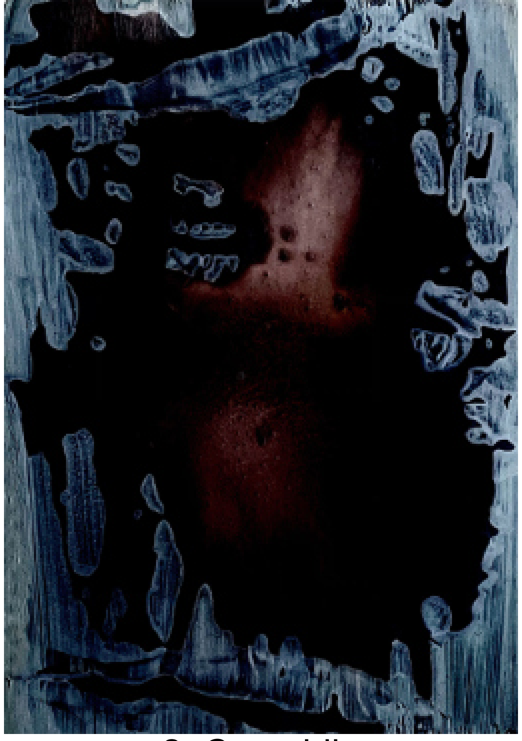

2. Czernidło
(eng. “Black wash”)

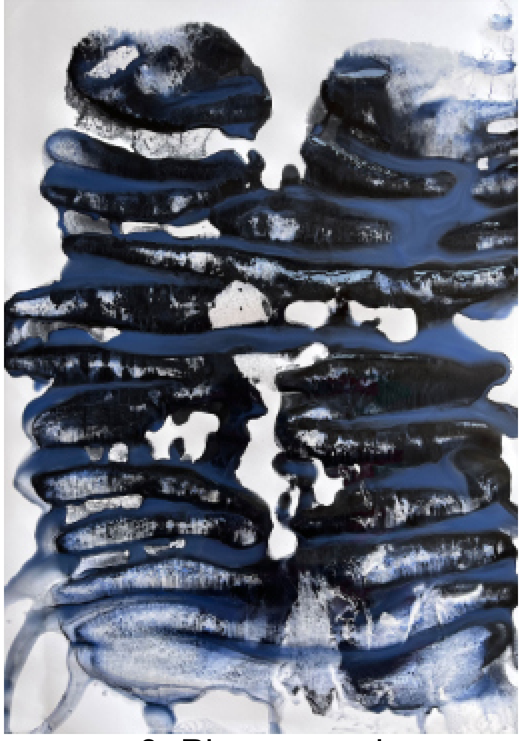

3. Płuca czerni
(eng. “Lungs of blackness”)

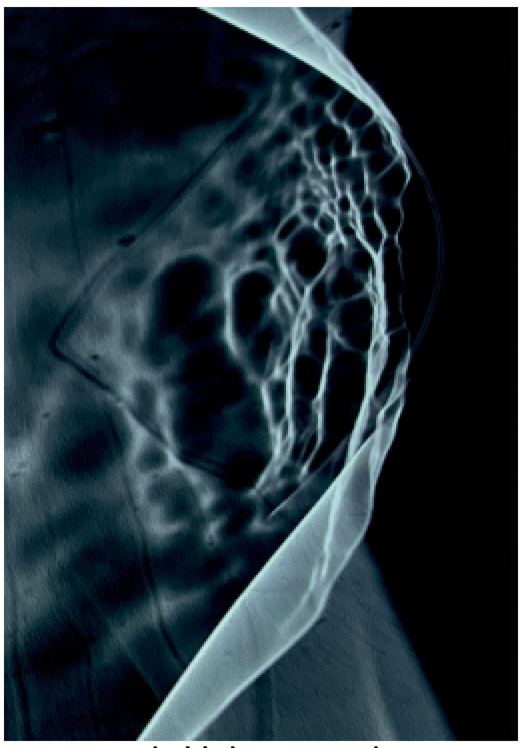

4. Ucho czerni
(eng. “Ear of blackness”)

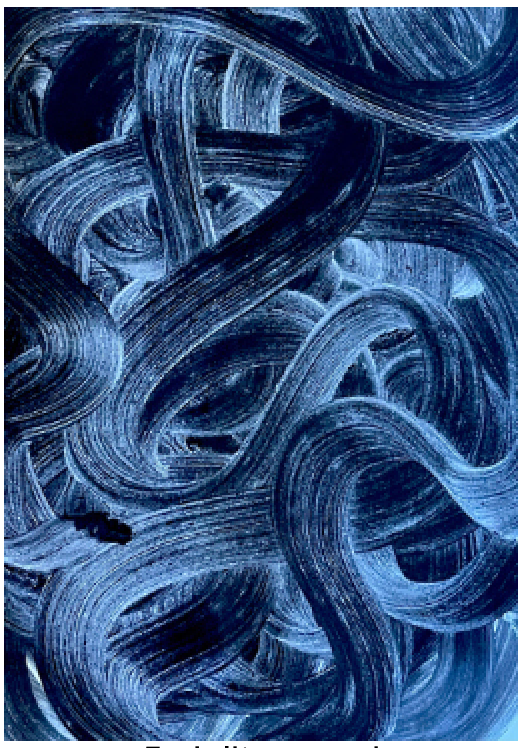

5. Jelita czerni
(eng. “Guts of blackness”)

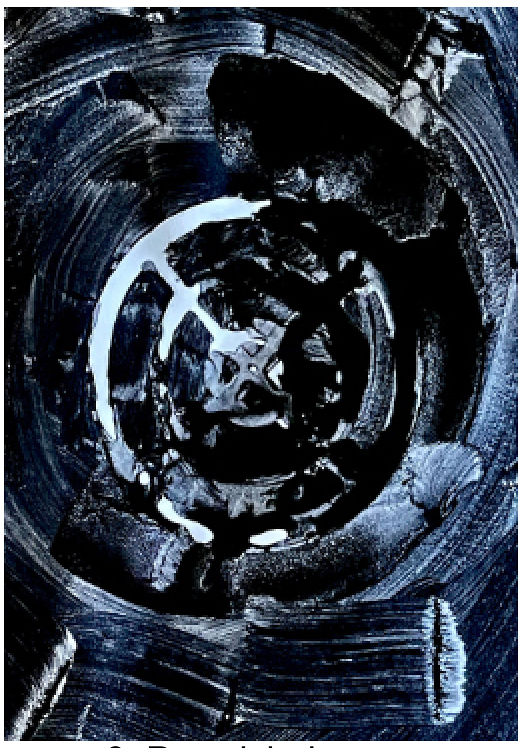

6. Przycisk do serc
(eng. “Hearts button”)

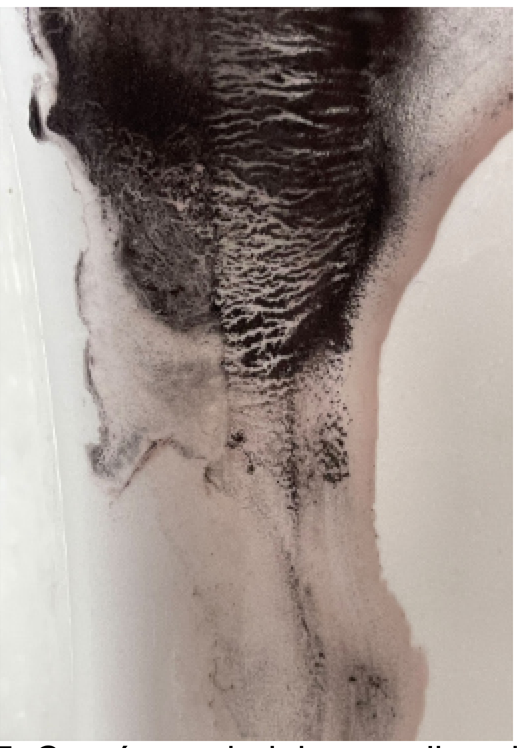

7. Czerń na miednicy emaliowej
(eng. “Blackness on enamel washing basin”)

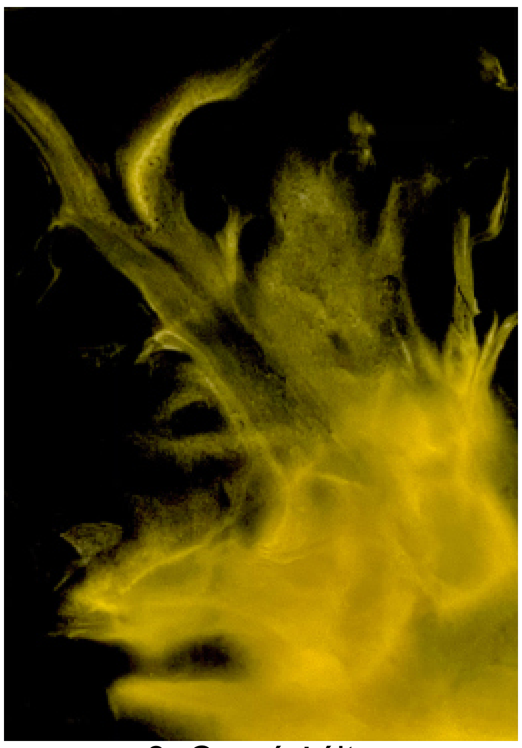

8. Czerń żółta
(eng. “Yellow blackness”)

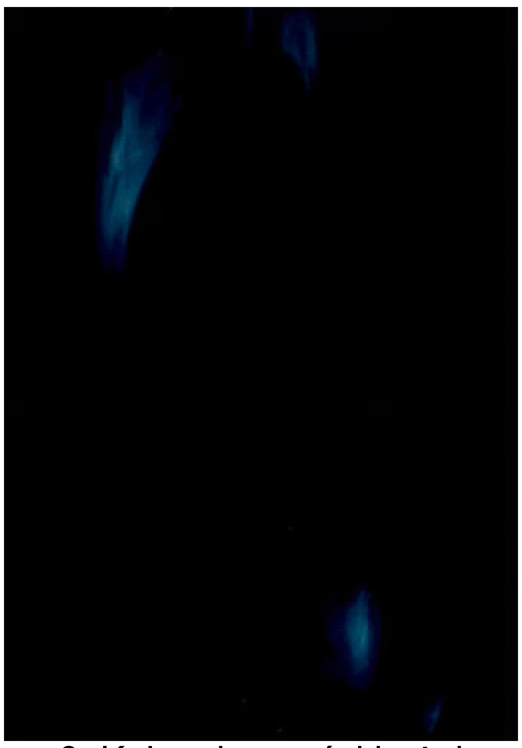

9. Kolor ciemności bożej
(eng. “The colour of holy blackness”)

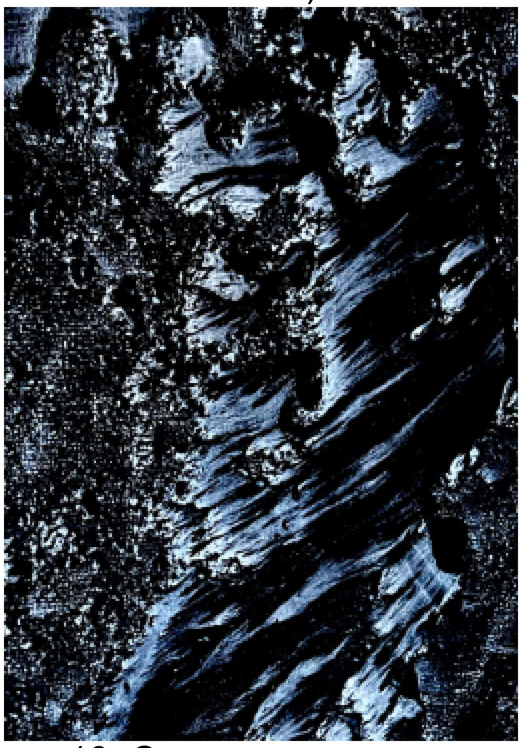

10. Czarne na czarnym
(eng. “Black on black”)

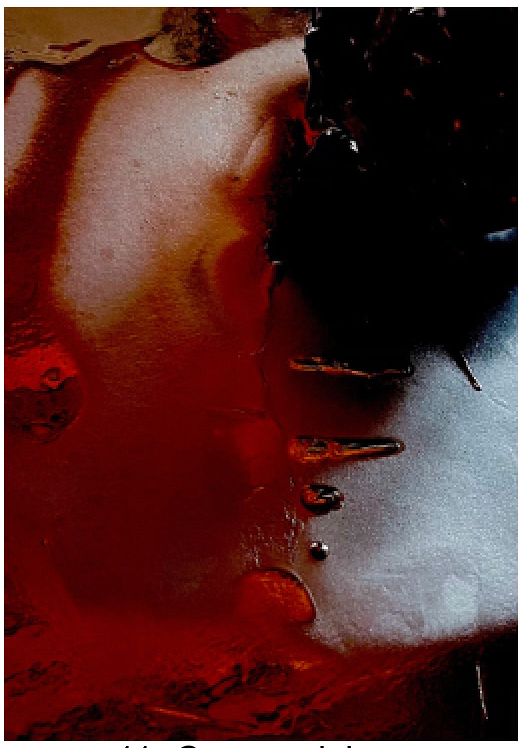

11. Czarna dziura
(eng. “Black hole”)

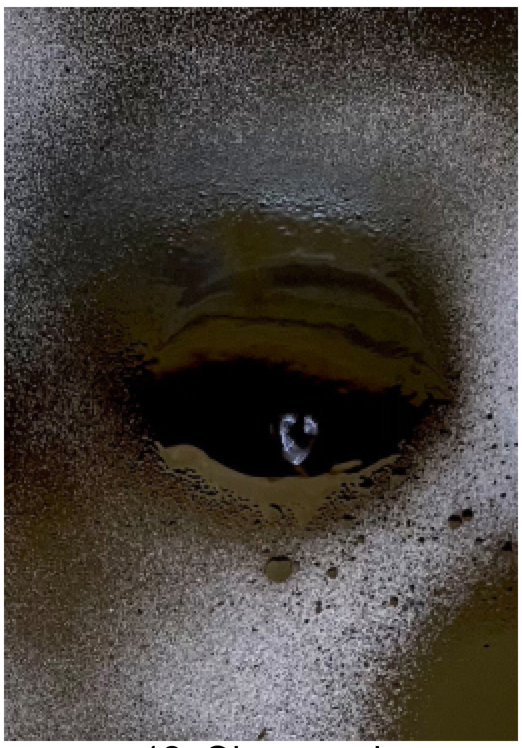

12. Oko czerni
(eng. “Eye of blackness”)
