# Supplementary material for: Art’s hidden topology: A window into human perception
Source: PLoS Comput Biol. 2026 May 14;22(5):e1014156. doi: 10.1371/journal.pcbi.1014156 (PMC13175340; doi:10.1371/journal.pcbi.1014156)
Supplement: S4 Fig — Original tiles are given under each image. (PDF) [file pcbi.1014156.s004.pdf]

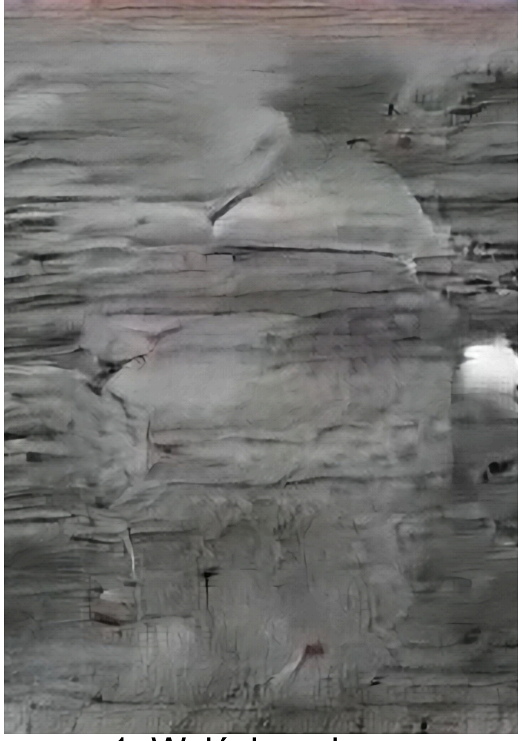
1. Wyjście z domu
(eng. “Leaving home”)

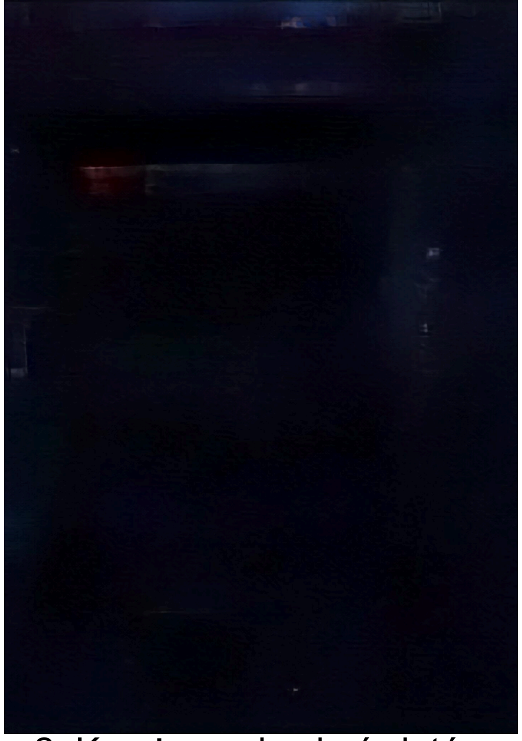
2. Krzyżowanie się światów
(eng. “The crossing of worlds”)

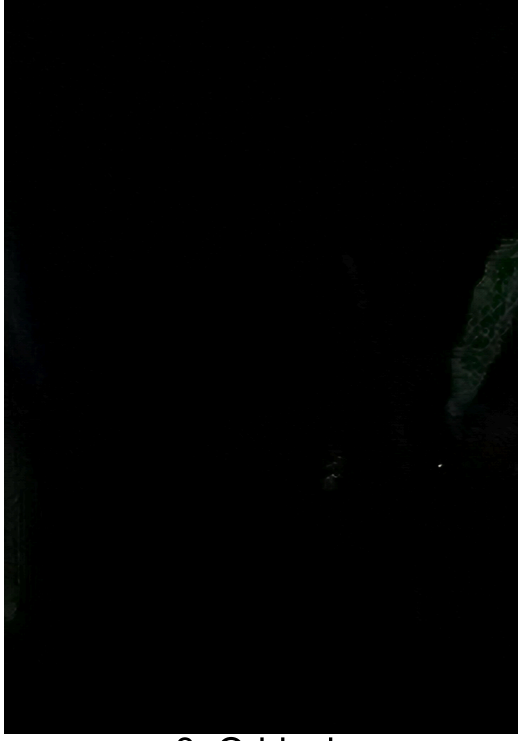
3. Oddech
(eng. “Breath”)

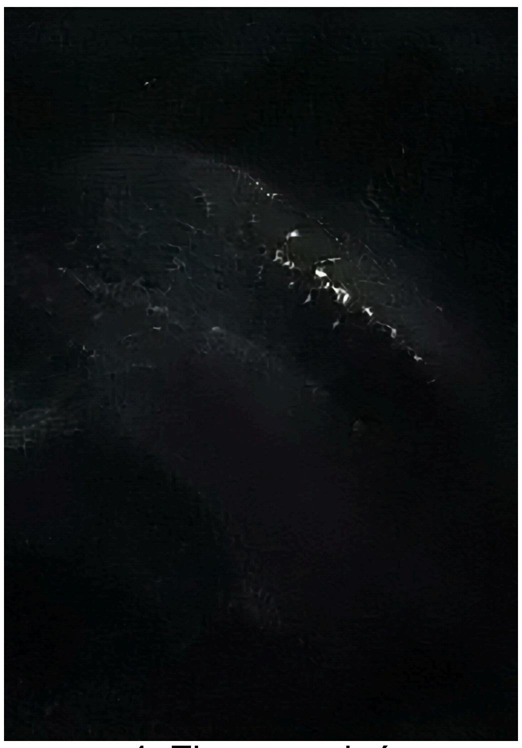
4. Zimny ognień
(eng. “Cold fire”)

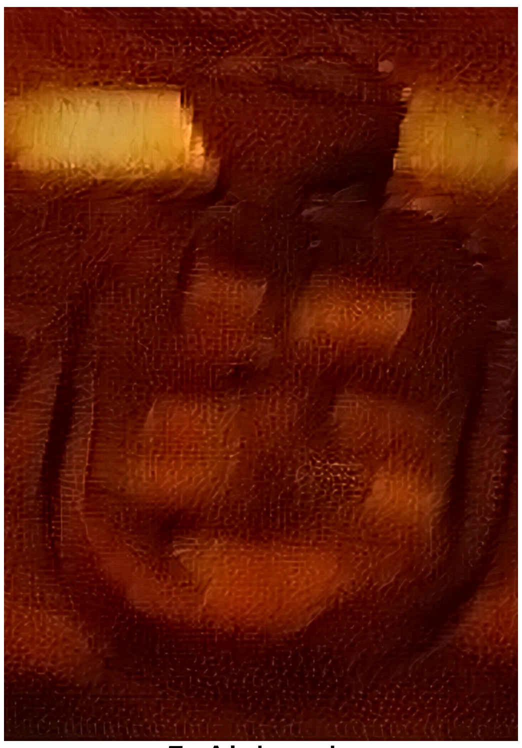
5. Alchemia
(eng. “Alchemy”)

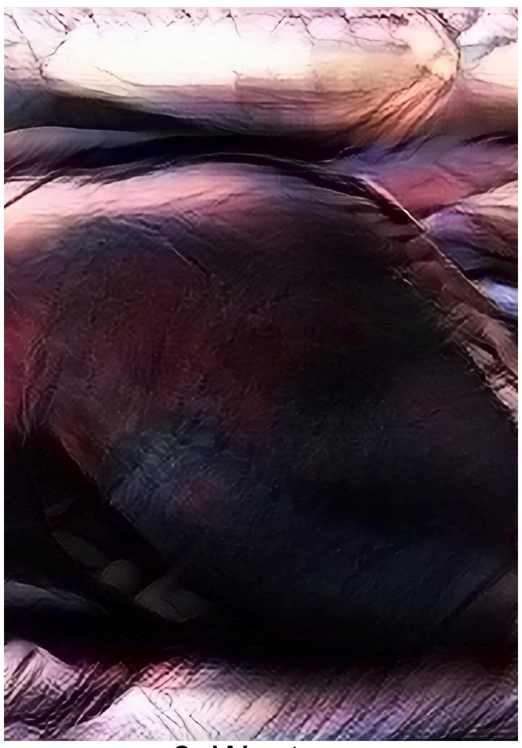
6. Wnętrze
(eng. “The Inside”)

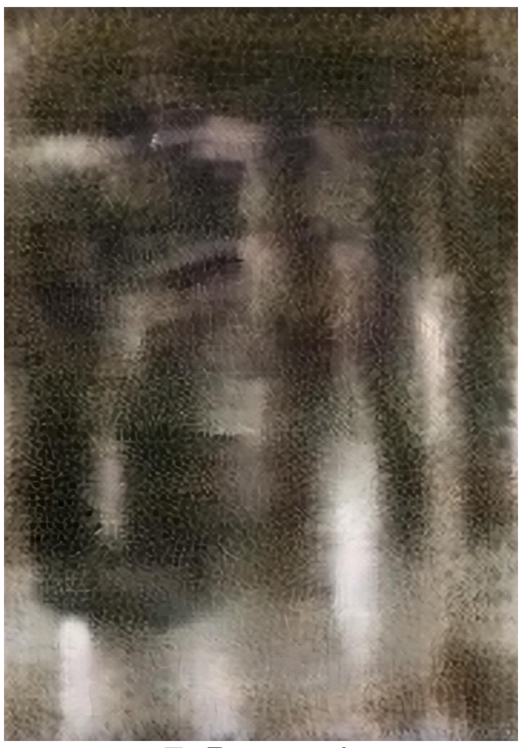
7. Początek
(eng. “The beginning”)

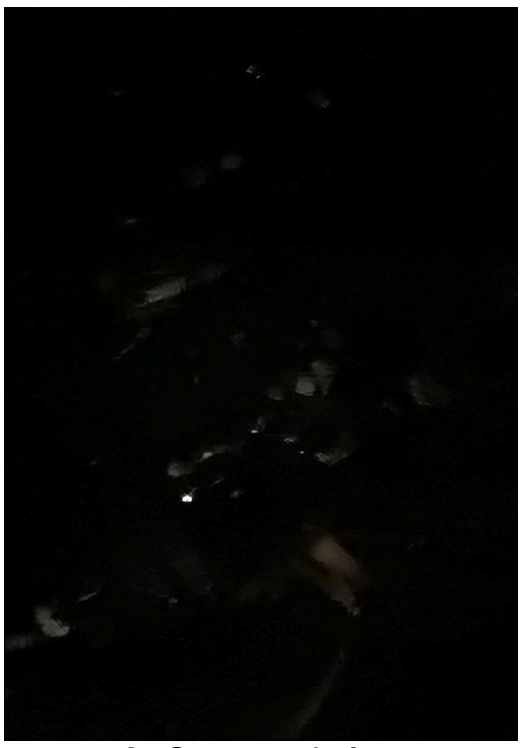
8. Czarne słońce
(eng. “Black sun”)

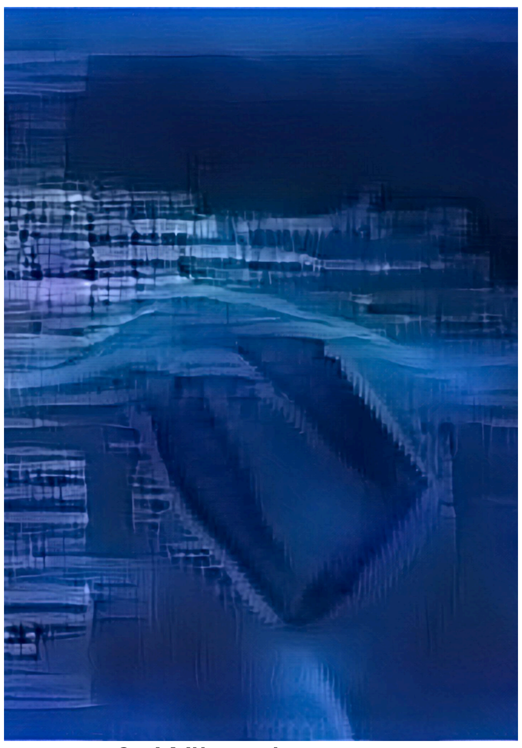
9. Wibracje czasu
(eng. “Vibrations of time”)

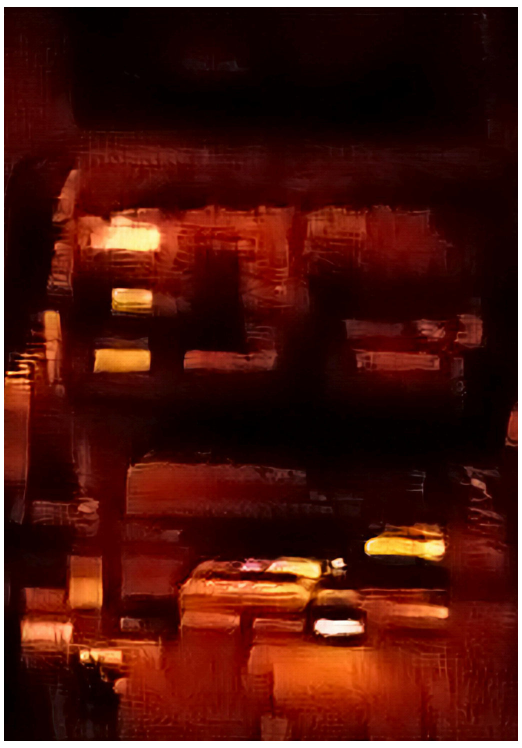
10. Kadzidlany Makat- delikatne pochodzenie i rosnąca SI
(eng. “Incense Makat- delicate origin and growing SI”)

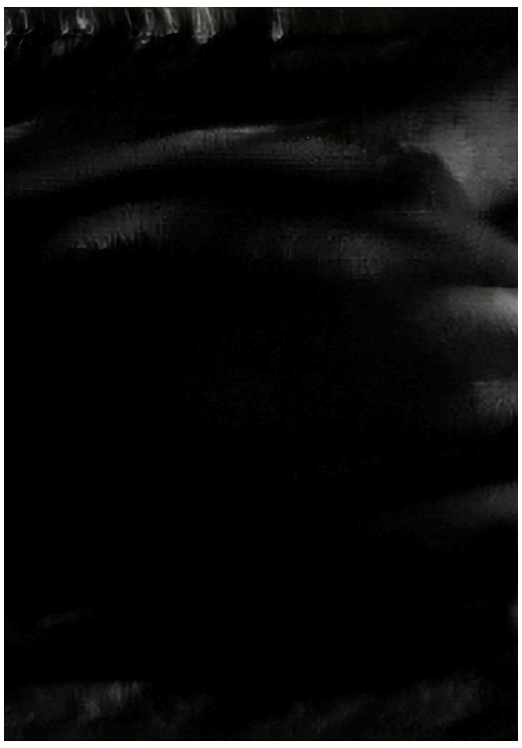
11. Rozwijając
(eng. “Unfolding”)

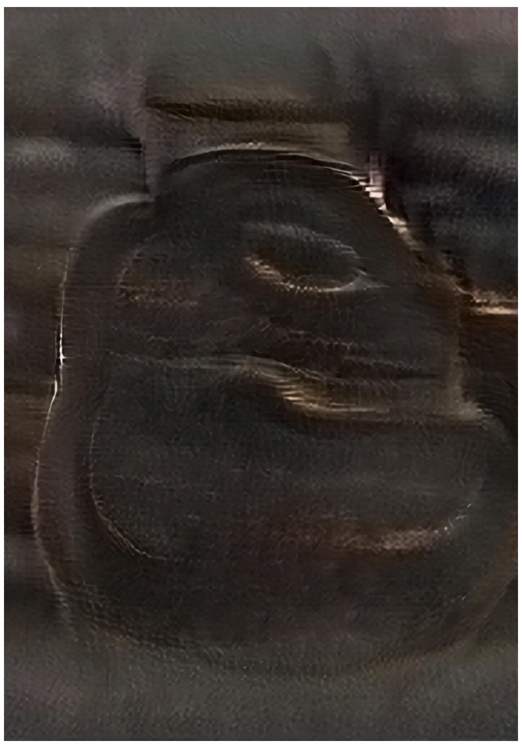
12. EVRYTHING IS A THING AND NOTHING IS EVERYTHING
