# Supplementary material for: Art’s hidden topology: A window into human perception
Source: PLoS Comput Biol. 2026 May 14;22(5):e1014156. doi: 10.1371/journal.pcbi.1014156 (PMC13175340; doi:10.1371/journal.pcbi.1014156)
Supplement: S5 Fig — (PDF) [file pcbi.1014156.s005.pdf]

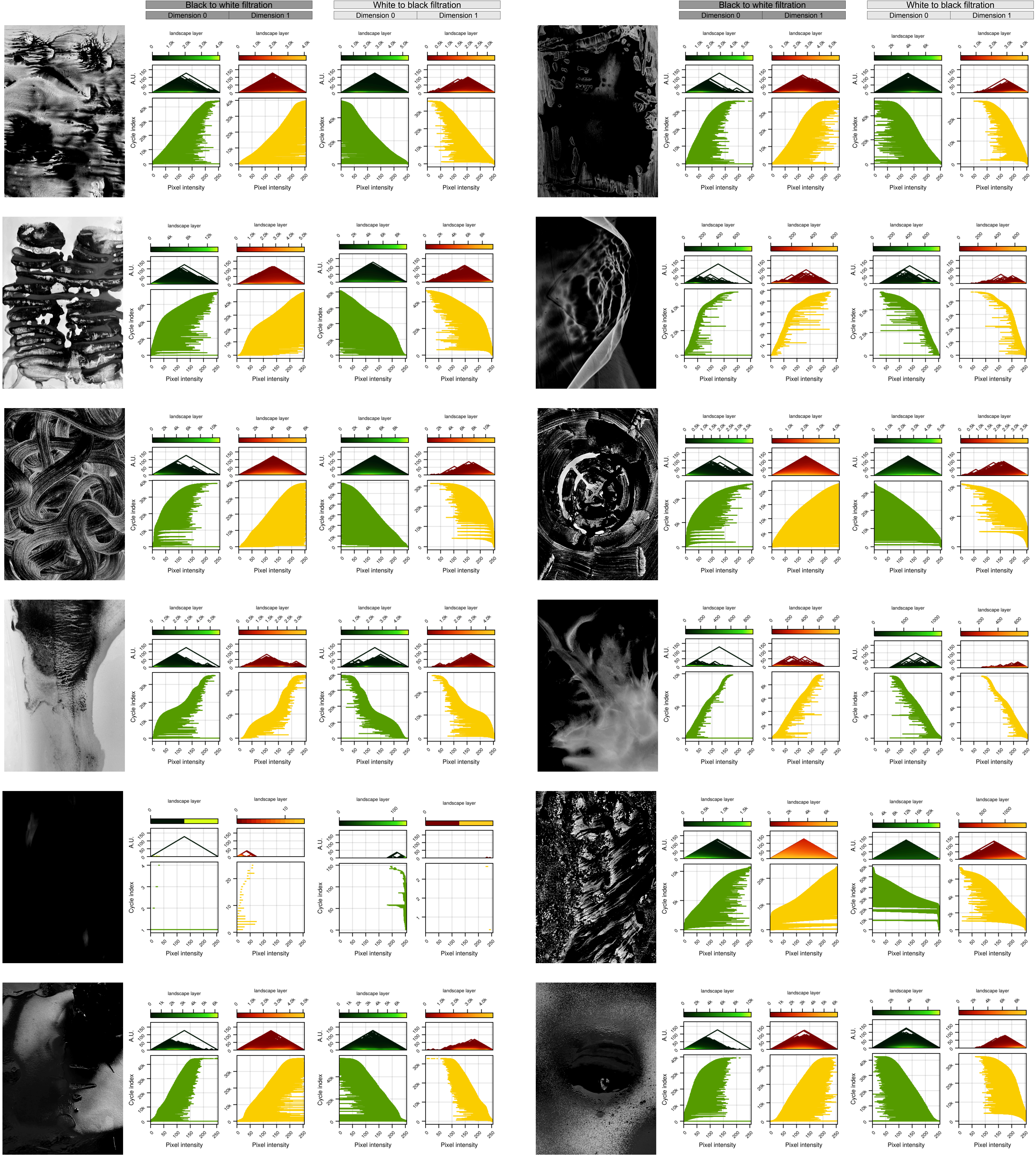

Black to white filtration
Dimension 0
Dimension 1
White to black filtration
Dimension 0
Dimension 1
Black to white filtration
Dimension 0
Dimension 1
White to black filtration
Dimension 0
Dimension 1
landscape layer
A.U.
Cycle index
Pixel intensity
