# Supplementary material for: Art’s hidden topology: A window into human perception
Source: PLoS Comput Biol. 2026 May 14;22(5):e1014156. doi: 10.1371/journal.pcbi.1014156 (PMC13175340; doi:10.1371/journal.pcbi.1014156)
Supplement: S9 Fig — The image used for black-to-white filtration is presented in the first column, top row; the dual filtration is done with the inverse of the original image, with the filtration again going from black to white (this was done so that it is easier to notice starting points, while the results of the filtration are the same after inverting the horizontal axis on the plots). For both images, results are presented in dimensions 0 and 1 in the form of persistence landscapes, barcodes and Betti curves, as indicated with the labels. The results for dimension 1, top row and dimension 0, bottom row, are the same with respect to inverting the horizontal axis. The results for dimension 0, top row, have 2 more cycles, one is the infinite cycle (which contributes to the outermost layer in the landscape), second missing cycle is related to one of the barcodes starting at step 0 and finishing at step 4 (dimension 0, top filtration). (PDF) [file pcbi.1014156.s009.pdf]

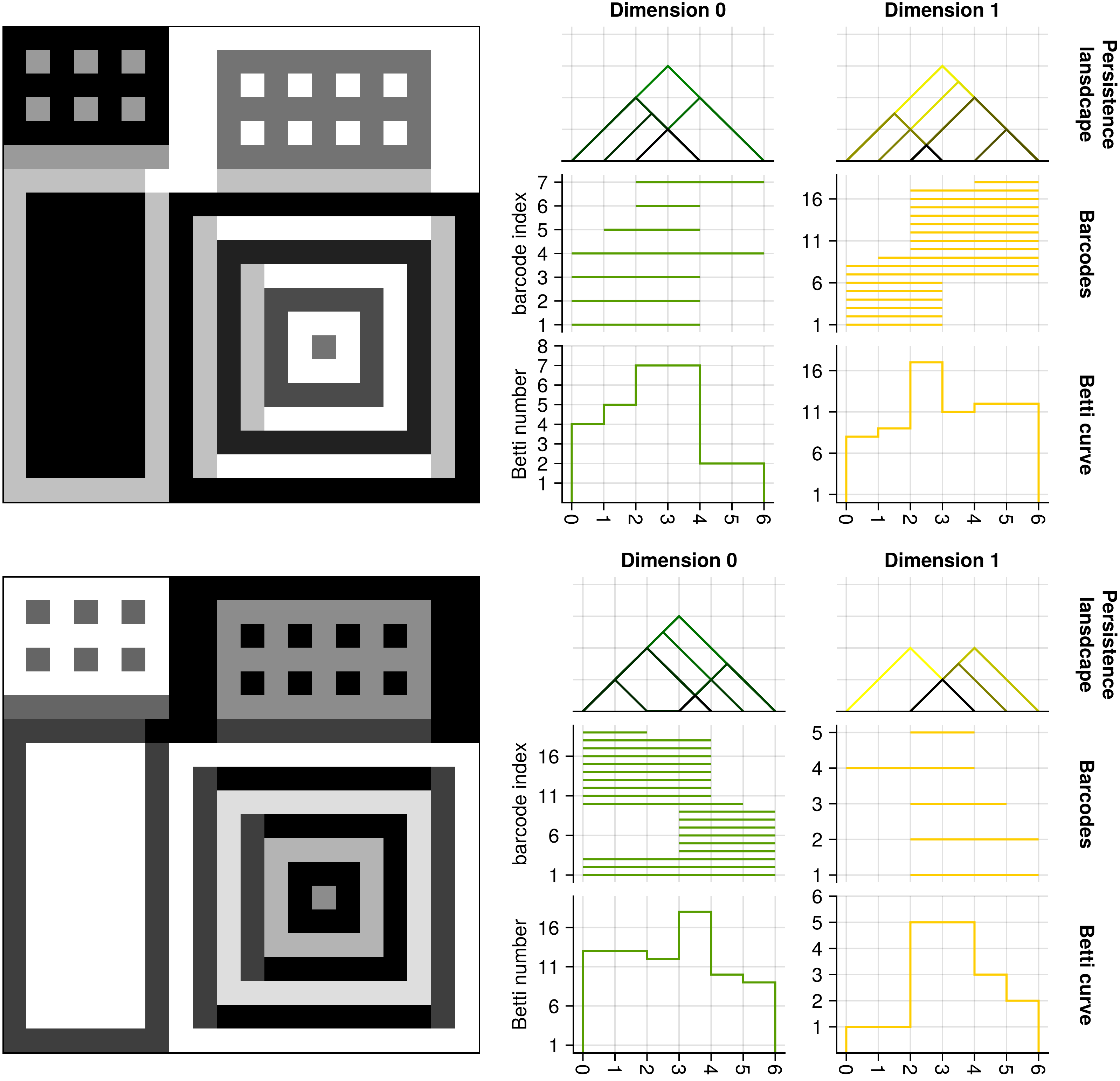

Dimension 0
Dimension 1
Persistence lansdcape
barcode index
Barcodes
Betti number
Betti curve
Dimension 0
Dimension 1
Persistence lansdcape
barcode index
Barcodes
Betti number
Betti curve
