# Supplementary material for: Art’s hidden topology: A window into human perception
Source: PLoS Comput Biol. 2026 May 14;22(5):e1014156. doi: 10.1371/journal.pcbi.1014156 (PMC13175340; doi:10.1371/journal.pcbi.1014156)
Supplement: S10 Fig — To justify the sufficiency of only selecting dimension 1 cycles for our analysis, we computed L1 pairwise distance between all 24 persistence landscapes within each dimension and for both filtrations. The duality between the topological invariants derived from the two filtrations predicts (up to boundary cycles) that these L1 distance matrices will be almost identical. A: filtration from black to white. B: filtration from white to black. Each heatmap shows L1, pairwise differences between the persistence landscapes of all images in dimensions 0 (left) and 1 (right). Every row (or column) in the heatmap corresponds to an image annotated between the matrices. The top 12 labels (coloured orange) are from pseudo-artistic images, and the next 12 labels (coloured green) are from artist images. While not all cycles are reflected in the duality, the overall shape differences captured with L1 distance between landscapes are mirrored when the filtration of the images is inverted. This verifies the expected similarity between the following pairs of matrices: ((1) BW filtration in dimension 0 and WB filtration in dimension 1; (2) BW filtration in dimension 1 and WB filtration in dimension 0). This is a powerful demonstration of the duality. (PDF) [file pcbi.1014156.s010.pdf]

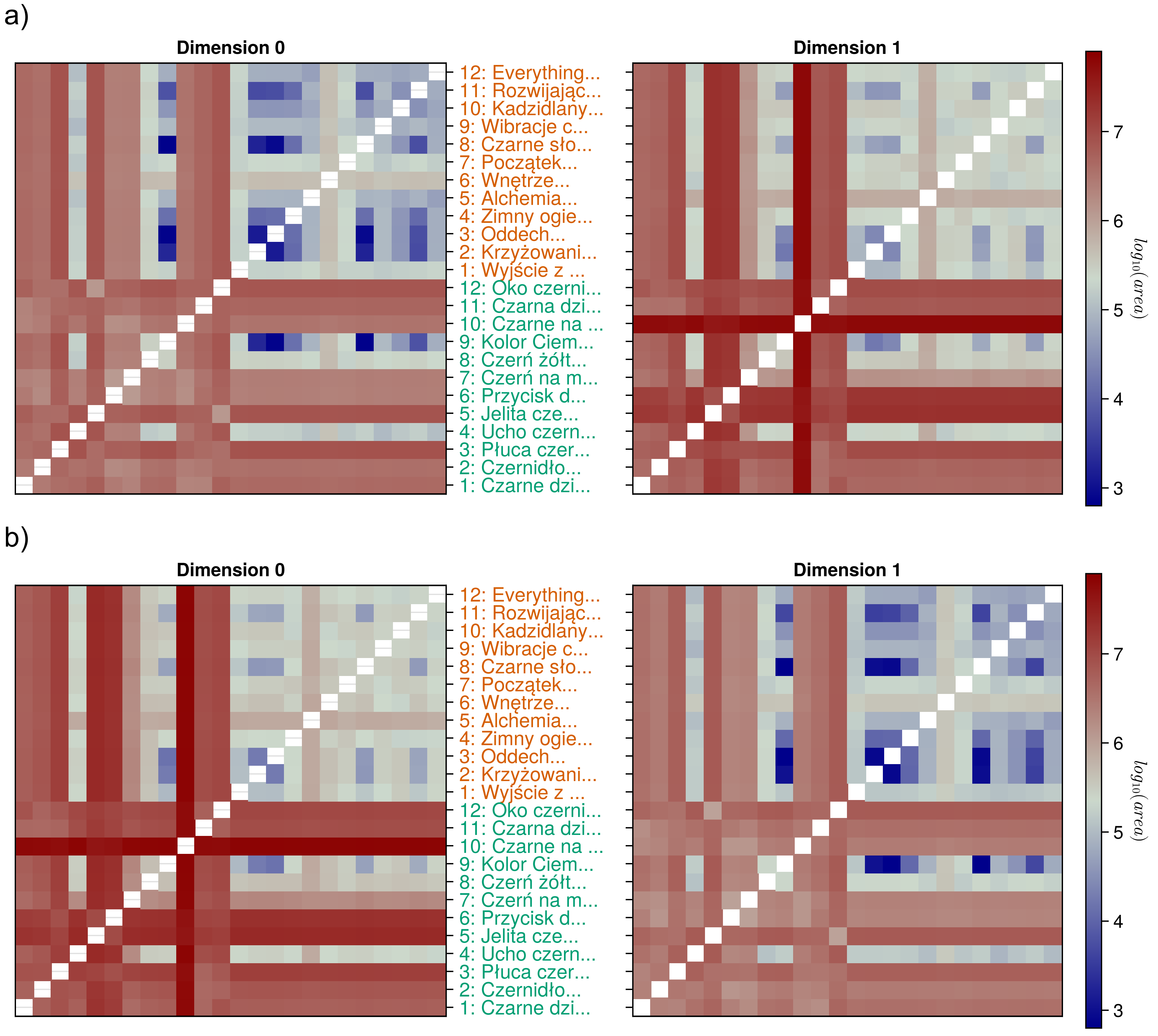

a)
Dimension 0
Dimension 1
12: Everything...
11: Rozwijając...
10: Kadzidlany...
9: Wibracje c...
8: Czarne sło...
7: Początek...
6: Wnętrze...
5: Alchemia...
4: Zimny ogie...
3: Oddech...
2: Krzyżowani...
1: Wyjście z ...
12: Oko czerni...
11: Czarna dzi...
10: Czarne na ...
9: Kolor Ciem...
8: Czerń żółt...
7: Czerń na m...
6: Przycisk d...
5: Jelita cze...
4: Ucho czern...
3: Płuca czer...
2: Czernidło...
1: Czarne dzi...
7
6
5
4
3
$log_{10}(area)$
b)
Dimension 0
Dimension 1
12: Everything...
11: Rozwijając...
10: Kadzidlany...
9: Wibracje c...
8: Czarne sło...
7: Początek...
6: Wnętrze...
5: Alchemia...
4: Zimny ogie...
3: Oddech...
2: Krzyżowani...
1: Wyjście z ...
12: Oko czerni...
11: Czarna dzi...
10: Czarne na ...
9: Kolor Ciem...
8: Czerń żółt...
7: Czerń na m...
6: Przycisk d...
5: Jelita cze...
4: Ucho czern...
3: Płuca czer...
2: Czernidło...
1: Czarne dzi...
7
6
5
4
3
$log_{10}(area)$
