# Supplementary material for: Art’s hidden topology: A window into human perception
Source: PLoS Comput Biol. 2026 May 14;22(5):e1014156. doi: 10.1371/journal.pcbi.1014156 (PMC13175340; doi:10.1371/journal.pcbi.1014156)
Supplement: S33 Fig — ECDF(Mperimeter,U), for: BW filtration (top row), WB filtration (middle row), combined BW and WB filtration (bottom row). (PDF) [file pcbi.1014156.s033.pdf]

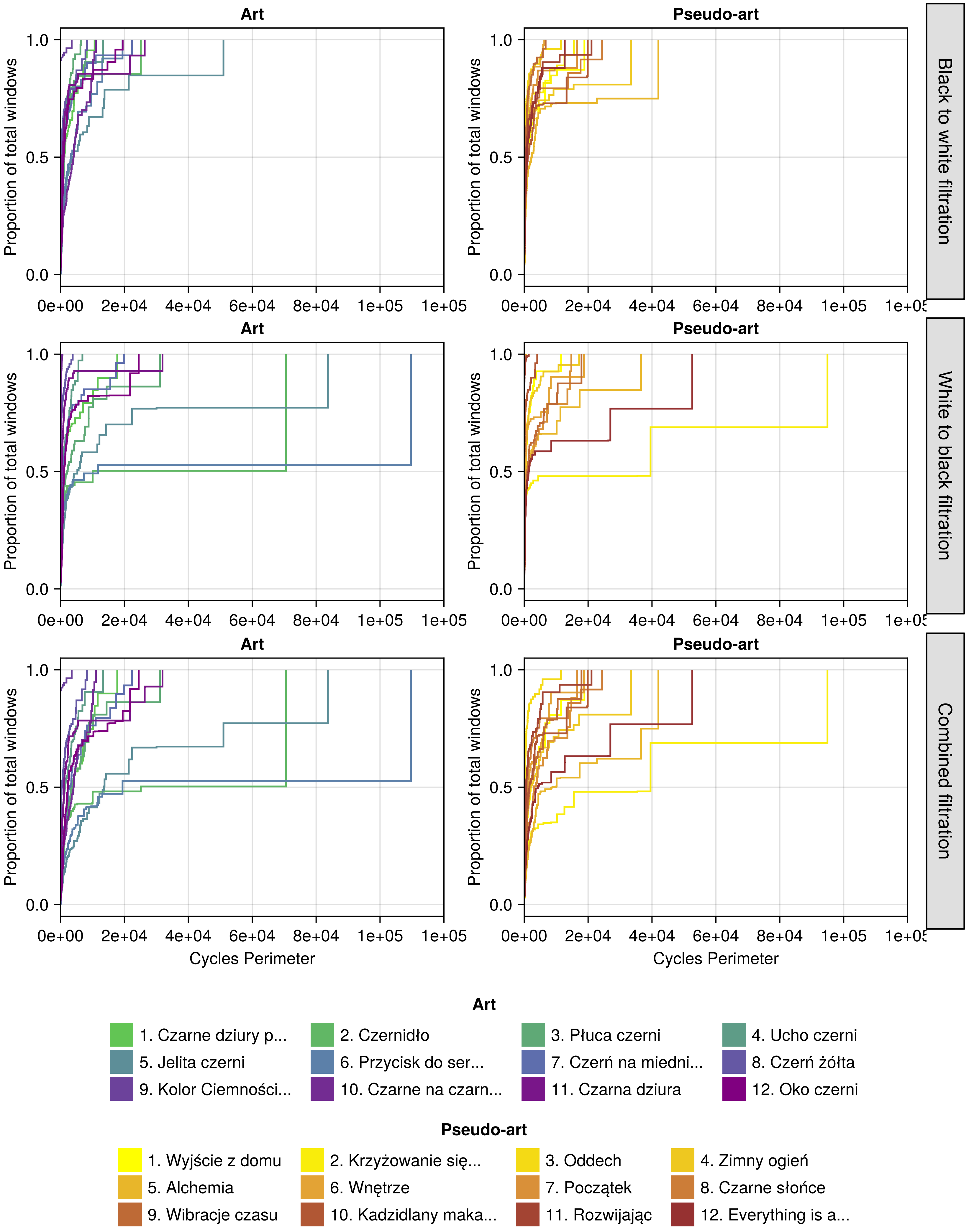

Art
Pseudo-art
Black to white filtration
White to black filtration
Combined filtration
Proportion of total windows
Cycles Perimeter
1.0
0.5
0.0
0e+00
2e+04
4e+04
6e+04
8e+04
1e+05
Art
1. Czarne dziury p...
2. Czernidło
3. Płuca czerni
4. Ucho czerni
5. Jelita czerni
6. Przycisk do ser...
7. Czerń na miedni...
8. Czerń żółta
9. Kolor Ciemności...
10. Czarne na czarn...
11. Czarna dziura
12. Oko czerni
Pseudo-art
1. Wyjście z domu
2. Krzyżowanie się...
3. Oddech
4. Zimny ogień
5. Alchemia
6. Wnętrze
7. Początek
8. Czarne słońce
9. Wibracje czasu
10. Kadzidlany maka...
11. Rozwijając
12. Everything is a...
