# Supplementary material for: Art’s hidden topology: A window into human perception
Source: PLoS Comput Biol. 2026 May 14;22(5):e1014156. doi: 10.1371/journal.pcbi.1014156 (PMC13175340; doi:10.1371/journal.pcbi.1014156)
Supplement: S1 Appendix — (PDF) [file pcbi.1014156.s037.pdf]

# S1 Appendix. EEG study.

## EEG analysis.

To analyse putative differences in brain activity, we used brain connectivity measures applied to the electroencephalograph (EEG) signal. Brain connectivity can be used to estimate how different brain regions interact during cognitive tasks and how these interactions change in response to interventions. In our study, we hypothesised that the two sets of images could challenge subjects with different levels of difficulty and engagement, causing differences in the organisation of brain connectivity patterns. To increase statistical power, all analyses were performed on combined data from both laboratory visits.

To infer EEG brain connectivity, we use statistical dependencies between EEG signals from different brain regions to estimate how different regions of the brain communicate with each other, hence we used the weighted phase lag index (wPLI) [1,2] calculated for all pairs of electrodes. For EEG measurements, the 64-channel BrainAmp amplifiers manufactured by Brain Products GmbH were used. This device operates at a sampling frequency of 1000 Hz.

Signal pre-processing and analyses were performed using MNE-Python [3]The preprocessing procedure included downsampling to 500 Hz and filtering in the 0.2-70 Hz range with a finite impulse response (FIR) filter with a Hamming window automatically adjusted to signal length. The power noise of 50 Hz and its higher harmonics (100 Hz and 150 Hz) were removed using a notch filter. To minimise the effect of muscle artefacts, we applied Independent Component Analysis and, using MNE functions, automatically removed components suspected of muscle origin.

Next, the data were cut into 7-second chunks (epochs) matching the duration of the periods when participants viewed the images. To compensate for the high variability of the EEG and to increase statistical power, we combined recordings from both laboratory visits.

The same preprocessing procedure was also applied to resting-state data collected from both groups prior to any gallery or laboratory visit, except we replaced the 7-second epochs collected while viewing the images with segments of the same duration extracted from the resting state. The resting state data were used to verify that the groups did not differ in EEG features even before the study began.

All analyses were performed separately for each of the following canonical EEG bands (theta: 4-8 Hz, alpha: 8-13 Hz, beta 1: 13-20 Hz, beta 2: 20-30 Hz and gamma: 30-70 Hz). Statistical comparisons of group differences were performed using the mass Mann-Whitney U-tests with FDR correction for multiple comparisons. All comparisons were performed using the full set of electrodes. Using the connectivity matrix, we also performed graph theoretic analyses focused on three global graph parameters, namely global efficiency, modularity and clustering coefficients. The analyses were performed using the Brain Connectivity Toolbox for Python version 0.6.1.

## EEG results.

The initial comparison of the EEG connectivity based on the resting-state signals collected prior to gallery and laboratory visits showed no significant group differences in neither investigated band thus excluding the possibility that possible disparities observed during image viewing stemmed from initial group variations.

The comparison of the group averaged signal correlations while participants were viewing images yielded significant differences in beta1 and gamma bands. In the beta1 band connectivity strength was higher for the participants watching artist images, while in the gamma band correlations were stronger in the pseudo-artistic group. Fig in S1 Fig and Fig in S2 Fig

The graph theoretic analyses performed using the wPLI connectivity matrix also showed significant differences between analysed groups. In the beta 2 band significant differences were found for all three graph measures: global efficiency and modularity appeared to be higher for artist images (diff art-pseudo-art= 0.1943 p= 0.0245 and diff art-pseudo-art=0.0041 p= 0.0158 respectively), while clustering coefficient was lower for artist images (diff art-pseudo-art= -0.0164

p= 0.019. Similar pattern was also found for gamma band where global efficiency was higher for artist images (diff art-pseudo-art = 0.2623, p= 0.0081) and cluster coefficient was lower (diff art-pseudo-art = -0.0257, p= 0.0115). The difference for modularity was not significant p= 0.1077).

# References


1. Vinck M., Oostenveld R., Van Wingerden M., Battaglia F., Pennartz C. M. A. An improved index of phase-synchronization for electrophysiological data in the presence of volume-conduction, noise and sample-size bias NeuroImage. 2011:55(4),1548-1565. doi:10.1016/j.neuroimage.2011.01.055.

2. Hardmeier M., Hatz F., Bousleiman H., Schindler C., Stam C. J., Fuhr P. Reproducibility of Functional Connectivity and Graph Measures Based on the Phase Lag Index (PLI) and Weighted Phase Lag Index (wPLI) Derived from High Resolution EEG PLOS ONE. 2014:9(10),e108648. doi:10.1371/journal.pone.0108648.

3. Gramfort A, Luessi M, Larson E, et al. MEG and EEG Data Analysis with MNE-Python. Frontiers in Neuroscience. 2013;7:267. doi:10.3389/fnins.2013.00267.
