# Supplementary material for: Art’s hidden topology: A window into human perception
Source: PLoS Comput Biol. 2026 May 14;22(5):e1014156. doi: 10.1371/journal.pcbi.1014156 (PMC13175340; doi:10.1371/journal.pcbi.1014156)
Supplement: S2 Appendix — (PDF) [file pcbi.1014156.s038.pdf]

S2 Appendix. Aesthetic Experience Questionnaires

Aesthetic Experience Questionnaire Before the visit to the art gallery

Aesthetic Experience Questionnaire [1] (Translation and adaptation: Marcin Napiórkowski, 2020, Cronbach's $\alpha = .753$).

Note: The questions are presented here exactly as they were presented to the participants that is in Polish; original (English) version of questions is presented in the brackets below each question.

1. Doświadczam szerokiego spektrum emocji.
   (eng. "I experience a wide range of emotions".)
2. Moje emocje zmieniają się w miarę oglądania dzieła sztuki.
   (eng. "My emotions change as I continue to view the work of art".)
3. Czuję się poruszony/a.
   (eng. "I feel moved".)
4. Doświadczam fizycznej reakcji.
   (eng."I experience a physical reaction".)
5. Porównuje dawną kulturę ze sztuką współczesną.
   (eng."I compare the past culture of the art with present-day culture".)
6. Postrzegam dzieło sztuki jako wyraz epoki, w której powstało.
   (eng. "I see the work of art as an extension of its time period".)
7. Staram się umieścić dzieło w jego kontekście historycznym.
   (eng. "I try to place the work of art in its historical context".)
8. Odnoszę je do innych dzieł sztuki.
   (eng. "I relate it to other works of art".)
9. Ważna jest dla mnie kompozycja dzieła.
   (eng. "The composition of a work of art is important to me".)
10. Istotne są dla mnie kolory dzieła.
    (eng. "The colors of the work of art are important to me".)
11. Skupiam się na subtelnych aspektach dzieła.
    (eng. "I experience a wide range of emotions".)
12. Staram się zrozumieć dzieło całościowo.
    (eng. "I focus on the subtle aspects of the work of art".)
13. Staram się zrozumieć, co artysta chciał przekazać.
    (eng. "I try to understand the work completely".)
14. Zdobywam nowe intuicje o samym dziele.
    (eng. "I try to understand what the artist is trying to communicate".)
15. Zyskuję nowe spostrzeżenia dotyczące dzieła.
    (eng."I gain new insights about the work of art itself".)

16. Mam jasne wyobrażenie, czego szukać, kiedy patrzę na dzieło sztuki.

    (eng. "I have a clear idea of what to look for when viewing the work of art".)

17. Zwykle czuje, kiedy moje wyobrażenia na temat dzieła sztuki są poprawne.

    (eng."I usually feel that my thoughts on the work of art are correct".)

18. Czuje, że jestem w stanie zrozumieć dzieło sztuki.

    (eng. "I feel I am able to understand the work of art".)

19. Tracę poczucie czasu, gdy patrzę na dzieło sztuki.

    (eng. "I lose track of time when I view the work of art".)

20. Zatapiam się w swoich myślach, gdy patrzę na dzieło sztuki.

    (eng. "I get lost in thought when I view the work of art".)

21. Jestem całkowicie skupiony/a na oglądaniu dzieła.

    (eng. "I am completely focused on viewing the work of art".)

22. Doświadczenie oglądania sztuki jest dla mnie satysfakcjonujące.

    (eng. "The experience of viewing the work of art is rewarding to me".)

Aesthetic Experience in the Art Gallery - Exhibition Evaluation.

Translation and adaptation: Marcin Napiórkowski, Cronbach's $\alpha = .91$, Paper-and-pencil version.

Question: 'Please indicate how much you agree with the following statements regarding your experience of the exhibition. Use a 5-point Likert scale (1 = strongly disagree, 5 = strongly agree)'.

Note: The questions are presented here exactly as they were presented to the participants, that is in English- this was an experiment design choice. For completeness, translation to Polish in include in brackets.

1. It was easy for me to notice and recognize the meaning of individual elements (color, composition, etc.) in the exhibition.

    (pl. "Łatwo mi było dostrzec i rozpoznać znaczenie poszczególnych elementów (kolor, kompozycja itp.) wystawy.".)

2. The exhibition evoked strong emotions in me; I felt moved.

    (pl. "Wystawa wzbudziła we mnie silne emocje, jestem poruszony/a.")

3. I understood the author's intentions. I knew what the exhibition was meant to communicate.

    (pl. "Odczytuję intencje autora. Wiem, co wystawa ma przekazywać.")

4. I was able to place the exhibition in a broader context — I recognized its connection to art history and other works.

    (pl. "Potrafię umieścić wystawę w kontekście – dostrzegam jej związek z historią sztuki i innymi dziełami.")

5. During the exhibition, I knew what to focus on; I understood what I was seeing.

    (pl. "Na wystawie wiedziałem/łam, na czym się skupić, rozumiałem/łam to, co widzę.")

6. I was fully focused during the exhibition, lost track of time, and experienced a sense of pleasure.

    (pl. "Na wystawie byłem/łam w pełni skupiony/a, traciłem/łam poczucie czasu, przeżywałem/łam przyjemne doświadczenie.")

Aesthetic Experience in the Laboratory

Evaluation of Individual Artworks Translation and adaptation: Marcin Napiórkowski, Cronbach's $\alpha = .90$, Computer-based version.

Question: 'Please rate the following statements about your experience while viewing individual artworks'. Responses were recorded using a computerized slider scale with five anchor points: 0% (strongly disagree), 25%, 50%, 75%, 100% (strongly agree).

Note: The questions are presented here exactly as they were presented to the participants, that is in English- this was an experiment design choice. For completeness, translation to Polish in include in brackets.

1. It is easy for me to notice and recognize the meaning of individual elements (color, composition, etc.).

   (pl. "Łatwo mi jest dostrzec i rozpoznać znaczenie poszczególnych elementów (kolor, kompozycja itp.)".)

2. I feel strong emotions; I am moved.

   (pl. "Odczuwam silne emocje, jestem poruszony/a.".)

3. I understand the artist's intentions. I know what the artwork is meant to convey.

   (pl. "Odczytuję intencje autora. Wiem, co dzieło ma przekazywać".)

4. I am able to place the artwork in context — I see its connection to art history and other works.

   (pl. "Potrafię umieścić to dzieło w kontekście – dostrzegam jej związek z historią sztuki i innymi dziełami".)

5. I know what to focus on; I understand what I am seeing.

   (pl. "Wiem, na czym się skupić, rozumiem to, co widzę".)

6. I am fully focused, I lose track of time, and I experience a pleasurable state.

   (pl. "Jestem w pełni skupiony/a, tracę poczucie czasu, przeżywam przyjemne doświadczenie".)

References

1. Wanzer DL, Finley KP, Zarian S, Cortez N. Experiencing Flow While Viewing Art: Development of the Aesthetic Experience Questionnaire. Psychology of Aesthetics, Creativity, and the Arts. 2020;14(1):113–124. doi:10.1037/aca0000203.
