# Supplementary material for: Art’s hidden topology: A window into human perception
Source: PLoS Comput Biol. 2026 May 14;22(5):e1014156. doi: 10.1371/journal.pcbi.1014156 (PMC13175340; doi:10.1371/journal.pcbi.1014156)
Supplement: S3 Appendix — We also discuss the proof of Alexander duality in more detail. (PDF) [file pcbi.1014156.s039.pdf]

S3 Appendix. **Persistent Homology** Here we provide more details and definitions for Persistent Homology, Cubical Complexes, Betti curves and Persistence Landscapes. We also discuss the proof of Alexander duality in more detail.

Persistent Homology, Cubical Complexes and Betti Curves.

For each image I we build a cubical complex $C_j(I)$ for each step of the filtration $j \in [0, M]$ ($M = 255$) using the procedure described in the main text, where the one-dimensional filtration parameter used is pixel intensity. Hence, we have the following chain of inclusions (a family of cubical complexes) to which the persistence algorithm [1] is applied:

$$C_0(I) \subset C_1(I) \subset \ldots \subset C_r(I) \subset C_{r+1}(I) \subset \ldots \subset C_M(I). \quad (1)$$

The ith Betti curve of $C(I) = \{C_0(I), C_1(I), \ldots, C_M(I)\}$ is defined by:

$$\beta_i(j) = \beta_i(C_j(I)) := \operatorname{rank} H_i(C_j(I), k),\ \ j \in [0, M], \quad (2)$$

where $H_i(C_j(I), k)$ is the ith homology group of $C_j(I)$ with coefficients in k (k can be any field; the software implementation that we used, took $k = \mathbb{Z}/2\mathbb{Z}$.):

$$H_i(C_j(I), k) := \frac{\ker \partial_i}{\operatorname{im} \partial_{i+1}}. \quad (3)$$

The maps ∂_i are the standard boundary maps. The inclusion maps $\iota_r : C_r(I) \hookrightarrow C_{r+1}(I)$ induce maps on the homology $(\iota_r)_i : H_i(C_r(I), k) \to H_i(C_{r+1}(I), k)$. These induced maps allow us to follow cycle representatives of homology classes as they evolve through the filtration, from one complex to the next. In particular, we note when they are 'born' 'birth time' b (the value of the pixel intensity when it first appears) and when they die 'death time' d (the value of the pixel intensity when it ceases to exist).

The Persistent Homology (PH) classes of the cubical complexes derived from an image can therefore be summarised by a collection of persistence intervals or 'barcodes'.

Sketch proof of duality.

Let $\tilde{C}(I)$ be the reverse filtration of $C(I)$ where the pixel intensity order is reversed. The cubical complexes for $\tilde{C}(I)$ at step f are complementary to those of $C(I)$ at step $M - 1 - f$ of the filtration. If the grid of the underlying image is embedded onto an S^2 (via 1-point sphere compactification), by Alexander duality (or simple observation in this case), for every $[\tau] \in H_0(C_j(I), k)$ there is a corresponding complementary class $[\tilde{\tau}] \in H_1(\tilde{C}_{M-1-j}(I), k)$. The birth time of the corresponding PH representative of τ, b_τ is related to death time of PH representative corresponding to $\tilde{\tau}$, $d_{\tilde{\tau}}$ via $b_\tau = M - 1 - d_{\tilde{\tau}}$ and vice versa. In our situation, where the image does not lie on a sphere, this relationship holds everywhere except on the boundary.

Persistence landscapes.

For the purpose of comparative study, instead of using standard barcode representation, the results from PH were represented as persistence landscapes [2]. For each cycle of a given image and dimension, a piecewise linear function $f_{(b,d)} : \mathbb{R} \to [0, \infty)$ was defined as:

$$f_{(b,d)} = \begin{cases} 0 & \text{if } x \notin [b, d] \\ x - b & \text{if } x \in [b, \frac{b+d}{2}] \\ -x + d & \text{if } x \in (\frac{b+d}{2}, d] \end{cases} \quad (4)$$

The intuition behind this formula is to build an upward-pointing, right-angled isosceles triangle with the base positioned between birth and death. Having this conversion of a multiset of n persistence barcodes $\{(b_i, d_i)\}_{i=1}^{n}$ into functions, it is now possible to construct a persistence landscape as a set of functions $\lambda_k : \mathbb{R} \to \mathbb{R}$ such that $\lambda_k(x)$ is the kth largest value of $\{f_{(b,d)}(x)\}_{i=1}^{n}$ and $\lambda_k(x) = 0$ when there is no kth largest value (The index k in $\lambda_k(x)$ denotes the layer of the landscape.

When an operation on a few landscapes is performed (For example averaging for a group or distance computation between a pair of landscapes), the operation is applied to each layer separately. Therefore, the (mean) average landscape is the landscape that results from taking the mean of each layer separately; hence, the average landscape $\tilde{\lambda}$ is defined as a collection of functions $\tilde{\lambda}_k(x)$ for each of its layers indexed by k:

$$\tilde{\lambda}_k(x) = \frac{1}{n} \sum_{i=1}^{n} \lambda_k^i(x) \tag{5}$$

where n is total number of landscapes to be averaged and λ_k^i is as defined as the kth layer for the ith landscape.

The L^1 distance between two landscapes is computed as the total sum of the absolute value of the differences between the two landscapes in each layer separately, where the distance within a layer is the difference of heights of every point where either of the layers is defined. If one of the landscapes has more layers than the other, the missing layers are considered to be zero-height layers. The L^1 distance between landscapes f and g is thus defined as:

$$||f - g|| = \sum_{k=1}^{max(K,K')} \int ||f_k - g_k|| \tag{6}$$

where K and K' are the maximum numbers of layers for landscapes f and g respectively. The norm $||f_k - g_k|| = \int |f_k(x) - g_k(x)|dx$ can be computed as the sum of integrals over the landscape intervals.

Those measures were used to compare the topology of the images from the two sets under consideration.

Persistence homology was computed using a filtration step size of 5-pixel intensity units as the human resolution is restricted to 5 points (Petrov, Y. 2005) [3]. Additional tests of filtration steps: 0, 2, 5, and 10 showed that only threshold 10 decreased the total landscape area (not shown). PH derived from cubical complexes was computed using the DIPHA library and the persistence landscape toolbox [2] rewritten in Julia for this purpose.

References

1. Carlsson G, Zomorodian A. The Theory of Multidimensional Persistence. Discrete & Computational Geometry. 2009;42(1):71–93. doi:10.1007/s00454-009-9176-0.

2. Bubenik P, Dłotko P. A Persistence Landscapes Toolbox for Topological Statistics. Journal of Symbolic Computation. 2017;78:91–114. doi:10.1016/j.jsc.2016.03.009.

3. Petrov Y. Luminance Correlations Define Human Sensitivity to Contrast Resolution in Natural Images. Journal of the Optical Society of America A. 2005;22(4):587. doi:10.1364/JOSAA.22.000587.
