# Supplementary material for: Art’s hidden topology: A window into human perception
Source: PLoS Comput Biol. 2026 May 14;22(5):e1014156. doi: 10.1371/journal.pcbi.1014156 (PMC13175340; doi:10.1371/journal.pcbi.1014156)
Supplement: S4 Appendix — (PDF) [file pcbi.1014156.s040.pdf]

S4 Appendix. Heatmaps, feature maps and operations on ECDF

Generation of gaze heatmaps from eye-tracking data.

Given eye movement data from viewing a single image, fixation sequences lasting over 75 ms were extracted and converted into gaze heatmaps using the Python package *Gaze Point Heat Map* [1]. This outputs a heatmap in the form of a 1434×2048, matrix. The heatmap thus obtained is then normalised and converted into a probability distribution. We shall specify each gaze distribution by a matrix G, where $\sum_{i,j} G_{i,j} = 1$, where i and j index the rows and columns of a chosen grid size. The grid chosen can be coarse or fine. We shall choose a grid size equal to that of feature maps to obtain distributions of topological descriptors, as described below.

Feature map processing.

An image overlaid with cycle representatives of dimension 1 persistent homology classes (visualisation of the representative cycle at its birth) as in S12 FigB, can be covered by a square grid. For convenience, we have chosen a grid with uniform fixed-sized non-overlapping windows.

An image overlaid with cycle representatives of dimension 1 persistent homology classes (visualisation of the representative cycle at its birth) as in S12 FigB, can be covered by a square grid. For convenience, we have chosen a grid with uniform fixed-sized non-overlapping windows.

A feature map can then be specified by a matrix M where, $M_{i,j}$ is the local measure of a chosen topological descriptor in the square in the ith row and the jth column of the grid, For example for cycle density, $M_{i,j}$ is the total number of cycles in the (i, j)th grid square (see S1 Table for the other examples used in this paper).

Composite feature maps can be constructed by combining feature map matrices from both filtrations, BW and WB, For example for maximal persistence we take $M_{i,j} := \max(M^{BW}_{i,j}, M^{BW}_{i,j})$, where M^{BW} and M^{WB} are the maximum persistence feature maps for the BW and WB filtration respectively. Other feature maps used in this study are summarised in Supplementary Materials S1 Table.

Given a feature map M of topological descriptors we can define two types of probability distributions supported on the grid with n_{row} rows and n_{col} columns :

$$P(m = M_{i,j}) = U_{i,j} := \frac{1}{g},\ 1 \leq i \leq n_{row},\ 1 \leq j \leq n_{col}, \tag{7}$$

where $g = n_{row}n_{col}$ is the total number of windows in the grid. We shall denote the above as the distribution that is intrinsic to the image.

The distribution,

$$P(m = M_{i,j}) = G_{i,j},\ \forall i, j \tag{8}$$

is the distribution relative to a person's gaze map, G, where $\sum_{i,j} G_{i,j} = 1$. Given a gaze map G we can also define a person's 'not looking' or complement uniform gaze map $\tilde{G}_{i,j}$ to be:

$$\tilde{G}_{i,j} := \frac{\mathbb{1}(G_{i,j} = 0)}{\sum_{l,m} \mathbb{1}(G_{l,m} = 0)},\ \forall i, j, \tag{9}$$

where $\mathbb{1}$ is the indicator function and the denominator gives the total number of grid squares in which the gaze map G is zero. Hence, $P(m = M_{i,j}) = \tilde{G}_{i,j},\ \forall i, j$, is the distribution of where the person was not looking.

In our analysis, we compare the cumulative distribution functions of the above three types of probability distributions. As these CDFs are derived from data, we shall refer to them as Empirical Cumulative Distribution Functions (ECDFs).

Note that for some $(i,j) \neq (k,l)$, it may be that $M_{i,j} = M_{k,l}$ in the above definitions. The intrinsic distribution can be considered the distribution relative to the uniform gaze $\sum_{i,j} U_{i,j} = 1$ that looks equally at all parts of the image, like a scanner rather than a human viewer of art.

The window size used for feature maps in this study was 51×51, $H \times W$. Other sizes were also tested, yielding similar results - as shown in S30 Fig.

Operations on ECDFs.

Given a (composite) topological descriptor M and a gaze map G, we denote the probability distribution defined in Equation 8, $\mathcal{D}(M,G)$, and the distribution intrinsic to the image defined in Equation 7 as $\mathcal{D}(M,U)$. The corresponding ECDFs will be denoted by $EDCF(M,G)$ and $ECDF(M,U)$.

Given an image k with a feature map M_k, a subject s with gaze distribution G_s, we can compare the distributions using their ECDFs by computing mean error (ME) and mean squared error (MSE) in the usual way as follows:

$$ME_{k,s} = \frac{1}{X} \sum_{x=x_{min}}^{x=x_{max}} ECDF(M_k,U)(x) - ECDF(M_k,G_s)(x), \tag{10}$$

where $x \in [x_{min}, x_{max}]$, $X = x_{max} - x_{min}$ is the range of values of the topological descriptor for which $0 \leq ECDF(M_k,U)(x) < 1$, in other words, the width of the curve, $ECDF(M_k,U)$.

Analogously the Mean Squared Error is given by:

$$MSE_{k,s} = \frac{1}{X} \sum_{x=x_{min}}^{x=x_{max}} (ECDF(M_k,U)(x) - ECDF(M_k,G_s)(x))^2. \tag{11}$$

The full set of ECDFs, for each image k, feature map M and participant s, $ECDF(M_k,U)$ ('intrinsic'), $ECDF(M_k,G_s)$ ('looking'), $ECDF(M_k,\tilde{G_s})$ ('not looking') can be found in S34 Fig, S35 Fig, S36 Fig..

References

1. Roeddiger T. Gaze Point Heat Map; 2024. https://github.com/TobiasRoeddiger/GazePointHeatMap.
