# Supplementary material for: Art’s hidden topology: A window into human perception
Source: PLoS Comput Biol. 2026 May 14;22(5):e1014156. doi: 10.1371/journal.pcbi.1014156 (PMC13175340; doi:10.1371/journal.pcbi.1014156)
Supplement: S5 Appendix — (PDF) [file pcbi.1014156.s041.pdf]

S5 Appendix. The effect of image resizing and histogram manipulations.

The effect of image resizing.

All images used in the study were 1434 pixels wide and 2048 pixels tall. Since the choice of image size was arbitrary, we tested how much topological properties changed with the change in size of the images.

The effect of changing image size on the area under the persistence landscapes for the 24 images (as shown in the caption) is shown in the area-under-landscape space, where the x and y coordinates are areas under the landscape in dimensions 0 and 1, respectively.

As shown in Fig , all of the images from both exhibitions were upscaled or downscaled while preserving the image's aspect ratio (image sizes are shown in the upper part of each legend). The upscaling of the images did not change the topological properties significantly- for both groups, the markers occupy the same space. For downscaling, however, the area under the persistence landscape for both data sets is decreasing. It is important to note that at every level of resizing, the relative location of both datasets in the area area-under-landscape space was preserved- the artistic images have higher area-under-landscape than the pseudo-artistic images (except for 3 cases- image number 9 being significantly lower than any other image, and images 4 and 8 being very close to the pseudo-artistic images).

It should be noted that for most of the images, downsizing by a factor of 4 (resulting in image size 512×359) did not change the area-under-landscape by less than one order of magnitude.

The effect of image histogram manipulation on persistent homology.

Below, we demonstrate how manipulating the image intensity histogram affects persistence landscapes. We demonstrate three types of histogram manipulations, two of which were tested with several parameters (all details are presented in S2 Table.

S24 Fig and S25 Fig shows how the image and its persistence properties (persistence landscapes, barcodes, Betti curves) are changing for the tested transformation.

Importantly, the relation between images is preserved- this is shown in S28 Fig and S29 Fig, where we demonstrate the $L1$ distance computed between all pairs of images, after the transformation was applied to all images independently. In all cases, the distance between pseudo-art images was lower than for art images.
