# Supplementary material for: Art’s hidden topology: A window into human perception
Source: PLoS Comput Biol. 2026 May 14;22(5):e1014156. doi: 10.1371/journal.pcbi.1014156 (PMC13175340; doi:10.1371/journal.pcbi.1014156)
Supplement: S6 Appendix — (PDF) [file pcbi.1014156.s042.pdf]

S6 Appendix. Titles of artworks by Richter, Kandinsky, Rothko, Malevich, Jarema, and Pollock.

Gerhard Richter's artworks:

1. 944-954 23711 image
2. Abstract Painting 1987
3. Abstract Painting skin
4. Abstrakter Bild (1992)780-1-76
5. Bach 1 1992
6. "lbbw ks claudius ii 1986 b 1600x900 vn image w1600 m"
7. Abstract painting 1992
8. Abstract painting 809-3
9. "Ohne Titel" 1991
10. Peinture Abstraite 1992 moma
11. Struktur 1989
12. Wald 2005

Wassily Kandinsky's artworks:

1. Composition X, 1939
2. Transverse Line, 1923
3. Yellow-Red-Blue, 1925
4. Untitled 1916
5. Untitled (First Abstract Watercolor), 1910
6. Im Grau, 1919
7. Composition IV, 1911
8. Composition IX, 1936
9. Composition VIII, 1923
10. Composition VII, 1913
11. Composition VI, 1913
12. Composition V, 1911

Mark Rothko's artworks:

1. 1952
2. Black on Dark Sienna on Purple 1960
3. Green Blue green 1969
4. No 301 1959
5. No 46 1957
6. No 9 1954
7. Nr 61 1963
8. Purple Brown 1957
9. Red and Brown 1957
10. untitled 1968
11. untitled 1970
12. Yellow and Orange 1949

Kazimir Malevich's artworks:

1. Dynamic Suprematism

2. Black square and red square 1915 trivium art history
3. Supremus no 50 1915 trivium art history
4. Malevitj Hieratic Suprematist Cross
5. Mystic Suprematism
6. suprematic painting, 1918
7. Suprematism, 18th Construction
8. Suprematism, 1915
9. Suprematism No 55 Spherical Evolution of a Plane 1917
10. Suprematist Composition - Airplane Flying 1915
11. Untitled ca. 1916
12. White on white 1918

Maria Jarema's artworks:

1. FILTRY 1958 mat prom 7 1200x1460
2. KOMPOZYCJA 25217
3. KOMPOZYCJA II POSTACIE 314 1
4. Penetracje 1956
5. PENETRACJE 1958 mat prom 6
6. PENETRACJE I 25204
7. PENETRACJE VII mat prom 2 1200x1018
8. PENETRACJE X 25218
9. RTYM I 1957 mat prom 5 1200x1764
10. Ryrtm VII
11. WYRAZY 06 spectra 37 net Starak
12. WYRAZY IV mat prom 3 1200x838

Jackson Pollock's artworks:

1. Autumn rhythm
2. Birth
3. Convergence
4. Mural on Indian Red Ground
5. Number 1
6. Number 11
7. Number 14
8. Number 23
9. Number 31
10. Number 5
11. Shimmering substance
12. Yellow Islands
