# Supplementary material for: Art’s hidden topology: A window into human perception
Source: PLoS Comput Biol. 2026 May 14;22(5):e1014156. doi: 10.1371/journal.pcbi.1014156 (PMC13175340; doi:10.1371/journal.pcbi.1014156)
Supplement: S1 Table — (PDF) [file pcbi.1014156.s043.pdf]

S1 Table. Feature maps, where $F \in \{BW, WB\}$ is the filtration.

Feature map matrix	Definition of matrix element
$M^F_{density}$	number of distinct cycles per grid square
$M^F_{persistence}$	maximum persistence of cycles that occur within a grid square
$M^F_{perimeter}$	maximum perimeter length of cycles that occur within a grid square
Combined Density $M_{density}$	$M_{density} := M^{BW}_{density} + M^{WB}_{density}$
Maximum Persistence $M_{persistence}$	$M_{persistence} := \max(M^{BW}_{persistence}, M^{WB}_{persistence})$
Maximum Perimeter $M_{perimeter}$	$M_{perimeter} := \max(M^{BW}_{perimeter}, M^{WB}_{perimeter})$
