# Supplementary material for: Art’s hidden topology: A window into human perception
Source: PLoS Comput Biol. 2026 May 14;22(5):e1014156. doi: 10.1371/journal.pcbi.1014156 (PMC13175340; doi:10.1371/journal.pcbi.1014156)
Supplement: S2 Table — (PDF) [file pcbi.1014156.s044.pdf]

S2 Table. Overview of image contrast adjustment transformations, tested parameter configurations, and their short descriptions [1].

Transformation Type	Tested parameters	Description
Contrast stretching	threshold = 0.5, slope = 0.5	Enhances image contrast by stretching the intensity values around a specified threshold using a sigmoid-like function. The slope parameter controls the steepness of the transition.
	threshold = 0.5, slope = 1.5	
	threshold = 1.0, slope = 1.0	
	threshold = 1.5, slope = 1.0	
Linear stretching	a = 0.2, b = 0.8	Adjusts the dynamic range of the image linearly. It maps the original intensity range $[A, B]$ to a new range $[a, b]$.
Gamma correction	gamma = 0.25	Applies a nonlinear transformation to adjust image brightness. A gamma value less than 1 brightens the image, while a value greater than 1 darkens it.
	gamma = 0.5	
	gamma = 2.0	

References

1. Szpak ZL, Chen J, Holy T. ImageContrastAdjustment.Jl: A Julia Package for Enhancing and Manipulating Image Contrast.; 2023. Available from: https://github.com/JuliaImages/ImageContrastAdjustment.jl/tree/master.
