# Supplementary material for: Art’s hidden topology: A window into human perception
Source: PLoS Comput Biol. 2026 May 14;22(5):e1014156. doi: 10.1371/journal.pcbi.1014156 (PMC13175340; doi:10.1371/journal.pcbi.1014156)
Supplement: S3 Table — (PDF) [file pcbi.1014156.s045.pdf]

S3 Table. Statistics for Alexander duality violation distributions..

	$\mathbf{BW_{dim0}}$ **-** $\mathbf{WB_{dim1}}$				$\mathbf{WB_{dim0}}$ **-** $\mathbf{BW_{dim1}}$			
**Measure**	Kot	Pseudo	All art	All pseudo	Kot	Pseudo	All art	All pseudo
Mean	0.4285	0.2764	0.4193	0.1372	0.3198	0.0967	0.3710	0.1576
Median	0.3152	0.1615	0.3988	0.1224	0.2395	0.0960	0.3499	0.1217
Std	0.4370	0.2519	0.2171	0.0678	0.2752	0.0419	0.1668	0.1509
p	0.078		< 0.001		< 0.001		< 0.001	
